# In search for optimal induction chemotherapy for advanced nasopharyngeal cancer: Standard dosing of Docetaxel, Platinum, and 5-Fluorouracil (TPF) followed by chemoradiation

**DOI:** 10.1371/journal.pone.0276651

**Published:** 2023-02-02

**Authors:** Michelle Jun, Harlan Pinto, Quynh-Thu Le, Andrew Quon, Wendy Hara, Jessie Coty, Alex McMillan, Rong Lu, Elzbieta Winters, Ruth Lira, A. Dimitrios Colevas

**Affiliations:** 1 Stanford Cancer Institute, Stanford University, Stanford, California, United States of America; 2 Division of Medical Oncology, Stanford Cancer Center, Stanford University, Stanford, California, United States of America; 3 Department of Radiation Oncology, Stanford Cancer Center, Stanford University, Stanford, California, United States of America; 4 Department of Nuclear Medicine & Molecular Imaging, Stanford University Medical Center, Stanford University, Stanford, California, United States of America; 5 Stanford Health Care, Stanford, California, United States of America; 6 Department of Biomedical Data Science, School of Medicine, Stanford University, Stanford, California, United States of America; 7 Quantitative Sciences Unit, School of Medicine, Stanford University, Stanford, California, United States of America; University of Wisconsin, UNITED STATES

## Abstract

**Objectives:**

A phase II = design is used to evaluate the efficacy and feasibility of full dose docetaxel, platinum, and 5-fluorouracil (TPF) in a sequential chemoradiation treatment locally advanced (LA) or oligometastatic (OM) NPC patients.

**Materials and methods:**

Twenty patients with LANPC (M0 cohort) and six patients with OMNPC (M1 cohort) received induction standard dose T (75 mg/m2) P (75 mg/m2) F (750 mg/m2 IVCI x 5days) x 3 followed by weekly cisplatin (40 mg/m2) or carboplatin (AUC 1.5) x 6 concurrent with radiation therapy of 70 Gy over 6.5–7 weeks. The first five patients received bevacizumab as part of an exploratory objective of hypoxia modification using correlative fluoromisonidasole (^18^F-MISO) PET CT scanning.

**Results:**

The ^18^F-MISO imaging failed to reveal adequate levels of baseline hypoxia necessary to evaluate for changes with chemotherapy and bevacizumab. Ninety percent of M0 patients and 83% of M1 patients received the full-intended TPF and radiation dose. Eighty-five percent of M0 patients and all M1 patients received at least 60% of the full-intended concurrent platinum dose. The 2-year progression free survival (PFS) rate for the M0 cohort was 90% (95% CI: 77.8%– 100%), and was sustained at 5 years. The 2-year PFS rate for the M1 cohort was 66.7% (95% CI: 37.9%– 100%). The 2-year overall survival (OS) rates for the M0 and M1 cohorts were 100% and 83.3% (95% CI: 58.3%– 100%), respectively. At five years, OS was 94.4% for the M0 cohort.

**Conclusion:**

Administration of standard-dose TPF as induction chemotherapy in this NPC patient population is both feasible and effective when coupled with definitive concurrent chemoradiation.

**ClinicalTrials.gov identifier:**

NCT00896181.

## Introduction

The treatment of locoregionally advanced nasopharyngeal carcinoma (LRNPC) has been evolving rapidly. Starting with the seminal trial led by Al Sarraf [1], it has become clear that the combination of chemotherapy and radiation is superior to radiation alone in these patients. The timing of additional chemotherapy with respect to radiation and the optimal concurrent chemoradiation regimen remains unknown. Over the past several decades, improved radiation techniques have been associated with substantial improvements in local and regional disease control. A number of investigators have debated the role of adjuvant chemotherapy with most studies showing minimal, if any, benefit in the unselected LRNPC population [2, 3]. One attempt to address this issue has been risk stratification using blood EBV DNA as a biomarker. Several studies are underway, including NRG Oncology HN 001 [4], which asks if this biomarker can select for patients who would benefit from adjuvant chemotherapy and whether alternatives to platinum plus 5-fluorouracil (PF) are superior in this setting. Whether survival outcome is improved by integration of effective systemic chemotherapy upfront in an era where radiation therapy yields extremely high locoregional control rates has also been the topic of several prospective clinical trials, which have demonstrated definitive benefit to an induction strategy [5, 6]. At the time of the launch of this study, it was unclear whether induction chemotherapy could be administered reliably and feasibly to this patient population using a standard TPF regimen that is widely used in non-NPC squamous cell carcinoma of the head and neck (SCCHN) [7, 8]. We therefore asked whether sequential chemoradiotherapy using induction TPF standard dosing followed by concurrent weekly cisplatin with radiation was feasible and associated with a high progression free survival (PFS).

Tumor hypoxia is a marker for poor prognosis and predicts for decreased radiation response in advanced SCCHN [9]. Hypoxic head and neck cancers are known to be less sensitive to radiation [10]. It is unknown if reversal of hypoxia prior to radiation results in better tumor control with radiation. Data In other cancer settings suggest that both chemotherapy and bevacizumab might reduce tumor hypoxia [11–16]. Therefore, we also explored whether fluoromisonidasole (^18^F-MISO) PET CT scanning could be used to demonstrate hypoxia at baseline and associated reduction with the addition of bevacizumab to the induction TPF (TPFB).

## Materials and methods

This phase II IRB-approved study prospectively enrolled non-metastatic AJCC stage II-IVb (M0) patients with histologically or cytologically confirmed nasopharyngeal carcinoma and an additional, exploratory oligometastatic (M1) patient cohort [17]. Informed consent was obtained prior to study enrollment. Eligibility was restricted to therapy naive patients with an ECOG performance status of <2 with RECIST measurable disease. Normal liver, renal and bone marrow function was required. Patients with peripheral neuropathy or hearing loss thought to render then clinically inappropriate for cisplatin or carboplatin were excluded.

### Exploration of bevacizumab and ^18^F-MISO imaging

Bevacizumab, 15mg/kg, was administered one week prior to C1D1 of induction TPF (TPFB), on D1 in the subsequent two cycles of induction TPF, and at Weeks 1, 4 and 7 during chemoradiation. The amount of ^18^F-MISO injected was 0.1 mCi/kg, but not to exceed 10 mCi. PET CT of the head and upper torso was acquired 90 to 120 minutes post ^18^F-MISO injection. Correlative ^18^F-MISO PET CT imaging occurred at baseline, 5 to 8 days post-dose of first bevacizumab administration, and 2 to 4 weeks after the last TPFB dose, prior to initiation of radiation treatment. Hypoxia was defined as a tissue to blood ^18^F-MISO detection ratio of 1.2 or greater [18, 19]. Tumor regions of interest were established using the ^18^F-MISO PET data fused with CT data for attenuation correction. A functional hypoxic volume of all RECIST evaluable tumor regions (primary site and lymph nodes) was calculated, based on the number of voxels with a tumor to blood ratio of 1.2 or greater and using MIMVista 6.1 software. The relative hypoxic volumes at baseline, after bevacizumab, and after TPFB were compared both quantitatively and qualitatively using descriptive statistics.

### Treatment regimen

The TPF treatment was three, 3-week cycles of docetaxel 75mg/m^2^ and cisplatin 75mg/m^2^ (or carboplatin AUC 6 for patients not cisplatin eligible) on day 1, and 750 mg/m^2^ 5-FU on days 1 through 5. Prophylactic ciprofloxacin and pegfilgrastim were administered per institutional standard with the TPF regimen.

Chemoradiation following TPF was initiated in the protocol-specified time range of 3–6 weeks from cycle 3 day 1 of TPF. Chemoradiation consisted of cisplatin 40 mg/m^2^ or carboplatin AUC 1.5 weekly x 6, concurrent with intensity modulated radiation therapy (IMRT) to 70 Gy to the gross target volume + margins delivered in 33 fractions over 6.5–7 weeks.

Dose reductions of docetaxel from 75 to 60 mg/m^2^, cisplatin from 75 to 60 mg/m^2^ and 5- FU from 750 mg/m^2^ to 600 mg/m^2^ to 480mg/m^2^ during induction were mandated for dose limiting toxicities. During chemoradiation, cisplatin reductions from 40 mg/m^2^ to 30 mg/m^2^ to 25 mg/m^2^, were specified for drug attributable severe adverse events (AEs). Carboplatin was substituted for cisplatin for Grade 2 and higher neuropathy, hearing loss or renal dysfunction.

### Response and adverse event criteria

All AEs Grade 2 and above were evaluated at screening, every 3 weeks from first dose of TPF, at the end of induction, every week during chemoradiation, and at the first follow up 1 month after the last dose of study treatment. Toxicities were graded according to the Common Terminology Criteria for Adverse Events Version 4.0 [20].

Tumor response and progression were evaluated using the Response Evaluation Criteria in Solid Tumors (RECIST) guideline version 1.1 [21]. Patients were assessed for response post-induction, and at 1 to 3 months, 12 months and 24 months post-radiation.

### Statistical considerations

Progression Free Survival (PFS)rate with PFSdefined as the interval between registration and cancer progression or death at 2 years was the primary endpoint. Patients who received at least one cycle of TPF and had their disease re-evaluated were considered evaluable for response. The initially planned sample size is 40 evaluable patients in total, which could provide at least 87% power to distinguish ≥15% AE rates from an acceptable AE rate of 3%, under a fixed type I error rate of 5%. Accrual was stopped after 20 patients for the M0 patients because of publication of a larger phase 3 trial [5], which demonstrated benefit for modified TPF in this setting, rendering further accrual not feasible. Twenty M0 patients can estimate the null AE rate of 3% with 95% CI (0.1, 24.9)% in a single-arm design. In the M1 exploratory cohort, six patients can estimate the null AE rate of 3% with 95% CI (0.4, 64.1)%.

Secondary endpoints included overall survival (OS), estimated according to the methods of Kaplan and Meier, and rates of adverse events. In the 20 M0 patients, there was 0.75 power to distinguish an AE rate of 3% from an AE rate of 15% when using the significance level of 0.25. In the exploratory cohort of M1 patients, there was 0.52 power to distinguish an AE rate of 3% from an AE rate of 15% when using the same significance level.

Because it was not feasible with the accrual rate planned to introduce an early stopping rule for a PFS at 2 years, this study included an early stopping rule based on RECIST determined CR rates. If there were less than 10 CRs in the first 18 patients, the trial would be stopped. If the trial proceeded to the second stage, the treatment would be considered worthy of further study if 33 or more of the 40 patients are progression-free at 2 years.

All statistical analyses were performing using R version 3.6.3 (2020-02-29), including additional packages "pwr”, “survival”, and “survminer”.

## Results

### Patient characteristics

**Fig 1:**
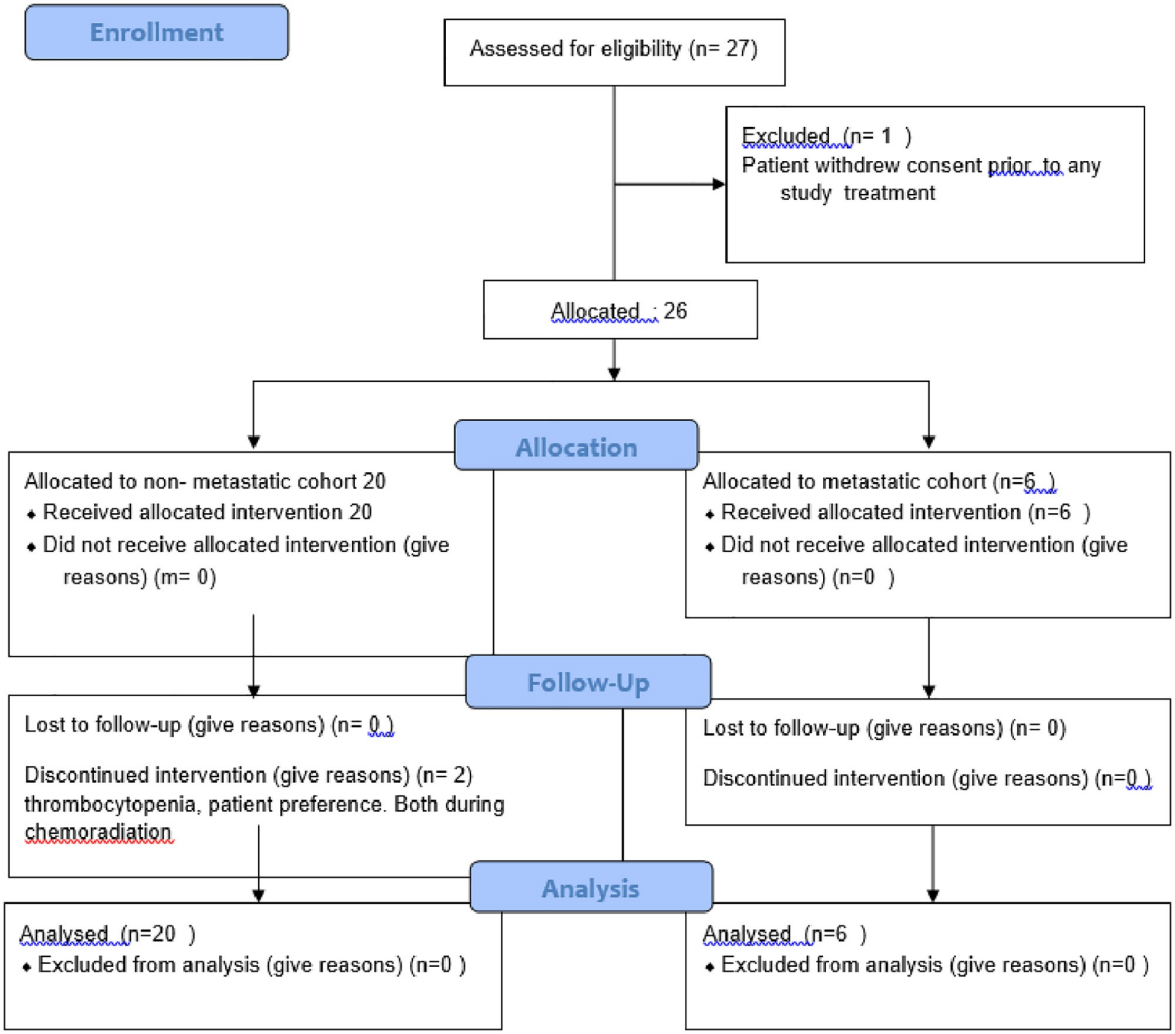
Consort diagram for trial enrollment.

Between June 2009 and August 2017, 27 patients were enrolled on the study. All recruitments, treatments and evaluations took place at the Stanford University Medical Center. See consort diagram in Fig 1. One patient withdrew full consent prior to receiving any study treatment. The 26 remaining patients received at least one cycle of TPF, and were thus evaluable for response per protocol. Twenty of the 26 patients were enrolled in the main M0 cohort, and six patients enrolled in the M1 cohort. See Table 1. The first five patients enrolled also participated in the bevacizumab and ^18^F-MISO imaging portion of the study.

**Table 1:** Patient demographics.

**Sex**	
** Men**	19 (73%)
** Female**	7 (27%)
**Age, years**	51 (41–55)
**T Category**	
** T1**	4 (15%)
** T2**	4 (15%)
** T3**	7 (27%)
** T4**	11 (42%)
**N Category**	
** N0**	1 (4%)
** N1**	7 (27%)
** N2**	14 (54%)
** N3**	4 (15%)
**M Category**	
** M0**	20 (77%)
** M1**	6 (23%)
**Disease Stage** ^1^	
** IIA**	1 (4%)
** IIB**	3 (12%)
** III**	8 (31%)
** IVA**	6 (23%)
** IVB**	2 (7%)
** IVC**	6 (23%)

Data are in n (%) or median (IQR).

^1^ American Joint Committee of Cancer 6^th^ Ed. [17].

### Bevacizumab and ^18^F-MISO imaging

**Fig 2:**
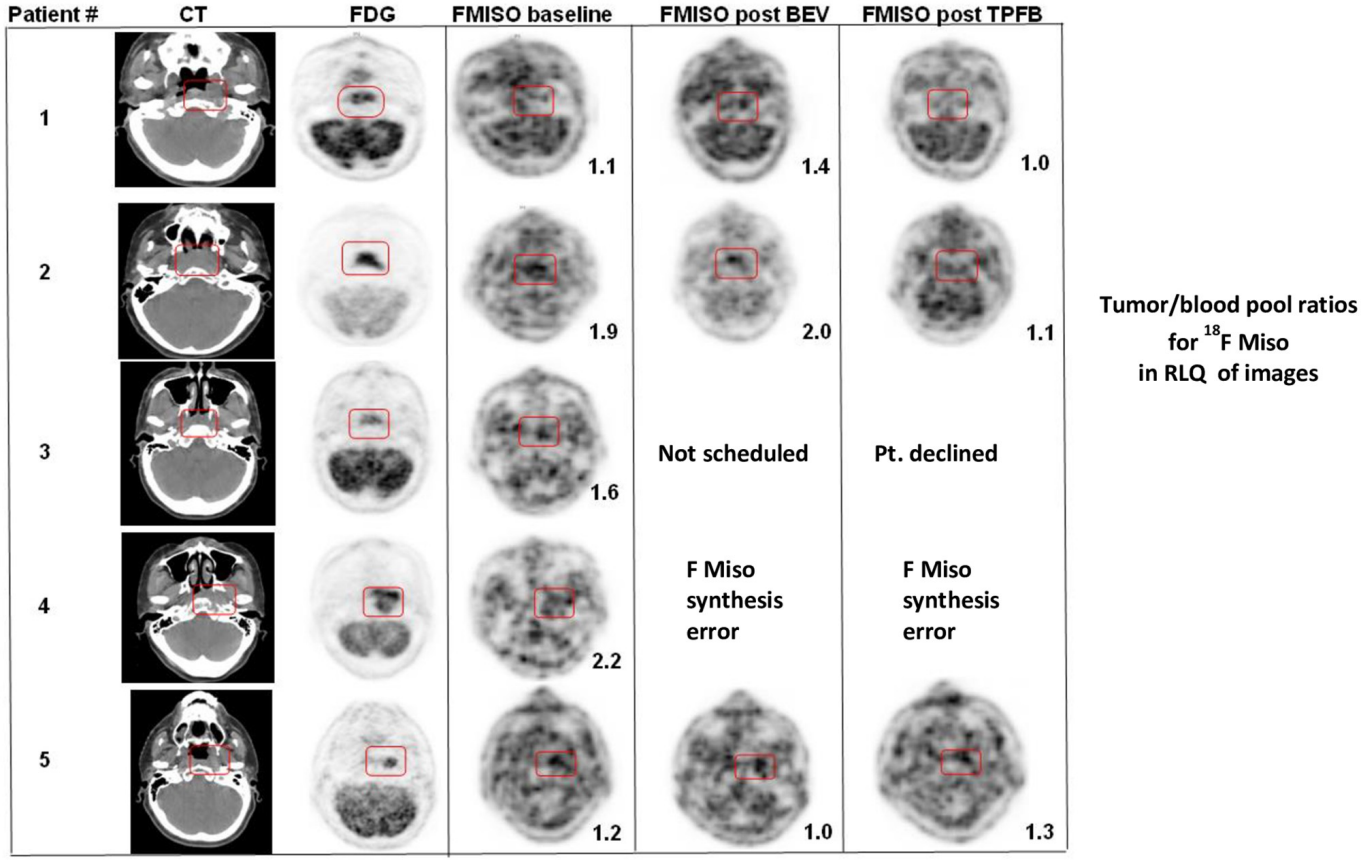
^18^F-MISO correlative imaging results.

The baseline hypoxia detection with the ^18^F-MISO failed to reveal substantial hypoxia and the signal to noise ratio was greater than expected, with only three of five patients with hypoxia as defined in the methods section. See Fig 2. Additionally, acquisition of the ^18^F-MISO tracer from a remote contractor proved to be not feasible. On more than one occasion the reagent could not be synthesized, transported, and delivered to the patient in a timely manner. Therefore, the protocol was amended to remove bevacizumab and ^18^F-MISO imaging.

### Progression Free Survival (PFS)

**Fig 3:**
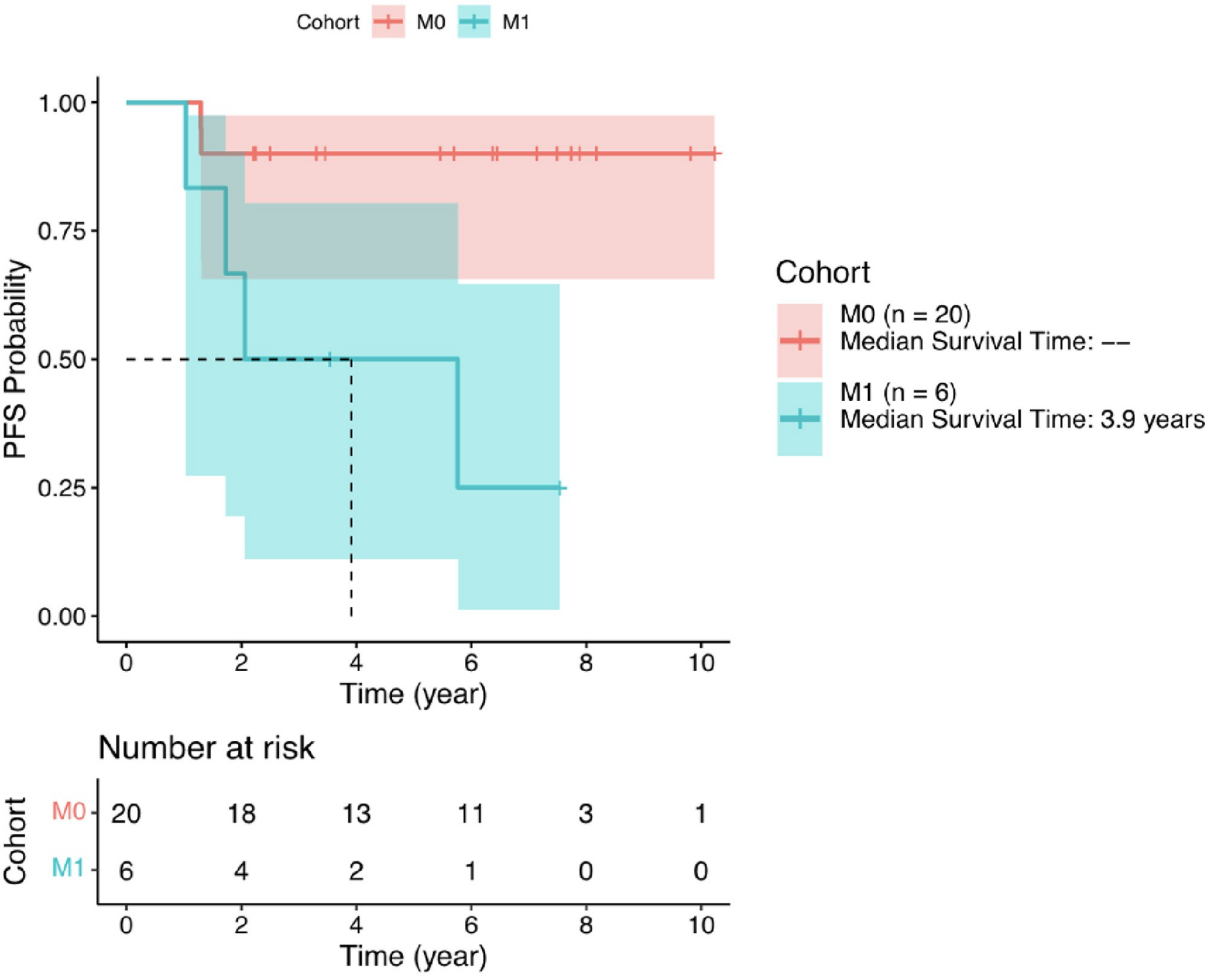
Kaplan-Meier progression free survival curve for M0 cohort and M1 cohort.

The PFS rate at 2 years in the M0 cohort was 90% (95% CI: 65.6%–97.4%) and was preserved through 5 years; the median was not reached. The PFS rate at 2 years in the M1 cohort was 66.7% (95% CI: 19.5%–90.4%) with the median of 3.9 years of follow up. See Fig 3.

### Overall Survival (OS)

**Fig 4:**
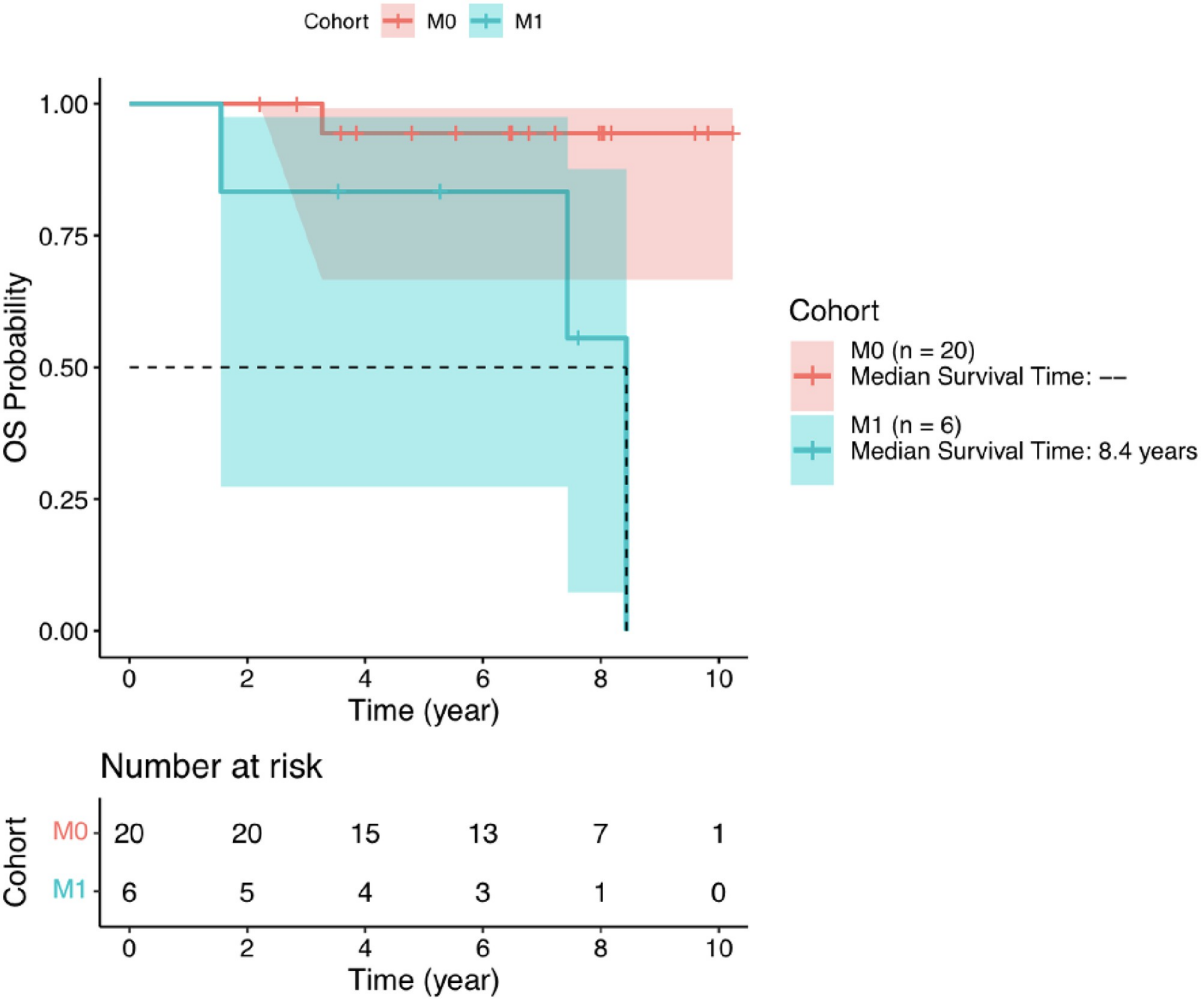
Kaplan-Meier overall survival curve for M0 cohort and M1 cohort.

The OS rate at 2 years in the Cohort M0 was 100%, with the median survival time not reached. The one death was from disease progression. Further, rate the OS in the M0 cohort at 5 years was 94.4%. The OS rate at 2 years in the M1 cohort was 83.3% (95% CI: 27.3%–97.5%), with three deaths from disease progression. See Fig 4.

### Best response

Twenty patients in the M0 cohort were evaluated for best response to TPF and best overall response. See Table 2. After TPF induction, 16 of 20 patients achieved partial response. Three patients had stable disease, and one patient had an indeterminate response. Following concurrent chemoradiation, 16 patients achieved complete response and two patients achieved partial response. The remaining two patients were not evaluable for final response due to premature withdrawal from protocol specified chemoradiation. One patient discontinued concurrent chemoradiation treatment due to thrombocytopenia and was switched to off-protocol chemoradiation with cetuximab. That patient achieved complete response by PET CT imaging. The other patient withdrew from treatment protocol during concurrent chemoradiation treatment for nonmedical reasons, later restarting treatment off protocol after radiographical progression, subsequently achieving near complete response by PET CT imaging.

**Table 2:** Best response to TPF and best overall response in M0 and M1 patients.

	M0 Cohort		M1 Cohort	
	Best Response to TPF	Best Overall Response	Best Response to TPF	Best Overall Response
Stable Disease	3	-	1	-
Partial Response	16^a^	2	5^b^	3
Complete Response	-	16	-	3
Indeterminate/Not Evaluable	1	2	-	-

^a^ 3 of 16 patients response confirmed by nonstandard RECIST.

^b^ 1 of 5 patients response confirmed by nonstandard RECIST.

Six patients in the exploratory M1 cohort were evaluated for best overall response. See Table 2. After TPF induction, five patients achieved partial response. One of the five patients achieved partial response that could not be confirmed by RECIST criteria as follow up imaging did not capture all target lesions. One patient had stable disease. Following concurrent chemoradiation, three patients achieved complete response and three patients achieved partial response.

### Assessment of treatment feasibility

#### Induction chemotherapy

All M0 patients (n = 20) completed TPF induction chemotherapy; eighteen patients received full intended dose. One patient received 87% of intended docetaxel dose because of neutropenia. One patient received 93% of intended docetaxel, cisplatin and 5-FU due to diarrhea, mucositis and neuropathy.

All M1 patients (n = 6) completed TPF induction chemotherapy; five of the M1 patients received full-intended dose. The remaining patient received 87% of intended cisplatin dose due to nausea and vomiting.

#### Concurrent chemoradiation

M0 patients began concurrent chemoradiation a median of 31 days (21–38 d) after cycle 3 day 1 induction TPF, and median radiation time was 44 days (8–49 d). Nineteen of the 20 patients received 70 Gy. The one patient receiving less than intended dose withdrew from the protocol treatment after 7 fractions of radiation (14.84 Gy) as mentioned above for nonmedical reasons. One year later, the patient restarted induction chemotherapy and concurrent chemoradiation, consisting of 4 doses of carboplatin and paclitaxel and an additional 66 Gy of radiation.

Eighteen M0 patients were started on concurrent cisplatin, with the remaining two patients starting on carboplatin due to baseline hearing loss. The median percentage of intended concurrent platinum-based chemotherapy received was 83% (33%–117%). Seven patients received full-intended concurrent chemotherapy dose and 17 patients received at least 60% of the full-intended concurrent platinum dose. See Appendix A in S1 Appendix. Two patients received 33% of intended dose due to premature withdrawal from study treatment. One of the two patients was the same, aforementioned patient who withdrew from protocol treatment. The other patient who received 33% of intended dose was discontinued from concurrent platinum-based chemotherapy due to thrombocytopenia and was switched to cetuximab with concurrent radiation off study, completing 4 doses of cetuximab during radiation. One patient received 58% of intended dose due to thrombocytopenia. One patient received 63% of intended dose due to neutropenia and thrombocytopenia. Two patients received 67% due to thrombocytopenia and neutropenia. One patient received 79% of intended dose due to neutropenia. Four patients received 83% of the intended dose due to neutropenia, thrombocytopenia, neuropathy, and hearing impairment. Two patients received an additional non-protocol specified 7^th^ dose of cisplatin in their final week of radiation.

The M1 patients (n = 6) began chemoradiation a median of 33 days (22–39 d) after cycle 3 day 1 of induction TPF and median radiation time was 45 days (42–48 d). Five of the six patients received 70 Gy and one patient received 60 Gy, per treating radiation oncologist decision. Three patients received full-intended cisplatin dose and all patients received at least 60% of full-intended cumulative platinum dose. One patient received 67% of intended cisplatin dose due to thrombocytopenia. One patient received 83% of intended dose due to thrombocytopenia. One patient received 96% of intended dose due to neutropenia. One of the six patients switched to carboplatin due to neuropathy, fatigue and anorexia, subsequently completing concurrent chemotherapy at the full-intended dose. See Appendix B in S1 Appendix.

#### Adverse events

One M0 patient discontinued protocol treatment due to thrombocytopenia after the second concurrent cisplatin dose. None of the M1 patients were discontinued from protocol treatment for AEs. See Tables 3 and 4.

**Table 3:** Adverse events of M0 cohort, worst grade per patient for each event.

	Induction Chemotherapy (n = 20)			Chemoradiation (n = 20)		
	Grade 2	Grade 3	Grade 4	Grade 2	Grade 3	Grade 4
**Hematological**						
Anemia	4 (20%)	1 (5%)	-	6 (30%)	2 (10%)	-
Febrile Neutropenia	-	3 (15%)	-	-	-	-
Lymphocyte Count Decreased	5 (25%)	3 (15%)	-	1 (5%)	6 (30%)	7 (35%)
Neutropenia	1 (5%)	1 (5%)	4 (20%)	6 (30%)	1 (5%)	1 (5%)
Thrombocytopenia	2 (10%)	-	-	4 (20%)	1 (5%)	-
White Blood Cell Decreased	1 (5%)	2 (10%)	-	4 (20%)	2 (10%)	-
**Non-Hematological**						
Abdominal Distension	1 (5%)	-	-	-	-	-
Abdominal Pain	1 (5%)	-	-	-	-	-
Acid Reflux	-	-	-	1 (5%)	-	-
Alanine Aminotransferase Increase	1 (5%)	-	-	-	-	-
Alopecia	2 (10%)	-	-	1 (5%)	-	-
Anorexia	1 (5%)	-	-	1 (5%)	-	-
Constipation	-	-	-	1 (5%)	-	-
Coronavirus	-	1 (5%)	-	-	-	-
Dehydration	2 (10%)	-	-	1 (5%)	-	-
Dermatitis	1 (5%)	-	-	9 (45%)	-	-
Diarrhea	2 (10%)	1 (5%)	-	-	-	-
Dysgeusia	4 (20%)	-	-	4 (20%)	-	-
Dysphagia	1 (5%)	-	-	3 (15%)	1 (5%)	-
Fatigue	6 (30%)	-	-	4 (20%)	-	-
Hyperglycemia	2 (10%)	1 (5%)	-	1 (5%)	-	-
Hypoalbuminemia	3 (15%)	-	-	1 (5%)	-	-
Hypocalcemia	-	-	-	1 (5%)	-	-
Hypokalemia	-	-	1 (5%)	-	-	-
Muscle Weakness	1 (5%)	-	-	-	-	-
Nausea	2 (10%)	-	-	4 (20%)	1 (5%)	-
Neuropathy	1 (5%)	-	-	-	1 (5%)	-
Oral Mucositis	4 (20%)	1 (5%)	-	7 (35%)	10 (50%)	-
Oral Pain	-	-	-	3 (15%)	1 (5%)	-
Pain in Extremities	-	-	-	1 (5%)	-	-
Palmar Plantar Syndrome	-	1 (5%)	-	-	-	-
Paresthesia	1 (5%)	-	-	1 (5%)	-	-
Salivary Duct Inflammation	-	-	-	1 (5%)	1 (5%)	-
Skin Infection	1 (5%)	-	-	-	-	-
Somnolence	-	1 (5%)	-	-	-	-
Thrush	1 (5%)	-	-	-	-	-
Typhlitis	-	1 (5%)	-	-	-	-
Vomiting	2 (10%)	-	-	-	-	-
Weight Loss	2 (10%)	-	-	4 (20%)	-	-
Xerostomia	1 (5%)	-	-	4 (20%)	-	-
**Worst AE Grade per Patient**	9 (45%)	6 (30%)	5 (25%)	1 (5%)	11 (55%)	8 (40%)

**Table 4:** Adverse events of M1 cohort, worst grade per patient for each event.

	Induction Chemotherapy (n = 6)			Chemoradiation (n = 6)		
	Grade 2	Grade 3	Grade 4	Grade 2	Grade 3	Grade 4
**Hematological**						
Anemia	2 (33%)	-	-	1 (17%)	1 (17%)	-
Febrile Neutropenia	-	2 (33%)	-	-	-	-
Lymphocyte count decreased	-	2 (33%)	-	1 (17%)	1 (17%)	2 (33%)
Neutropenia	-	2 (33%)	-	1 (17%)	2 (33%)	
Thrombocytopenia	-	-	-	1 (17%)	-	-
**Non-Hematological**						
Alopecia	2 (33%)	-	-	-	-	-
Anorexia	1 (17%)	-	-	1 (17%)	-	-
Creatinine Increased	-	-	-	1 (17%)	-	-
Colitis	1 (17%)	-	-	-	-	-
Dehydration	-	1 (17%)	-	-	-	-
Dermatitis	-	-	-	5 (83%)	-	1 (17%)
Diarrhea	1 (17%)	-	-	-	-	-
Dysgeusia	-	-	-	1 (17%)	-	-
Dysphagia	1 (17%)	-	-	1 (17%)	-	-
Fatigue	2 (33%)	-	-	1 (17%)	1 (17%)	-
Hyperglycemia	1 (17%)	-	-	-	1 (17%)	-
Hypertension	-	-	-	-	1 (17%)	-
Hypoalbuminemia	1 (17%)	1 (17%)	-	1 (17%)	-	-
Hypocalcemia	1 (17%)	-	1 (17%)	1 (17%)	-	-
Hypokalemia	1 (17%)	-	-	1 (17%)	-	-
Hyponatremia	-	-	1 (17%)	-	-	-
Laryngeal Inflammation	1 (17%)	-	-	-	-	-
Nausea	-	1 (17%)	-	2 (33%)	-	-
Neuropathy	-	-	-	1 (17%)	-	-
Oral Mucositis	2 (33%)	1 (17%)	-	3 (50%)	3 (50%)	-
Otitis Media	-	-	-	1 (17%)	-	-
Pulmonary Embolism	-	1 (17%)	-	-	-	-
Salivary duct inflammation	-	-	-	1 (17%)	-	-
Skin Problem	-	-	-	-	1 (17%)	-
Urinary Tract Infection	-	-	-	1 (17%)	-	-
Vomiting	-	1 (17%)	-	-	-	-
Weight Loss	-	-	-	2 (33%)	-	-
**Worst AE Grade per Patient**	-	5 (83%)	1 (17%)	-	3 (50%)	3 (50%)

## Discussion

We have demonstrated in this exploratory study that the administration of standard dose TPF as induction chemotherapy in patients with locoregionally advanced NPC is both feasible and effective when coupled with definitive concurrent chemoradiation. While the number of patients in this study is small, this approach is associated with excellent long term OS and PFS. Whether standard TPF induction dosing is superior to either dose attenuated TPF as used in the Chinese phase 3 TPC study or gemcitabine and carboplatin (GC) induction chemotherapy remains an open question [5, 6]. It is worth noting that there are no data supporting the use of dose attenuated TPF selected by Chinese investigators outside of their study. The assumption that conventional dosing of TPF in appropriately selected patients is not feasible in NPC patients is refuted by our data. There are limited data from a Groupe Oncologie Radiotherapie Tete et Cou (GORTEC) based study of TPF [22] induction in NPC, using an identical dose regimen as ours. While the GORTEC study was closed prematurely for low accrual, they observed, as we have, that TPF did not compromise tolerance to the chemoradiation. In their study, TPF was associated with a numerically superior survival difference versus concurrent chemoradiation alone (hazard ratio (HR) for death 0.4), a greater difference than seen in the dose attenuated TPF study (HR for death 0.59). Because the induction trial with GC was associated with a HR for death of 0.43, it is unclear whether further study of TPF is warranted in this situation [5]. However, one meta-analysis has concluded that TPF-based induction chemotherapy results in better survival outcomes [23] compared to double drug based induction chemotherapy, suggesting that a definitive trial designed to ask whether conventional dose TPF is superior could be contemplated. Therefore, this study supports the use of TPF as induction for patients with locoregionally advanced NPC, but further trials will be needed to optimize the TPF regimen and to ask whether TPF or other regimens such as gemcitabine plus cisplatin, or newer regimens, are optimal.

With regards to the exploratory objective of using bevacizumab and ^18^F-MISO PET CT imaging, the lack of substantial hypoxia levels at baseline in the initial patents led us to conclude that ^18^F-MISO PET CT imaging does not warrant development as a hypoxia evaluation tool.

## Supporting information

S1 File Supplemental patient specific activity data per PLOS ONE request. (PDF)

S2 File Patient AE data. (PDF)

S3 File TPF protocol original IRB approved. (PDF)

S1 Checklist TREND statement checklist. (PDF)

S1 Appendix (PDF)
