# Supplementary material for: In search for optimal induction chemotherapy for advanced nasopharyngeal cancer: Standard dosing of Docetaxel, Platinum, and 5-Fluorouracil (TPF) followed by chemoradiation
Source: PLoS One. 2023 Feb 2;18(2):e0276651. doi: 10.1371/journal.pone.0276651 (PMC9894485; doi:10.1371/journal.pone.0276651)
Supplement: S1 File — (PDF) [file pone.0276651.s001.pdf]

Supplemental patient specific activity data per PLOS ONE request

Baseline demographics

Record ID	Event Name	TNM Status	sex	enrollment date
TPF01	Baseline/Pre-treatment	T3N2M1	Male	6/8/2009
**TPF02**	**Baseline/Pre-treatment**	**T3N2M0**	**Female**	**9/25/2009**
TPF03	Baseline/Pre-treatment	T3N2M0	Male	1/5/2010
**TPF04**	**Baseline/Pre-treatment**	**T3N2M1**	**Female**	**2/19/2010**
TPF05	Baseline/Pre-treatment	T2N0M0	Male	3/16/2010
**TPF09**	**Baseline/Pre-treatment**	**T2N1M0**	**Female**	**9/20/2011**
TPF10	Baseline/Pre-treatment	T4N1M0	Male	10/4/2011
**TPF11**	**Baseline/Pre-treatment**	**T3N1M0**	**Female**	**11/29/2011**
TPF12	Baseline/Pre-treatment	T3N2M0	Male	12/16/2011
**TPF13**	**Baseline/Pre-treatment**	**T4N2M0**	**Female**	**1/31/2012**
TPF14	Baseline/Pre-treatment	T4N3M1	Male	4/26/2012
TPF15	Baseline/Pre-treatment	T4N3M1	Male	5/17/2012
TPF16	Baseline/Pre-treatment	T1N2M0	Male	12/14/2012
TPF17	Baseline/Pre-treatment	T2N2M0	Male	3/1/2013
**TPF19**	**Baseline/Pre-treatment**	**T4N1M0**	**Female**	**5/29/2013**
TPF20	Baseline/Pre-treatment	T1N2M0	Male	6/21/2013
TPF21	Baseline/Pre-treatment	T4N1M0	Male	7/9/2013
TPF22	Baseline/Pre-treatment	T1N1M0	Male	10/3/2013
TPF23	Baseline/Pre-treatment	T4N2M1	Male	8/26/2014
**TPF24**	**Baseline/Pre-treatment**	**T4N3M0**	**Female**	**9/18/2014**
TPF25	Baseline/Pre-treatment	T2N3M0	Male	1/21/2015
TPF26	Baseline/Pre-treatment	T4N2M0	Male	1/14/2016
TPF27	Baseline/Pre-treatment	T4N2cM0	Male	5/12/2016
TPF28	Baseline/Pre-treatment	T4N2M1	Male	6/3/2016
TPF29	Baseline/Pre-treatment	T3N1-2M0	Male	2/2/2017
TPF30	Baseline/Pre-treatment	T1N1M0	Male	8/28/2017

Response data, M0 group

Record ID	Event Name	Date of Radiological Measurement	Targeted lesions response evaluation-RECIST(Note: Leave blank for baseline visit)	Overall response(Note: Leave blank for baseline visit)	Best Response
TPF02	2 Tumor Response 3mo Post Rdx	4/15/2010	CR (Complete Response) - All lesions gone and tumor markers normal	CR - Complete Response	
TPF02	3 Tumor Response 12mo Post Rdx	2/25/2011	CR (Complete Response) - All lesions gone and tumor markers normal	CR - Complete Response	
TPF02	4 Tumor Response 24mo Post Rdx	1/15/2012	CR (Complete Response) - All lesions gone and tumor markers normal	CR - Complete Response	
TPF02	Follow Up				CR - per RECIST
TPF03	2 Tumor Response 3mo Post Rdx	8/12/2010	CR (Complete Response) - All lesions gone and tumor markers normal	CR - Complete Response	
TPF03	3 Tumor Response 12mo Post Rdx	5/24/2011	CR (Complete Response) - All lesions gone and tumor markers normal	CR - Complete Response	

TPF03	4 Tumor Response 24mo Post Rdx	7/23/2012	CR (Complete Response) - All lesions gone and tumor markers normal	CR - Complete Response	
TPF03	Follow Up				CR - per RECIST
TPF05	2 Tumor Response 3mo Post Rdx	10/26/2010	CR (Complete Response) - All lesions gone and tumor markers normal	CR - Complete Response	
TPF05	3 Tumor Response 12mo Post Rdx	7/26/2011	CR (Complete Response) - All lesions gone and tumor markers normal	CR - Complete Response	
TPF05	4 Tumor Response 24mo Post Rdx	6/28/2012	CR (Complete Response) - All lesions gone and tumor markers normal	CR - Complete Response	
TPF05	Follow Up				CR - per RECIST
TPF09	2 Tumor Response 3mo Post Rdx	3/11/2012	CR (Complete Response) - All lesions gone and tumor markers normal	CR - Complete Response	
TPF09	3 Tumor Response 12mo Post Rdx	1/16/2013	CR (Complete Response) - All lesions gone and tumor markers normal	CR - Complete Response	
TPF09	4 Tumor Response 24mo Post Rdx	2/27/2014	CR (Complete Response) - All lesions gone and tumor markers normal	CR - Complete Response	

TPF09	Follow Up				CR - per RECIST
TPF10	2 Tumor Response 3mo Post Rdx	3/30/2012	CR (Complete Response) - All lesions gone and tumor markers normal	CR - Complete Response	
TPF10	3 Tumor Response 12mo Post Rdx	2/8/2014	CR (Complete Response) - All lesions gone and tumor markers normal	CR - Complete Response	
TPF10	4 Tumor Response 24mo Post Rdx	3/2/2015	CR (Complete Response) - All lesions gone and tumor markers normal	CR - Complete Response	
TPF10	Follow Up				CR - per RECIST
TPF11	2 Tumor Response 3mo Post Rdx	5/25/2012	CR (Complete Response) - All lesions gone and tumor markers normal	CR - Complete Response	
TPF11	3 Tumor Response 12mo Post Rdx	2/10/2014	CR (Complete Response) - All lesions gone and tumor markers normal	CR - Complete Response	
TPF11	4 Tumor Response 24mo Post Rdx	9/4/2015	CR (Complete Response) - All lesions gone and tumor markers normal	CR - Complete Response	
TPF11	Follow Up				CR - per RECIST

TPF12	2 Tumor Response 3mo Post Rdx	6/27/2012	PR (Partial Response)- Current sum >30% decrease from baseline sum	PR - Partial Response	
TPF12	3 Tumor Response 12mo Post Rdx	5/23/2013	CR (Complete Response) - All lesions gone and tumor markers normal	CR - Complete Response	
TPF12	4 Tumor Response 24mo Post Rdx	7/11/2015	CR (Complete Response) - All lesions gone and tumor markers normal	CR - Complete Response	
TPF12	Follow Up				CR - per RECIST
TPF13	2 Tumor Response 3mo Post Rdx	4/25/2012		IR - Indeterminate Response	
TPF13	3 Tumor Response 12mo Post Rdx	3/20/2013	PD (Progressive Disease)- Current Sum >20% increase from the best response	PD - Progressive Disease	
TPF13	4 Tumor Response 24mo Post Rdx	2/26/2014		IR - Indeterminate Response	

TPF13	Follow Up				achieved partial response status post TPF induction, stopped chemoradiation prematurely, was lost to follow up but returned to Stanford to discuss treatment discussions after radiological disease progression and restarted treatment off protocol. Achieved CR re-induction with TPF x1 then TP x2 then carboplatin and paclitaxel with radiation (66 Gy) which she completed on 9/6/2013 on off treatment protocol
TPF16	2 Tumor Response 3mo Post Rdx	6/23/2013	PR (Partial Response)- Current sum >30% decrease from baseline sum	PR - Partial Response	
TPF16	3 Tumor Response 12mo Post Rdx	3/31/2014	CR (Complete Response) - All lesions gone and tumor markers normal	CR - Complete Response	

TPF16	4 Tumor Response 24mo Post Rdx	2/23/2015	CR (Complete Response) - All lesions gone and tumor markers normal	CR - Complete Response	
TPF16	Follow Up				CR - per RECIST
TPF17	2 Tumor Response 3mo Post Rdx	8/30/2013	PR (Partial Response)- Current sum >30% decrease from baseline sum	PR - Partial Response	
TPF17	3 Tumor Response 12mo Post Rdx	3/8/2014	CR (Complete Response) - All lesions gone and tumor markers normal	CR - Complete Response	
TPF17	4 Tumor Response 24mo Post Rdx	9/21/2015	CR (Complete Response) - All lesions gone and tumor markers normal	CR - Complete Response	
TPF17	Follow Up				CR - per RECIST
TPF19	2 Tumor Response 3mo Post Rdx	11/14/2013	CR (Complete Response) - All lesions gone and tumor markers normal	CR - Complete Response	
TPF19	3 Tumor Response 12mo Post Rdx	11/17/2014	CR (Complete Response) - All lesions gone and tumor markers normal	CR - Complete Response	
TPF19	4 Tumor Response 24mo Post Rdx	5/12/2016	CR (Complete Response) - All lesions gone and tumor markers normal	CR - Complete Response	

TPF19	Follow Up				CR - per RECIST
TPF20	2 Tumor Response 3mo Post Rdx	12/23/2013	CR (Complete Response) - All lesions gone and tumor markers normal	CR - Complete Response	
TPF20	3 Tumor Response 12mo Post Rdx	12/19/2014		CR - Complete Response	
TPF20	4 Tumor Response 24mo Post Rdx	12/18/2015	CR (Complete Response) - All lesions gone and tumor markers normal	CR - Complete Response	
TPF20	Follow Up				CR - per RECIST
TPF21	2 Tumor Response 3mo Post Rdx	2/5/2014	CR (Complete Response) - All lesions gone and tumor markers normal	CR - Complete Response	
TPF21	3 Tumor Response 12mo Post Rdx	12/13/2014	CR (Complete Response) - All lesions gone and tumor markers normal	CR - Complete Response	
TPF21	4 Tumor Response 24mo Post Rdx	1/15/2015	CR (Complete Response) - All lesions gone and tumor markers normal	IR - Indeterminate Response	
TPF21	Follow Up				CR - clinical and after switching to off treatment protocol

TPF22	2 Tumor Response 3mo Post Rdx	5/9/2014	CR (Complete Response) - All lesions gone and tumor markers normal	CR - Complete Response	
TPF22	3 Tumor Response 12mo Post Rdx	3/10/2015	CR (Complete Response) - All lesions gone and tumor markers normal	CR - Complete Response	
TPF22	4 Tumor Response 24mo Post Rdx	4/5/2016	CR (Complete Response) - All lesions gone and tumor markers normal	CR - Complete Response	
TPF22	Follow Up				CR - per RECIST
TPF24	2 Tumor Response 3mo Post Rdx	3/11/2015	PR (Partial Response)- Current sum >30% decrease from baseline sum	PR - Partial Response	
TPF24	3 Tumor Response 12mo Post Rdx	11/11/2015	CR (Complete Response) - All lesions gone and tumor markers normal	CR - Complete Response	
TPF24	4 Tumor Response 24mo Post Rdx	12/8/2016	CR (Complete Response) - All lesions gone and tumor markers normal	CR - Complete Response	
TPF24	Follow Up				CR - per RECIST
TPF25	2 Tumor Response 3mo Post Rdx	7/2/2015	PR (Partial Response)- Current sum >30% decrease from baseline sum	PR - Partial Response	

TPF25	3 Tumor Response 12mo Post Rdx	8/1/2016	PR (Partial Response)- Current sum >30% decrease from baseline sum	PR - Partial Response	
TPF25	4 Tumor Response 24mo Post Rdx	12/2/2016	PD (Progressive Disease)- Current Sum >20% increase from the best response	PD - Progressive Disease	
TPF25	Follow Up				PR - per RECIST
TPF26	2 Tumor Response 3mo Post Rdx	8/15/2016	PR (Partial Response)- Current sum >30% decrease from baseline sum	PR - Partial Response	
TPF26	3 Tumor Response 12mo Post Rdx	5/11/2017	PR (Partial Response)- Current sum >30% decrease from baseline sum	PR - Partial Response	
TPF26	4 Tumor Response 24mo Post Rdx	6/6/2018	PR (Partial Response)- Current sum >30% decrease from baseline sum	PR - Partial Response	
TPF26	Follow Up				PR - per RECIST
TPF27	2 Tumor Response 3mo Post Rdx	12/9/2016	PR (Partial Response)- Current sum >30% decrease from baseline sum	PR - Partial Response	

TPF27	3 Tumor Response 12mo Post Rdx	9/1/2017	PR (Partial Response)- Current sum >30% decrease from baseline sum	PR - Partial Response	
TPF27	4 Tumor Response 24mo Post Rdx	9/14/2018	CR (Complete Response) - All lesions gone and tumor markers normal	CR - Complete Response	
TPF27	Follow Up				CR - per RECIST
TPF29	2 Tumor Response 3mo Post Rdx	9/1/2017	PR (Partial Response)- Current sum >30% decrease from baseline sum	PR - Partial Response	
TPF29	3 Tumor Response 12mo Post Rdx	5/15/2018	CR (Complete Response) - All lesions gone and tumor markers normal	CR - Complete Response	
TPF29	4 Tumor Response 24mo Post Rdx	8/1/2019	CR (Complete Response) - All lesions gone and tumor markers normal	CR - Complete Response	
TPF29	Follow Up				CR - per RECIST
TPF30	2 Tumor Response 3mo Post Rdx	4/9/2018	PR (Partial Response)- Current sum >30% decrease from baseline sum	PR - Partial Response	

TPF30	3 Tumor Response 12mo Post Rdx	12/10/2018	CR (Complete Response) - All lesions gone and tumor markers normal	CR - Complete Response	
TPF30	4 Tumor Response 24mo Post Rdx	11/11/2019	CR (Complete Response) - All lesions gone and tumor markers normal	CR - Complete Response	
TPF30	Follow Up				CR - per RECIST

Response data M1 group

Record ID	Event Name	Date of Radiological Measurement	Targeted lesions response evaluation-RECIST(Note: Leave blank for baseline visit)	Non targeted lesions response evaluation-RECIST(Note: Leave blank for baseline visit)	Overall response(Note: Leave blank for baseline visit)	Best Response
TPF01	2 Tumor Response 3mo Post Rdx	1/15/2010	PR (Partial Response)- Current sum >30% decrease from baseline sum		PR - Partial Response	

TPF01	3 Tumor Respons e 12mo Post Rdx	3/1/2011	PD (Progressive Disease)- Current Sum >20% increase from the best response		PD - Progressive Disease	
TPF01	4 Tumor Respons e 24mo Post Rdx					
TPF01	Follow Up					PR - per RECIST
TPF04	2 Tumor Respons e 3mo Post Rdx	9/14/2010	CR (Complete Response) - All lesions gone and tumor markers normal	IR (Indeterminate Response)/SD(Stabl e Disease) - Anything else	PR - Partial Response	
TPF04	3 Tumor Respons e 12mo Post Rdx	8/26/2011	CR (Complete Response) - All lesions gone and tumor markers normal	IR (Indeterminate Response)/SD(Stabl e Disease) - Anything else	PR - Partial Response	
TPF04	4 Tumor Respons e 24mo Post Rdx					

TPF04	Follow Up					PR - per RECIST
TPF14	2 Tumor Respons e 3mo Post Rdx	11/28/2012	CR (Complete Response) - All lesions gone and tumor markers normal		CR - Complete Response	
TPF14	3 Tumor Respons e 12mo Post Rdx	7/24/2013	CR (Complete Response) - All lesions gone and tumor markers normal		CR - Complete Response	
TPF14	4 Tumor Respons e 24mo Post Rdx	7/14/2015	CR (Complete Response) - All lesions gone and tumor markers normal		CR - Complete Response	
TPF14	Follow Up					CR - per RECIST

TPF15	2 Tumor Response 3mo Post Rdx	12/26/2012	CR (Complete Response) - All lesions gone and tumor markers normal		CR - Complete Response	
TPF15	3 Tumor Response 12mo Post Rdx	5/9/2013	PD (Progressive Disease)- Current Sum >20% increase from the best response		PD - Progressive Disease	
TPF15	4 Tumor Response 24mo Post Rdx					
TPF15	Follow Up					CR - per RECIST
TPF23	2 Tumor Response 3mo Post Rdx	2/19/2015	PR (Partial Response)- Current sum >30% decrease from baseline sum		PR - Partial Response	

TPF23	3 Tumor Respons e 12mo Post Rdx	11/18/2015	PR (Partial Response)- Current sum >30% decrease from baseline sum		PR - Partial Response	
TPF23	4 Tumor Respons e 24mo Post Rdx	9/15/2016	PD (Progressive Disease)- Current Sum >20% increase from the best response		PD - Progressive Disease	
TPF23	Follow Up					PR - per RECIST
TPF28	2 Tumor Respons e 3mo Post Rdx	12/5/2016	PR (Partial Response)- Current sum >30% decrease from baseline sum		PR - Partial Response	

TPF28	3 Tumor Response 12mo Post Rdx	12/8/2017	PR (Partial Response)- Current sum >30% decrease from baseline sum		PR - Partial Response	
TPF28	4 Tumor Response 24mo Post Rdx	1/18/2019	CR (Complete Response) - All lesions gone and tumor markers normal		CR - Complete Response	
TPF28	Follow Up					CR - per RECIST

PFS and OS

Record ID	Days survived until death or loss to follow up (Date of Registration to Date of	OS Status (Alive/ Dead)	Days Survived Progression Free (Date of Registration to Date of Progression ,	PFS status (alive-prorgression-free or Progressed/dead)	Date of Registration (Date Informed Consent Obtained)	Date of last contact	Date of Death	Has the disease progressed ?	Date of assessment	Lost to Follow Up ?	Non-Metastatic (MO) / Metastatic (M1)

	Death /LFU)		Death)								
TPF 01	3079	Dead	631	Dead	6/8/2 009		11/12 /2017	Yes	3/1/2 011	No	M1
TPF 02	3735	Alive	3735	Progressi on Free	9/25/ 2009	12/17 /2019		No	12/17 /2019	No	M0
TPF 03	3580	Alive	3580	Progressi on Free	1/5/2 010	10/25 /2019		No	10/25 /2019	No	M0
TPF 04	2713	Dead	2104	Dead	2/19/ 2010		7/25/ 2017	Yes	11/24 /2015	No	M1
TPF 05	3502	Alive	2821	Progressi on Free	3/16/ 2010	10/17 /2019		No	12/5/ 2017	No	M0
TPF 09	2982	Alive	2982	Progressi on Free	9/20/ 2011	11/19 /2019		No	11/19 /2019	No	M0
TPF 10	2984	Alive	2876	Progressi on Free	10/4/ 2011	12/5/ 2019		No	8/19/ 2019	No	M0
TPF 11	2905	Alive	2731	Progressi on Free	11/29 /2011	11/12 /2019		No	5/22/ 2019	No	M0
TPF 12	2634	Alive	2603	Progressi on Free	11/30 /2011	2/15/ 2019		No	1/15/ 2019	No	M0
TPF 13	2938	Alive	475	Progress ed	12/1/ 2011	12/17 /2019		Yes	3/20/ 2013	No	M0
TPF 14	2779	Alive	2750	Progressi on Free	12/2/ 2011	7/12/ 2019		No	6/13/ 2019	No	M1
TPF 15	568	Dead	377	Dead	5/17/ 2012		12/6/ 2013	Yes	5/29/ 2013	No	M1
TPF 16	2923	Alive	2824	Progressi on Free	12/3/ 2011	12/4/ 2019		No	8/27/ 2019	No	M0
TPF 17	2473	Alive	2078	Progressi on Free	3/1/2 013	12/8/ 2019		No	11/8/ 2018	No	M0
TPF 19	2365	Alive	2324	Progressi on Free	5/29/ 2013	11/19 /2019		No	10/9/ 2019	No	M0
TPF 20	2362	Alive	2323	Progressi on Free	6/21/ 2013	12/9/ 2019		No	10/31 /2019	No	M0
TPF 21	2351	Alive	2351	Progressi on Free	7/9/2 013	12/16 /2019		No	12/16 /2019	No	M0
TPF 22	2020	Alive	1992	Progressi on Free	10/3/ 2013	4/15/ 2019		No	3/18/ 2019	No	M0
TPF 23	1924	Alive	751	Progress ed	8/26/ 2014	12/2/ 2019		Yes	9/15/ 2016	No	M1
TPF 24	1747	Alive	819	Progressi on Free	9/18/ 2014	7/1/2 019		No	12/15 /2016	No	M0
TPF 25	1194	Dead	471	Dead	1/21/ 2015		4/29/ 2018	Yes	5/6/2 016	No	M0
TPF 26	1404	Alive	1260	Progressi on Free	1/14/ 2016	11/18 /2019		No	6/27/ 2019	No	M0

TPF 27	1308	Alive	1204	Progression Free	5/12/2016	12/11/2019		No	8/29/2019	No	M0
TPF 28	1292	Alive	1291	Progression Free	6/3/2016	12/17/2019		No	12/16/2019	No	M1
TPF 29	1035	Alive	910	Progression Free	2/2/2017	12/4/2019		No	8/1/2019	No	M0
TPF 30	805	Alive	805	Progression Free	8/28/2017	11/11/2019		No	11/11/2019	No	M0
