# Supplementary material for: In search for optimal induction chemotherapy for advanced nasopharyngeal cancer: Standard dosing of Docetaxel, Platinum, and 5-Fluorouracil (TPF) followed by chemoradiation
Source: PLoS One. 2023 Feb 2;18(2):e0276651. doi: 10.1371/journal.pone.0276651 (PMC9894485; doi:10.1371/journal.pone.0276651)
Supplement: S2 File — (PDF) [file pone.0276651.s002.pdf]

Record ID	Event Name	Neuropatl	Hearing In	Neutrophi	Platelet Co	Hemoglok	Creatinine	Total Billir	Oral Mucc
TPF02	Baseline/Pre-treatment	0	0	0	0	1	0	0	0
TPF02	Induction Treatment TPF 1	0	0	0	0	1	0	0	1
TPF02	Induction Treatment TPF 2	0	0	4	1	1	0	0	1
TPF02	Induction Treatment TPF 3	1	0	0	0	1	0	0	0
TPF02	End of Induction	1	0	0	2	1	0	0	0
TPF03	Baseline/Pre-treatment	0	0	0	0	0	0	0	0
TPF03	Induction Treatment TPF 1	0	0	0	1	0	0	0	0
TPF03	Induction Treatment TPF 2	0	0	0	0	0	0	0	3
TPF03	Induction Treatment TPF 3	1	0	0	1	1	0	0	3
TPF03	End of Induction	0	0	0	0	1	0	0	3
TPF05	Baseline/Pre-treatment	0	0	0	0	1	0	0	0
TPF05	Induction Treatment TPF 1	0	0	0	0	1	0	0	0
TPF05	Induction Treatment TPF 2	0	0	0	0	1	0	0	1
TPF05	Induction Treatment TPF 3	0	0	0	0	1	1	0	1
TPF05	End of Induction	0	0	0	0	1	0	0	1
TPF09	Baseline/Pre-treatment	0	0	0	0	1	0	0	0
TPF09	Induction Treatment TPF 1	0	0	0	0	1	0	0	0
TPF09	Induction Treatment TPF 2	0	0	0	0	2	0	0	1
TPF09	Induction Treatment TPF 3	0	0	0	0	2	0	0	2
TPF09	End of Induction	0	0	2	0	2	0	0	1
TPF09	SAE 01								
TPF09	SAE 02								
TPF10	Baseline/Pre-treatment	0	3	0	0	1	0	0	0
TPF10	Induction Treatment TPF 1	0	3	0	0	1	0	0	0
TPF10	Induction Treatment TPF 2	0	3	0	0	1	0	0	0
TPF10	Induction Treatment TPF 3	0	3	0	0	2	0	0	0
TPF10	End of Induction	0	3	0	0	3	0	0	0
TPF10	SAE 01								
TPF11	Baseline/Pre-treatment	0	3	0	0	0	0	0	0
TPF11	Induction Treatment TPF 1	0	3	0	0	1	0	0	0
TPF11	Induction Treatment TPF 2	0	3	0	0	0	0	0	1
TPF11	Induction Treatment TPF 3	0	3	0	0	1	0	0	0
TPF11	End of Induction	0	3	4	0	1	0	0	0

TPF12	Baseline/Pre-treatment	0	1	0	0	0	0	0	0
TPF12	Induction Treatment TPF 1	0	1	0	0	0	0	0	0
TPF12	Induction Treatment TPF 2	0	1	1	0	1	0	0	0
TPF12	Induction Treatment TPF 3	0	1	0	0	1	0	0	0
TPF12	End of Induction	0	1	0	1	1	0	0	0
TPF13	Baseline/Pre-treatment	0	1	0	0	0	0	0	0
TPF13	Induction Treatment TPF 1	0	1	0	0	0	0	0	0
TPF13	Induction Treatment TPF 2	0	1	0	0	0	0	0	0
TPF13	Induction Treatment TPF 3	0	1	0	0	1	0	0	0
TPF13	End of Induction	0	1	0	0	1	0	0	0
TPF16	Baseline/Pre-treatment	0	0	0	0	0	0	0	0
TPF16	Induction Treatment TPF 1	0	0	0	0	0	1	0	0
TPF16	Induction Treatment TPF 2	0	0	1	0	1	0	0	0
TPF16	Induction Treatment TPF 3	0	0	0	0	1	1	0	0
TPF16	End of Induction	0	0		0	1	0	0	0
TPF17	Baseline/Pre-treatment	0	0	0	0	0	0	0	0
TPF17	Induction Treatment TPF 1	0	0	0	0	0	0	0	0
TPF17	Induction Treatment TPF 2	0	0	0	0	1	0	0	2
TPF17	Induction Treatment TPF 3	2	0	0	0	1	0	0	0
TPF17	End of Induction	1	0	0	0	1	0	0	0
TPF19	Baseline/Pre-treatment	0	0	0	0	0	0	0	0
TPF19	Induction Treatment TPF 1	0	0	0	0	0	0	0	0
TPF19	Induction Treatment TPF 2	0	0	0	0	2	0	0	0
TPF19	Induction Treatment TPF 3	0	0	0	0	2	0	0	0
TPF19	End of Induction	0	0	0	0	2	0	0	0
TPF20	Baseline/Pre-treatment	0	0	0	0	1	0	0	0
TPF20	Induction Treatment TPF 1	0	0	0	0	1	0	0	0
TPF20	Induction Treatment TPF 2	1	0	0	0	1	1	0	0
TPF20	Induction Treatment TPF 3	1	0	0	0	1	1	0	0
TPF20	End of Induction	1	0	0	0	1	0	0	0
TPF21	Baseline/Pre-treatment	0	0	0	1	1	0	0	0
TPF21	Induction Treatment TPF 1	0	0	4	1	1	0	0	0
TPF21	Induction Treatment TPF 2	0	0	0	0	2	0	0	0

TPF21	Induction Treatment TPF 3	0	0	1	2	2	0	0	0
TPF21	End of Induction	0	0	2	1	1	0	0	0
TPF21	SAE 01								
TPF21	SAE 02								
TPF22	Baseline/Pre-treatment	0	0	0	0	1	0	0	0
TPF22	Induction Treatment TPF 1	0	0	0	0	1	0	0	0
TPF22	Induction Treatment TPF 2	0	0	0	0	2	0	0	0
TPF22	Induction Treatment TPF 3	0	0	0	0	2	0	0	0
TPF22	End of Induction	0	0	0	0	1	0	0	0
TPF24	Baseline/Pre-treatment	0	0	0	0	1	0	0	0
TPF24	Induction Treatment TPF 1	0	0	0	0	1	0	0	0
TPF24	Induction Treatment TPF 2	0	0	0	0	1	0	0	0
TPF24	Induction Treatment TPF 3	0	0	0	0	1	0	0	0
TPF24	End of Induction	0	0	0	0	1	0	0	1
TPF25	Baseline/Pre-treatment	1	0	0	0	0	0	0	0
TPF25	Induction Treatment TPF 1	0	0	4	0	0	0	0	0
TPF25	Induction Treatment TPF 2	0	0	0	0	1	1	0	0
TPF25	Induction Treatment TPF 3	0	0	0	0	1	1	0	0
TPF25	End of Induction	0	0	0	0	1	0	0	0
TPF25	SAE 01								
TPF26	Baseline/Pre-treatment	0	0	0	0	0	0	0	0
TPF26	Induction Treatment TPF 1	0	0	0	0	1	0	0	0
TPF26	Induction Treatment TPF 2	0	0	0	0	1	0	0	1
TPF26	Induction Treatment TPF 3	0	0	0	0	1	0	0	2
TPF26	End of Induction	0	0	1	0	1	0	0	2
TPF27	Baseline/Pre-treatment	0	0	0	0	0	0	0	0
TPF27	Induction Treatment TPF 1	0	0	0	0	1	0	0	0
TPF27	Induction Treatment TPF 2	0	0	0	0	1	0	0	0
TPF27	Induction Treatment TPF 3	0	0	0	0	1	0	0	0
TPF27	End of Induction	0	0	0	0	1	0	0	0
TPF29	Baseline/Pre-treatment	0	0	0	0	0	0	0	0
TPF29	Induction Treatment TPF 1	0	0						0
TPF29	Induction Treatment TPF 2	0	0	0	0	1	0	0	2

TPF29	Induction Treatment TPF 3	0	0	0	0	1	0	0	1
TPF29	End of Induction	0	0	0	0	1	0	0	2
TPF30	Baseline/Pre-treatment	0	0	0	0	1	0	0	0
TPF30	Induction Treatment TPF 1	0	0	0	0	1	0	0	0
TPF30	Induction Treatment TPF 2	0	0	3	0	1	0	0	0
TPF30	Induction Treatment TPF 3	0	0	2	0	1	0	0	0
TPF30	End of Induction	0	0	0	0	1	0	0	0

Other AEs (choice=Yes)	Descriptio	AE Grade	Descriptio	AE 2 Grad	Descriptio
Unchecked					
Checked	Constipati	1	Insomnia	1	Rash chee
Checked	Rash	1	Fatigue	1	Alopecia
Checked	Fingers an	1	Diarrhea	2	Altered Ta
Checked	Diarrhea	1			
Unchecked					
Unchecked					
Checked	Trismus	1	Tinitus	1	Palmar-pla
Checked	Nausea	1	Constipati	1	Hyperpigr
Checked	Vomiting	2	Fatigue	2	Weight Lo
Unchecked					
Checked	Headache	1			
Checked	Edema - h	1	Hyperpigr	1	Salivary du
Checked	Fatigue	1	Dry Cough	1	Dysgeusia
Checked	Fatigue	2	Dysgeusia	2	
Unchecked					
Unchecked					
Checked	Neutroper	1	Diarrhea	1	Febriled N
Checked	Weight Lo	1	Alopecia	1	Fatigue
Checked	Weight los	1	Alopecia	1	
Unchecked					
Checked	Cough	1			
Checked	Cough	1	Nausea	1	Vomiting
Checked	Cough	1			
Checked	Hypokaler	4	Dehydrati	2	Nausea
Unchecked					
Unchecked					
Checked	Lymphocy	3			
Checked	Lymphocy	3	Dry moutl	2	Dysgeusia
Checked	White blo	3	Lymphocy	2	Dry moutl

Unchecked					
Unchecked					
Checked	Anorexia	2	Lymphocy	2	Nausea
Unchecked					
Checked	Fatigue	2			
Unchecked					
Unchecked					
Unchecked					
Checked	Diarrhea	1	Fatigue	2	
Unchecked					
Unchecked					
Checked	Fatigue	2			
Unchecked					
Unchecked					
Unchecked					
Unchecked					
Unchecked					
Checked	Diarrhea	3			
Unchecked					
Unchecked					
Unchecked					
Unchecked					
Checked	Hypoalbu	2	Lymphocy	2	
Checked	Lymphocy	2	Hyperglyc	2	
Checked	Lymphocy	2	Hyperglyc	2	
Unchecked					
Checked	Alopecia	2	Nausea	2	Paresthes
Checked	Lymphocy	3	Hyperglyc	3	Alopecia
Checked	Hyperglyc	3	Alopecia	2	Paresthes
Checked	Alopecia	2	Paresthes	2	Lymphocy
Unchecked					
Checked	Typhlitis	3	Thrush	2	Abdomina
Checked	Diarrhea	2	Hypoalbu	2	White blo

Unchecked						
Checked	White blo	2				
Unchecked						
Unchecked						
Checked	Vomitting	2				
Unchecked						
Unchecked						
Checked	Dysgeusia	2	Fatigue	1	Abdomina	
Checked	Dysgeusia	2	Fatigue	1	Nausea	
Checked	Dysgeusia	2	Fatigue	1	Nausea	
Checked	Dysgeusia	2	Fatigue	1	Nausea	
Checked	Dysgeusia	2	Fatigue	1	Nausea	
Checked	Puritic Ras	1				
Checked	Puritic Ras	1				
Checked	Puritic Ras	1	Hiccups	1	Acid Reflu	
Checked	Dysgeusia	1	Abdomina	1	Hair Loss	
Checked	Dysgeusia	1	Abdomina	1	Hair Loss	
Unchecked						
Checked	Somnolen	3	Fatigue	2	Lymphocy	
Checked	Shortness	1	Fatigue	2	Alopecia	
Checked	Dysgeusia	1	Nausea	1	Alopecia	
Checked	Dysgeusia	1	Alopecia	1	Ringing in	
Unchecked						
Checked	Lymphocy	2	Hypoalbu	2		
Unchecked						
Unchecked						
Unchecked						
Unchecked						
Unchecked						
Checked	Dermatitis	2				

Checked	Hyperglyc	2		
Unchecked				
Unchecked				
Checked	Lymphocy	3		
Checked	White blo	3	Lymphocy	3
Checked	White blo	3		
Unchecked				

AE 3 Grad	Descriptio	AE 4 Grad	Descriptio	AE 5 Grad	Descriptio	AE 6 Grad	Descriptio	AE 7 Grad	Descriptio	AE 8 Grad	Descriptio	AE 9 Grad	Descriptio
1	Muscle W	2	Dyspnea	1									
1													
1													
2	PICC Line-	1	Dysgeusia	1	Fatigue	1	Alkaline P	1	Alanine A	2	Aspartate	1	Insomnia
1	Palmar-pl	3	Alopecia	2									
2													
1	Nausea	1											
2	Oral Pain	1											
3													
1	Dysplagia	1	Electrolyt	1	Dehydrati	1	Acute Ren	1	Febrile Ne	1	CNS in Blo	1	Febrile ne
1													
1	Vomiting	1	Diarrhea	1	Fatigue	1							
2													
2	Dysgeusia	2											

2				
2				
2	Nausea	2	Paresthes	2
2				
2				
2	Dysphagia	2		
2	Thromboc	2	febrile ne	3

2													
1	Lymphocy	2											
1	Lymphocy	2											
1	Alopecia	1	Lymphocy	2									
1	Upper nec	1	Alocpecia	1									
1	Fevers anc	1	Diaphores	1	Candida	1	Abdomina	1	Hair Loss	1	Loss of ap	1	Dehydrati
1	Loss of Ap	1	Tachycard	1	Constipati	1	Weight Lo	2	Hiccups	1			
1	Loss of Ap	1	Tachycard	1	Constipati	1	Weight Lo	2	Hiccups	1	Dysgeusia	2	
2	Skin infect	2											
1	Dysgeusia	1	GERD	1	Bloody Nc	1	Skin Infect	2					
1	Stomach c	1	Ringing in	1									
1													

AE 10 Gra	Descriptio	AE 11 Gra	Descriptio	AE 12 Gra	Descriptio	AE 13 Gra	Descriptio	AE 14 Gra	Descriptio	AE 15 Gra	Complete	Description of AE 16
											Complete	
											Complete	
											Complete	
											Complete	
											Complete	
											Complete	
											Complete	
1											Complete	
											Complete	
											Complete	
											Complete	
											Complete	
											Complete	
											Complete	
											Complete	
											Complete	
											Complete	
											Complete	
3											Complete	
											Complete	
												Febrile Neutropenia
												Febrile neutropenia
											Complete	
											Complete	
											Complete	
											Complete	
											Complete	
											Complete	
											Complete	
											Complete	
											Complete	
											Complete	

Complete
Complete
Complete
Complete
Complete
Complete
Complete
Complete
Complete
Complete
Complete
Complete
Complete
Complete
Complete
Complete
Complete
Complete
Complete
Complete
Complete
Complete
Complete
Complete
Complete
Complete
Complete
Complete
Complete
Complete
Complete
Complete
Complete

							Complete	
							Complete	
								Thrombocytopenia
								Febrile Neutropenia
							Complete	
							Complete	
							Complete	
							Complete	
							Complete	
							Complete	
							Complete	
							Complete	
							Complete	
							Complete	
							Complete	
							Complete	
2	Tachycard	1	Coronavir	3	Weight Lo	1	Complete	
							Complete	
							Complete	
								Blood and Lymphatic Syst
							Complete	
							Complete	
							Complete	
							Complete	
							Complete	
							Complete	
							Complete	
							Complete	
							Complete	
							Complete	
							Complete	
							Complete	
							Complete	

Complete
Complete
Complete
Complete
Complete
Complete
Complete

AE 16 Grade	Attribution of SAE	Attributio	Attributio	Attributio	Attribution of SAE (choice=None)	Has the e\	Date SAE ı	Comment
3	Checked	Checked	Unchecke	Checked	Unchecked	Yes	#######	
3	Checked	Checked	Unchecke	Checked	Unchecked	Yes	#######	

2	Checked	Checked	Unchecke	Checked	Unchecked	Yes	#######
3	Checked	Checked	Unchecke	Checked	Unchecked	Yes	#######
3	Unchecked	Unchecke	Unchecke	Checked	Unchecked	Yes	

Complete?

Complete
Complete

Complete
Complete

Complete
