# Supplementary material for: In search for optimal induction chemotherapy for advanced nasopharyngeal cancer: Standard dosing of Docetaxel, Platinum, and 5-Fluorouracil (TPF) followed by chemoradiation
Source: PLoS One. 2023 Feb 2;18(2):e0276651. doi: 10.1371/journal.pone.0276651 (PMC9894485; doi:10.1371/journal.pone.0276651)
Supplement: S3 File — (PDF) [file pone.0276651.s003.pdf]

Version date August 13, 2008

NCI CTEP protocol number, 8209.

Stanford protocol # ENT 0025

TITLE: A Phase 2 Study of Sequential and concurrent chemoradiation for patients with advanced nasopharyngeal carcinoma (NPC)

.

Coordinating Center:

Stanford Cancer Center

Stanford CA 94305

Participating Centers:

UCSF Cancer Center, San Francisco, CA

Principal Investigator:

A. Dimitrios Colevas, M.D.

Associate Professor of Medicine (Oncology)

Stanford University Medical Center

875 Blake Wilbur Drive

Stanford, CA 94305-5826

(650) 724-9707

FAX (650) 498-5800

colevas@stanford.edu

Co- Principal Investigator:

Quynh-Thu Le, MD

Professor

Clinical Research Director
Department of Radiation Oncology
Stanford University
Tel 650-498-5032
Fax 650-725-8231
email qle@stanford.edu

Co-Investigators:

Sue S. Yom, MD, PhD
Assistant Professor of Clinical Radiation Oncology
UCSF Helen Diller Family Comprehensive Cancer Center
UCSF School of Medicine
Department of Radiation Oncology
1600 Divisadero St., Suite H-1031
San Francisco, CA 94143-1708
Email: yoms@radonc.ucsf.edu
Office: 415-353-7410
Fax: 415-353-9883

Sarita Dubey, MD
Clinical Assistant Professor of Medicine
UCSF Helen Diller Family Comprehensive Cancer Center
UCSF School of Medicine
1600 Divisadero St, Box 1770
San Francisco, CA 94143
PH: 415-353-9674
Fax: 415-353-9959
sdubey@medicine.ucsf.edu

Nuclear medicine co- investigator

Andrew Quon, MD
Assistant Professor of Radiology
Stanford University Medical Center

300 Pasteur Drive H-0101
Stanford, California 94305-5281
(650) 736-1369
aquon@stanford.edu

Statistician:
Alex McMillan PhD
Sr Res Scientist-Basic Ls *in* Health Research and Policy – Biostatistics
Redwood Bldg, Stanford, California 94305
(650) 725-5352
Alex.McMillan@stanford.edu

Study Coordinator and data manager:
Ruth Lira
Stanford University Medical Center
875 Blake Wilbur Drive
Stanford, CA 94305-5826
Telephone (650) 723-1367
Fax (650) 498 5800
e-mail address rlira@stanford.edu

SCHEMA

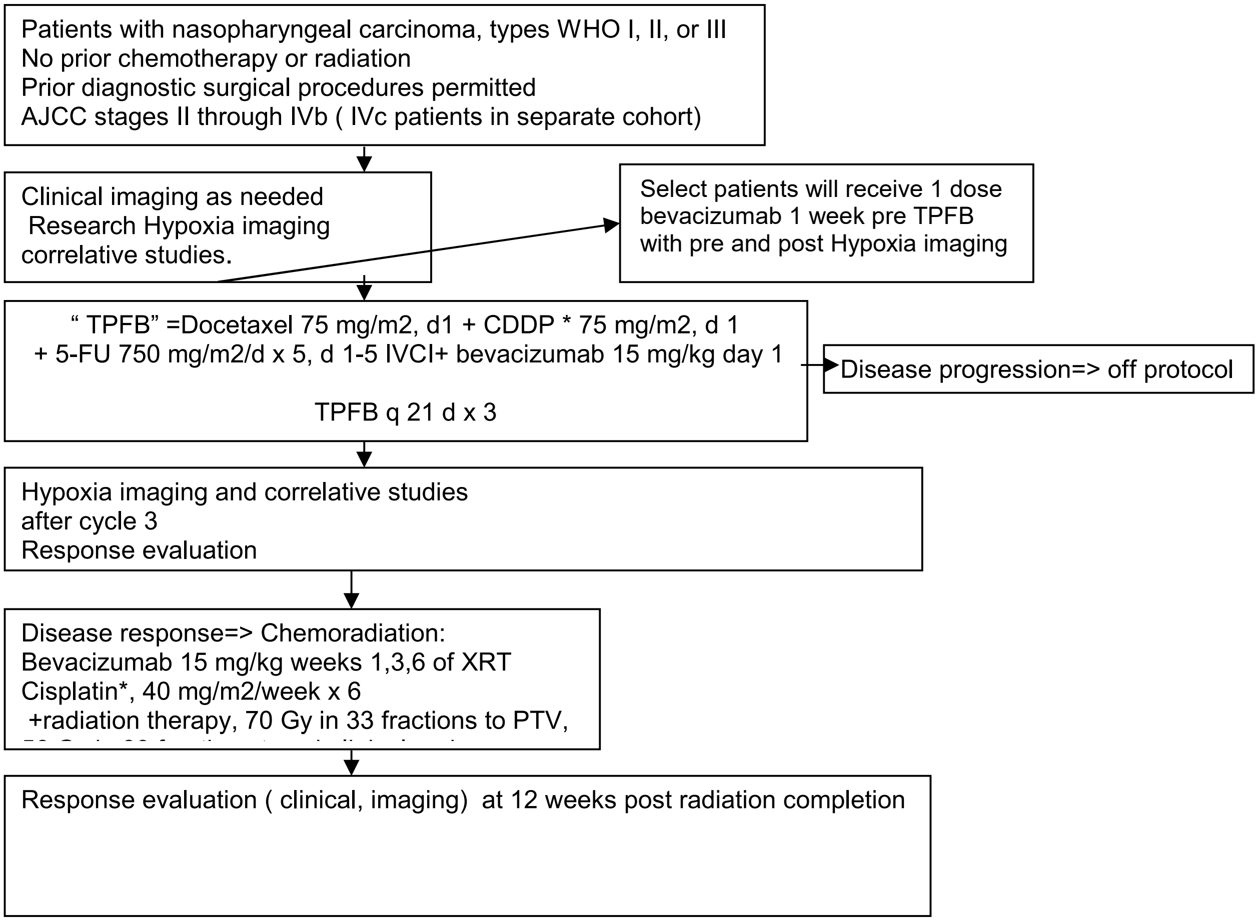

* Under certain circumstances, carboplatin AUC 6 may be substituted for CDDP in TPF and carboplatin AUC 1.5 may be substituted for CDDP during radiation. See the protocol for specific circumstances under which this is permitted.

TABLE OF CONTENTS

Page

SCHEMA ....

1. OBJECTIVES ....
1.1 **Primary Objectives** ....
1.2 **Secondary Objectives** ....

2. BACKGROUND ....
2.1 **Nasopharyngeal Cancer** ....
2.2 **EBV and NPC** ....
2.3 **Chemoradiation vs Radiation** ....
2.4 **Adjuvant vs Neoadjuvant chemotherapy** ....
2.5 **Carboplatin vs Cisplatin** ....
2.6 **Taxanes** ....
2.7 **VEGF and Bevacizumab in NPC** ....
2.8 **Tumor Hypoxia and NPC** ....
2.9 **Radiation and NPC** ....
2.10**Rationale** ....
2.11 **Correlative Studies Background** ....
 2.11.1 Hypoxia imaging in NPC ....
 2.11.2 Serum EBV DNA ....

3. PATIENT SELECTION ....
3.1 **Eligibility Criteria** ....
3.2 **Exclusion Criteria** ....
3.3 **Inclusion of Women and Minorities** ....

4. REGISTRATION PROCEDURES ....
4.1 **General Guidelines** ....
4.2 **Registration Process** ....

5. TREATMENT PLAN ....
5.1 **Prophylactic Gastrostomy Feeding Tube** ....
5.2 **Agent Administration** ....
5.3 Radiation therapy treatment plan...
5.4 **Duration of Follow Up** ....

6. DOSING DELAYS/DOSE MODIFICATIONS ....
6.1 **Bevacizumab Dose Modification**……………………………………………………..
6.2 **Docetaxel Dose Modification**…………………………………………………………
6.3 **Cisplatin and Carboplatin Dose Modification**………………………………………
6.4 **5-Fluorouracil (5-FU) Dose Modification**……………………………………………

7. ADVERSE EVENTS: LIST AND REPORTING REQUIREMENTS ....
7.1.1 Bevacizumab **Comprehensive Adverse Events and Potential Risks Lists (CAEPR)**
7.1.2 F- MISO **Comprehensive Adverse Events and Potential Risks Lists (CAEPR)**
7.2 **Adverse Event Characteristics for commercial agents** ....
7.2.1Docetaxel
7.2.2Cisplatin
7.2.3Carboplatin
7.2.4 5-Fluorouracil
7.3 **Adverse Event Characteristics**
7.4 **Expedited Adverse Event Reporting** ....
7.5 **Routine Adverse Event Reporting**..
7.6 **Secondary AML/MDS** ....

8. PHARMACEUTICAL INFORMATION ....
8.1 ***CTEP-Supplied Investigational Agent(s)*** **Bevacizumab** (rhuMAb VEGF, Avastin™) NSC # 704865
8.2 ***Commercial Agent(s)*** Docetaxel, Cisplatin, Carboplatin, and 5- Fluorouracil.
Note that the F-MISO imaging agent will be used under an IND held by the NCI , but obtained from Cardinalhealth. ....

9. CORRELATIVE/SPECIAL STUDIES ....

9.1 **Hypoxia Imaging** ....
9.2 **Plasma EBV DNA levels** ....

10. STUDY CALENDAR
11. MEASUREMENT OF EFFECT
11.1**Antitumor Effect – Solid Tumors** ....
1**2. DATA REPORTING / REGULATORY CONSIDERATIONS** ....
12.1 **Data Reporting** ....
12.2 **CTEP Multicenter Guidelines** ....
12.3 **Cooperative Research and Development Agreement (CRADA)/**
Clinical Trials Agreement (CTA) ....

13.STATISTICAL CONSIDERATIONS ....
13.1 **Study Design/Endpoints**……………………………………………………...
13.2 **Sample Size/ Accrual Rate**...
13.3 **Stratification Factors**…………………………………………………………
13.4 **Analysis of Secondary** endpoints………………………………………..
13.5 **Reporting** …………………………………………………………….
...... 13.5.2 Evaluation of Response…………………………………………………
REFERENCES...

APPENDICES

Appendix A : performance status criteria
Appendix B: Hypoxia in GMB, effect of bevacizumab poster
Appendix C:F MISO PET imaging manual
Appendix D: CTEP Multicenter guidelines
Appendix E: Radiation quality assurance form
Appendix F Registration and data submission forms ....

1. OBJECTIVES

1.1. **Primary Objectives: To establish the complete response rate using RECIST criteria, to TPFB followed by chemoradiotherapy of locoregionally advanced nasopharyngeal carcinoma.**

1.2. **Secondary Objectives**

1.2.1. **Evaluate safety and feasibility of TFPB=> chemoxrt in patients with NPC**

1.2.2. **Evaluate early tumor hypoxia changes to bevacizumab and later hypoxia changes to TPFB, with preliminary correlation with CR rates and PFS.**

1.2.3. **Evaluate changes in serum EBV DNA levels following bevacizumab and TPFB with preliminary correlation with CR rates and PFS.**

2. BACKGROUND

Background for the rationale of TPFB=> chemoradiation

2.1 Nasopharyngeal cancer

Nasopharyngeal cancer (NPC)is a significant problem worldwide. The annual incidence rate worldwide is 1.8/100,000 and the rate climbs to as high as 50/100,000 in Southern China .[1]

2.2 EBV and NPC

The association between Epstein- Barr virus (EBV) infection and NPC is well known. In areas of high NPC incidence, evaluation of high IgA antibodies to EBV capsid antigen and early antigen have been proposed as a potential screening tool. Screening using Epstein-Barr virus (EBV) IgA and DNA assays has been shown to be highly sensitive and specific in a high risk population . [2] Pretherapy plasma EBV DNA levels are an independent prognostic indicator in NPC patients treated exclusively with radiation.[3]Assays measuring post treatment circulating EBV DNA levels have been shown to be predictive of outcome in patients treated with radiation or chemoradiation , with a 92 and 37 percent 2 year freedom from relapse for patients with undetectable versus detectable plasma EBV DNA respectively. [4] Therefore, while there are data to suggest that plasma DNA is a predictor of outcome in patients with NPC, these data are confined to baseline EBV DNA in patients treated with radiation alone or after the completion of radiation in a mixed set of patients. It is unknown whether plasma EBV level alteration after chemotherapy alone is of predictive utility in patients with NPC.

2.3 CHEMORADIATION VERSUS RADIATION

Historically, nonmetastatic NPC has been treated with radiation alone. Because of the known chemosensitivity of NPC, a series of randomized controlled trials of chemoradiotherapy versus radiotherapy conducted in the 1990's and early 2000's demonstrated that in patients with advanced local or regional disease, the addition of chemotherapy to radiation was associated with a statistically significant and clinically meaningful survival advantage. [5 6, 7] A smaller subsequent trial suggested that even in the

case of patients with advanced local disease but minimal nodal disease (T3-4, N0-1), patients had increased local control with the use of combined chemotherapy and accelerated fractionation radiation versus conventional radiation alone. [8] The table below is a summary of the RCT data supporting either an OS or LRC advantage of chemoradiation versus radiation:

From IJROBP Volume 66, Number 1, 2006 p 150 [8]:

Table 8. Literature reports on concurrent ± adjuvant chemoradiotherapy and/or accelerated radiotherapy for nasopharyngeal carcinoma

	Stage		Chemotherapy concurrent ±	Time point	Tumor control					Toxicity	
Author	AJCC-5	Fractionation	adjuvant	(year)	FFS (%)	PFS (%)	LR-FFR (%)	D-FFR (%)	OS (%)	Acute (Crude %)	Late (Actuarial %)
Phase III trials comparing concurrent chemoradiotherapy vs. radiotherapy alone											
Al-Sarraf (2, 15)	II-IVB	All CF	P + PF	5		58 vs. 29*			67 vs. 37*	76 vs. 50*	NR
Lin (17)	II-IVB	All CF	PF	5	72 vs. 53*		89 vs. 73* (L)	79 vs. 70[†]	72 vs. 54*	S	NR
Chan (18)	II-IVB	All CF	P	5	60 vs. 52		NS	NS	70 vs. 59[†]	S	NR
Kwong (19)	II-IVB	All CF	U ± PF/VBM	3	69 vs. 58		80 vs. 72	85 vs. 71*	87 vs. 77[†]	S	NR
Wee (16)	III-IVB	All CF	P + PF	3		72 vs. 53*	NR	87 vs. 70*[§]	80 vs. 65*	S	NR
NPC-9901 (3)	T1-4N2-3	All CF	P + PF	3	72 vs. 62*	70 vs. 61	92 vs. 82*	76 vs. 73	78 vs. 78	84 vs. 53*	28 vs. 13*
NPC-9902	T3-4N0-1	CF Arm	P + PF	3	74 vs. 70	73 vs. 68	81 vs. 85	89 vs. 81	87 vs. 83	82 vs. 55*	31 vs. 14
Phase III trials comparing accelerated fractionation vs. conventional fractionation											
Teo (20)	T1-4N0-2	AF	Nil	5	85 vs. 77		89 vs. 85 (L)	93 vs. 85	85 vs. 87	91 vs. 42*	49 vs. 23*[‡]
NPC-9902	T3-4N0-1	AF	Nil	3	63 vs. 70	63 vs. 68	78 vs. 85	77 vs. 81	73 vs. 83	69 vs. 55	22 vs. 14
Series treated by combined strategies of concurrent chemoradiotherapy and accelerated fractionation											
Lin (21)	II-IVB	HF	PF + PF	3	64		89 (L)	74	74	>61	
Wolden (23)	II-IVB	AF	P + PF	3		66	89 (L)	79	84	>84	
NPC-9902	T3-4N0-1	AF	P + PF	3	94	88	94	97	88	86	34

Abbreviation: FFS = failure-free survival (failure at any site); PFS = progression-free survival (failure or death); LR-FFR = locoregional failure-free rate; D-FFR = distant failure-free rate; OS = overall survival (death from any cause); L = local failure-free rate alone; CF = conventional fractionation; AF = accelerated fractionation; HF = hyperfractionation; P = cisplatin; F = 5-fluorouracil; UFT = uracil and tegafur; VBM = combination of vincristine, bleomycin and methotrexate; NR = not reported; NS = nonsignificant hazard ratio, but no data on actuarial rate; S = statistically significant, but no corresponding data on overall rate.

* Statistically significant ($p < 0.05$).

[†] Borderline significance ($p = 0.05–0.08$).

[‡] Neurologic damages only.

[§] 2-year incidence of freedom from distant failure as the first site of failure.

The advantage to chemoradiation over radiation in the RCTs was distributed between both local and systemic effects. In the large RCT cited above, there was a substantial reduction in both locoregional recurrence and metastatic disease in the combined treatment arms versus radiation alone.

In all of the RCT, the chemotherapy combined cisplatin – based chemotherapy with XRT, with cisplatin dosing plans including 40 mg/m2 weekly to 100 mg/m2 every 3 weeks to 20mg/m2/d x 4 with concurrent 5FU infusion every 3 weeks. In the US intergroup study, an additional 3 cycles of cisplatin 80mg/m2 plus 5FU 1000mg/m2/d x 4 days was planned, to be administered after radiation had been completed.

A common theme to all of these recipes is that in all cases cumulative dose of concurrent cisplatin exceeded 180mg/m2. However , in the US intergroup trial, where 300 mg/m2 (three 100 mg/m2 doses) was the intent, only 63% of the patients received three cycles of CDDP. In the Chan et al. trial in which CDDP was administered weekly to an anticipated total of 240 mg/m2, 95% of the patients were compliant with the plan. [9] Therefore, while the optimal concurrent CDDP dosing schedule has yet to be defined, it appears that weekly dosing is more tolerable and in many cases dosing beyond a cumulative dose of 200-240 mg/m2 is not feasible. Additionally, of the 3 RCT discussed here, only one, the US intergroup study, administered adjuvant PF. Only about half of the patients randomized to the adjuvant PF were able to receive 3 cycles, and a third of the patients randomized to that arm received no adjuvant treatment. Therefore, since all 3 RCTs showed an overall survival advantage for patients with advanced disease, it is not clear that adjuvant PF has benefit beyond concurrent chemoradiation, nor is it clear that administration of PF after chemoradiation is feasible.

2.4 Adjuvant versus neoadjuvant(or induction) chemotherapy

The rationale behind administration of adjuvant PF in the intergroup trial was that the additional chemotherapy might reduce the number of distant relapses and therefore offer benefit beyond the concurrent treatment. However, a comparison of the outcome of the US intergroup versus the Chan and Lin trials suggests that the reduction in the development of distant metastasis was approximately 30 to 50% in patients with advanced disease on all of these trials. Therefore the benefit of adjuvant PF on this basis is also questionable. Again, this may be because so few patients in the US intergroup study were able to receive meaningful doses of PF after chemoradiation.

Initial studies of induction chemotherapy added to radiation alone in NPC patients failed to show disease control or survival benefit. [10] Recent pooled analysis of cisplatin- based induction chemotherapy versus radiation alone in NPC patients demonstrated improvement in relapse free and disease specific survival, but did not show an overall survival advantage.[11] Subset analysis of these trials demonstrated a survival advantage of induction chemotherapy for patients with early stage, but not advanced stage disease. This subset analysis suggested that in all groups there was a numerical advantage to induction in terms of distant metastasis free survival in all groups , but because locoregional disease was the major contributor to relapse, it overshadowed any potential benefit to systemic control of disease. Therefore, with the development of better radiation techniques to control locoregional disease, we hypothesize that a more effective treatment of distant disease may translate into an OS advantage now that locoregional control rates have improved.

There are ongoing RCT designed to definitively answer the question concerning the possible benefit of the addition of PF induction chemotherapy to concurrent chemoradiation in patients with NPC. Lee et al. of the Hong Kong Nasopharyngeal Cancer Study Group are conducting a trial whose primary endpoint is a comparison of induction chemotherapy with Cisplatin + 5-Fluorouracil versus adjuvant chemotherapy with Cisplatin + 5-Fluororacil (PF-P vs P-PF) in the setting of concurrent chemoradiation as a backbone. This trial opened in September 2006 and is planning to enroll 798 patients with an estimated completion date of September 2013. See web site **ClinicalTrials.gov, Identifier:** NCT00379262. Feng et al. of the Taiwan National Health Research Institutes opened in 2003 a multicenter Phase III Trial Comparing Induction Mitomycin, Epirubicin, Cisplatin, Fluorouracil, and Leucovorin Chemotherapy Followed by Concurrent Chemoradiotherapy Versus Concurrent Chemoradiotherapy Alone in Stage IV Nasopharyngeal Carcinoma (NPC), based on 5 year OS of 70% and 5 year distant metastasis rate of 81% in a phase 2 trial. [12] See ClinicalTrials.gov identifier NCT00201396. This 480 patient trial is expected to be completed in 2013.

Therefore, the question of induction plus concurrent chemoradiation using CDDP based chemotherapy in patients with NPC, while still open, should be answered in the next 5 years with 2 large RCT, at least for the specific chemotherapy recipes discussed above

2.5 Carboplatin versus Cisplatin

Carboplatin is probably as efficacious as cisplatin in the treatment of NPC in the curative setting. Evidence for equivalence can be drawn be inference from other diseases and from one recently published robust direct comparison of CDDP and carboplatin in NPC,

discussed below.

Multiple studies in NSCLC and ovarian cancer have demonstrated that despite the fact that cisplatin containing regimens often have been associated with higher response rates, carboplatin in almost every study is associated with the same survival and better tolerability. [13 14-18]

A randomized controlled trial of patients with SCCHN compared weekly carboplatin (100 mg/m2/dose x 4) with daily low dose cisplatin (4mg/m2/dose, cumulative dose 64 mg/m2) , both concurrently administered with definitive radiation (65Gy). Both local control and overall survival were numerically superior in the carboplatin arm, but OS did not reach statistical significance because of the size of the trial, 119 patients. [19] Because many experts regard the dose of CDDP in this trial as inadequate, one can conclude that there is evidence that carboplatin XRT is associated with a better outcome than XRT and suboptimal CDDP dosing, but is silent on the question of standard CDDP dosing in this setting.

A recent study directly compared CDDP versus carboplatin in the curative setting in patients with NPC who were receiving concurrent definitive radiation. In this 206 patient study, the standard US intergroup concurrent plus adjuvant chemoradiation was compared to an identical radiation plan with carboplatin 100mg/m2 weekly instead of concurrent cisplatin, and carboplatin AUC5 instead of cisplatin in the adjuvant setting. [20] There was no difference in overall survival or disease free survival. Toxicity was markedly less in the carboplatin arm, and over twice as many patients in the carboplatin arm (62 versus 26%) completed all intended chemotherapy treatment.

2.6 TAXANES

Taxanes are among the most active anti- cancer agents available for squamous cell carcinoma of the head and neck, with single agent response rates of 40% or higher reported in patients with prior platinum exposure. [21, 22] Three recently reported RCT of the addition of a taxane to the " backbone" PF induction regimen as part of a curative chemoradiation plan have demonstrated that overall survival with TPF is superior to PF when used as induction chemotherapy followed by either radiation alone or chemoradiation in patients with squamous cell cancer of the head and neck. [23 24, 25] Paradoxically, the three drug combination (taxane, platinum, 5-FU) has been associated with a superior QOL than the two drug combination (platinum, 5-FU). This is probably because the dose of infusional 5-FU, which induces severe gastrointestinal toxicity, was reduced in all versions of the three drug regimen tested. These results let the FDA to recently (9/28/07) approve the use of docetaxel explicitly in combination with cisplatin and 5-fluorouracil for induction therapy of locally advanced squamous cell carcinoma of the head and neck (SCCHN) before patients undergo chemoradiotherapy and surgery. [26] Additionally, a recent meta- analysis of 5 RCT suggests that TPF was associated with a robust 20% two year survival improvement over PF in this setting. [27]

This strategy of using a taxane and cisplatin combination is beginning to be tested in NPC. The combination of docetaxel 75 mg/m2 plus cisplatin 75 mg/m2 achieved a response rate of 63% in patients with metastatic NPC [28]and when docetaxel has been

combined with PF as induction chemotherapy for NPC patients in early phase 1 and 2 trials, response rates approaching 100% have been seen . [29-32]

2.7 VEGF and bevacizumab in NPC

Increased vascular endothelial growth factor-A (VEGF-A) expression has been associated with poor prognosis in squamous cell carcinoma of the head and neck. A meta-analysis of twelvestudies evaluating VEGF-A expression in 1002 patients with head and neck squamous cellcarcinoma showed that positive VEGF staining was associated with an almost two-fold higher risk of death at two years. [33] VEGF has been shown to play an important role in lymph node metastasis through the induction of angiogenesis in nasopharyngeal carcinoma. [34] Qian, et al. have demonstrated that the levels of serum VEGF are significantly elevated in patients with metastatic nasopharyngeal carcinoma. [35] Overexpression of VEGF was seen in 67% of NPC patients and the higher expression of VEGF in Epstein Barr Virus (EBV) positive tumors was related to higher rate of recurrence, nodal positivity, and lower survival. [36] A recent pilot study by Druzgal, et al. analyzed the pre- and post-treatment serum levels of cytokines and angiogenesis factors as markers for outcome in patients with head and neck cancer. [37] In this study, patients were more likely to remain disease free when the VEGF level decreased post-treatment versus those who continued to have increased VEGF levels after treatment.

Bevacizumab, a monoclonal antibody directed against VEGF, has already established clinical utility in colorectal and NSCL cancers in combination with cytotoxic chemotherapy. There are several early clinical trials underway of bevacizumab in combination with chemotherapy and radiation for patients with SCCHN, NSCLC, and various gastrointestinal cancers. The RTOG is presently conducting a phase two trial in NPC patients, adding bevacizumab to the standard US intergroup treatment regimen. We are unaware of any studies of bevacizumab in combination with TPF induction chemotherapy in any SCCHN type, including NPC.

2.8 Tumor Hypoxia and NPC

It is well established that tumor hypoxia is a poor prognostic marker and predicts for decreased radiation response in advanced SCCHN. [38, 39] Increased vascular leakage from immature tumor blood vessels can result in higher interstitial fluid pressure, thereby, worsening tumor hypoxia and impeding effective tumoral drug delivery as well as impeding delivery of oxygen to the tumor. Jain et al. has popularized the concept of normalization of tumor vasculature through anti-angiogenic therapy such as bevacizumab. [40] Preclinical models confirmed that DC 101, a VEGFR2 specific monoclonal antibody could restore intra-tumoral microvasculature, improved tumor oxygenation and enhanced radiation sensitivity. [41]This concept was similarly supported by clinical data in colorectal, breast and brain cancers, where treatment with bevacizumab, paclitaxel (a chemotherapy with anti-angiogenic properties) or AZD2171 (a VEGF receptor tyrosine kinase inhibitor), respectively, was shown to reduce tumor hypoxia and interstitial pressure in human tumors. [42-44] Recent data suggest that in humans with glioblastoma multiforme,

bevacizumab alone, even after a single dose, can not only alter blood flow by DCE MRI assessment but can also reduce intra-tumoral hypoxia as detected by FMISO PET scanning (Appendix B). This concept has yet to be tested in head and neck cancers in general and NPC specifically. We hypothesize that initial treatment with bevacizumab in addition to TPF may enhance drug delivery and improve hypoxia, which has been documented to exist in NPC patients with Fluorine-18 fluoromisonidazole (FMISO, a hypoxia specific tracer) Positron Emission Tomography (PET) imaging . [45] Reductions in tumor hypoxia have been seen in NPC patients treated with radiation. We will use FMISO PET CT to assess the level of tumor hypoxia pretreatment, after one single treatment with bevacizumab and after completion of induction chemotherapy in order to determine the impact of B alone and TPFB on hypoxia modulation in NPC patients prior to chemoradiation.

2.9 Radiation and NPC

For many years, two-dimensional radiation therapy (2DRT) was considered the standard of care for patients with locally advanced NPC. Because 2DRT delivers substantial radiation doses to the parotid glands, permanent severe xerostomia is a common side effect. Radiation doses as low as 15 Gy can result in permanent dysfunction of the major salivary glands. [46, 47]. The permanent xerostomia or oral dryness often results in dysphasia and poor speech function as well as predisposes the patients to fissures, ulcers, dental caries, infection, and in worst cases, osteoradionecrosis. [48 49].

In addition to the parotid glands, there are multiple critical normal tissues surrounding the nasopharynx, such as the optic structures, the temporal lobes and the brain stem, all of which are highly sensitive to radiation injury. The location of such structures precludes dose escalation with 2DRT. Therefore, despite the addition of chemotherapy, the local control rates for the more advanced T3/T4 tumors were typically in the range of 40-60% for 2DRT. [50, 51 52 38, 53] With the introduction of three- dimensional radiation therapy (3DRT) and more recently intensity modulated radiation therapy (IMRT), superior tumor coverage was achieved without exceeding the radiation tolerance to surrounding critical structures.[54-56] Several studies have also compared IMRT to 3DRT plans and demonstrated that IMRT consistently improves tumor target volume coverage while simultaneously significantly reducing radiation exposure to normal structures, in particular the parotid glands, in patients with locally advanced. [57-59] Since then, several centers have reported decreased rates of xerostomia in nasopharyngeal cancer (NPC) patients treated with IMRT[60, 61, 62] There are emerging randomized trial data which confirm the advantage of IMRT in improving salivary gland flow when compared to conventional RT in early stage NPC patients. [63, 64] Besides the dosimetric advantages, several centers also reported excellent early clinical outcomes in NPC for IMRT. [65] The most mature IMRT clinical data came from UCSF[60] The 4-year local progression-free and regional progression-free rates for 67 loco-regional advanced NPC patients were 97% and 98%, respectively. An update with more patients (n=118), continued to show excellent locoregional control. [60] Several centers from Hong Kong also have shown similar findings. [61, 65] A recent experience from MSKCC reported a 91% locoregional control rate for IMRT treated NPC with a median follow-up of 35 months. [62] The RTOG completed a

phase II trial of IMRT with or without chemotherapy for non-stage IVC NPC. Preliminary data showed decreased xerostomia when compared to historical RTOG trials where conventional RT was used. (Lee N, ASTRO proceedings 2007). Based on these results, we propose to use IMRT using the dose and fractionation schedule (70.2 Gy over 6.5 weeks) that has been pioneered at UCSF and validated in the Phase II RTOG study for the radiation treatment of these patients.

2.10 **Rationale**

Please see the background section for the scientific and clinical basis for this trial. Briefly, this trial is intended to ask several questions concerning a new treatment paradigm for patients with locoregionally advanced NPC. While many of the questions posed will only be definitively answerable in larger controlled trials, there is a need to generate preliminary data supporting the below hypotheses before there will be adequate enthusiasm for dedicating the resources of a multi-institutional, multi-national effort to ask the questions which would definitively address these questions.

Hypothesis which this trial will address:

Primary:

Sequential PTFB=> chemoradiation will be associated with a higher complete response rate than the present US standard of care for this group of patients with NPC, chemoradiation=> PF.

Secondary:

Sequential PTFB=> chemoradiation for patients with NPC is more feasible than chemoradiation=> PF as administered by the US intergroup 0099 study as measured by the percentage of patients who are able to complete the planned total course of treatment.

Induction TPFB is more active than PF and better tolerated, as assessed by complete response rate after chemotherapy and incidence and severity of adverse events during chemotherapy.

The addition of bevacizumab to treatment prior to radiation may render tumors less hypoxic.

The diminished hypoxia from TPFB may radiosensitize NPC .

Noninvasive imaging with hypoxia specific imaging agents is a feasible noninvasive way to measure tumor hypoxia changes following bevacizumab and TPFB, relative to baseline.

Changes in hypoxia resultant from TPFB, based on FMISO PET imaging, may be of predictive value.

Alterations in plasma EBV DNA levels following bevacizumab alone and/ or following TPFB may be predictive of long term outcome for patients with NPC.

2.11 Correlative Studies Background

2.11.1 Hypoxia imaging in NPC

Please see the extended discussion concerning hypoxia, hypoxia imaging, and the potential role of bevacizumab in hypoxia reduction in NPC in the general background section of this protocol. We hypothesize that initial treatment with bevacizumab in addition to TPF may enhance drug delivery and improve hypoxia, which has been documented to exist in NPC patients with Fluorine-18 fluoromisonidazole(FMISO, a hypoxia specific tracer) Positron Emission Tomography (PET) imaging . [45] Reductions in tumor hypoxia have been seen in NPC patients treated with radiation, but we are unaware of any data concerning noninvasive hypoxia reduction imaging with either bevacizumab alone or polychemotherapy in NPC. We will use FMISO PET CT to assess the level of tumor hypoxia pretreatment, after one single treatment with bevacizumab and after completion of induction chemotherapy in order to determine the impact of TPFB on hypoxia modulation in NPC patients prior to chemoradiation. If there is hypoxia reduction prior to radiation, and this correlates with a higher CR rate to the overall chemoradiation regimen used here versus the historical approach in which radiation is used initially, this would support the hypothesis that bevacizumab and chemotherapy prior to radiation in NPC may enhance the overall CR rate in part by diminishing tumor hypoxia, and thereby making the tumor more radiation sensitive.

2.11.2 Serum EBV DNA

Please see the discussion of plasma EBV DNA as a marker for NPC. While there are data to suggest that plasma DNA is a predictor of outcome in patients with NPC, these data are confined to baseline EBV DNA in patients treated with radiation alone or after the completion of radiation in a mixed set of patients. It is unknown whether plasma EBV level alteration after chemotherapy alone is of predictive utility in patients. Similarly, there are no data concerning the effect of bevacizumab on plasma EBV DNA levels . We hypothesize that changes in plasma EBV DNA levels from baseline following bevacizumab treatment and following TPFB treatment may provide additional prognostic information . We propose to collect patient plasma at baseline, after one dose of bevacizumab, after 3 cycles of chemotherapy, and after chemoradiation and evaluate these samples for EBV DNA levels, according to the methodology used in the past by our group. [4]

3. PATIENT SELECTION

3.1 **Eligibility Criteria checklist**

Yes______ NO______3.1.1 Patients must have histologically or cytologically confirmed nasopharyngeal carcinoma, stages II (minimally T2a,N0,M0 or Tany,N1, M0) through IVb. Patients with metastatic (stage IVc) untreated NPC who otherwise meet all eligibility criteria will be enrolled on a separate cohort and evaluated separately. Stage: T_________ N___________ M ___________

Yes______ NO______3.1.2 Patients must have measurable disease, defined as at least one lesion that can be accurately measured in at least one dimension (longest diameter to be recorded) as ≥20 mm with conventional techniques or as ≥10 mm with spiral CT scan. See Measurement of Effect section for details.

Yes______ NO______3.1.3 Prior treatment

Patients may have had diagnostic surgery(s) at the primary site or neck as long as there is still measurable disease present.

Yes______ NO______3.1.4 Age ≥18 years.

Because no dosing or adverse event data are currently available on the use of the TPFB combination) in patients <18 years of age, children are excluded from this study but will be eligible for future pediatric trials.

Yes______ NO______3.1.5 Life expectancy of greater than 3 months.

Yes______ NO______3.1.6 ECOG performance status <2. PS = _________

Yes______ NO______3.1.7 Patients must have normal organ and marrow function as defined below:

-absolute neutrophil count≥1,500/mcL value_______ date_______

-platelets ≥100,000/mcL value_______ date_______

-International normalized ratio (INR) ≤1.5 X institutional ULN value_______ date_______

-Activated partial thromboplastin time (aPTT)≤ 1.5 X institutional ULN value_______ date_______

-total bilirubin ≤1.5 X institutional ULN value_______ date_______

-AST(SGOT)/ALT(SGPT)≤2.5 X institutional ULN value_______ date_____

-creatinine ≤ 1.5 mg/dl value_______ date_______

-creatinine clearance$\geq$55 mL/min/1.73 m^2 for patients with creatinine levels above 1.5 mg/dl value_______ date_______ **Patients with creatinine > grade 1 but less than grade 3 are eligible but should receive carboplatin throughout the protocol instead of cisplatin.**

-Hearing loss < grade 2. **If hearing loss is grade 2, patients are still eligible but should receive carboplatin throughout the protocol instead of cisplatin. ________ hearing grade________ date assessed.**

-Peripheral neuropathy < grade 2. **If peripheral neuropathy is grade 2, patients are still eligible but should receive carboplatin throughout the protocol instead of cisplatin. ________neuropathy grade________ date assessed.**

Cisplatin should be substituted with carboplatin for creatinine > grade 1 , neuropathy > gr2 or hearing loss > grade 2

- Urine protein: creatinine ratio (UPC ratio) < 1.0. Urine protein should be screened by urine analysis for Urine Protein Creatinine (UPC) ratio. For UPC ratio > 1, 24-hour urine protein should be obtained and the level should be <1000 mg for patient enrollment. value_______ date_______

UPC ratio of spot urine is an estimation of the 24 urine protein excretion – a UPC ratio of 1 is roughly equivalent to a 24-hour urine protein of 1 gm. UPC ratio is calculated using one of the following formulas:

- [urine protein]/[urine creatinine] – if both protein and creatinine are reported in mg/dL
- [(urine protein) x0.088]/[urine creatinine] – if urine creatinine is reported in mmol/L

Yes______ NO______3.1.8 The effects of TPFB on the developing human fetus at the recommended therapeutic dose are unknown. For this reason and because these agents as well could be be teratogenic or abortifacient, women of child-bearing potential and men must agree to use adequate contraception (hormonal or barrier method of birth control; abstinence) prior to study entry and for the duration of study participation. Should a woman become pregnant or suspect she is pregnant while participating in this study, she should inform her treating physician immediately. Patient agrees

Yes______ NO______3.1.9 Ability to understand and the willingness to sign a written informed consent document.

3.2 Exclusion Criteria

Yes______ NO______3.2.1 Patients who have had chemotherapy or radiotherapy for nasopharyngeal carcinoma.

Yes______ NO______3.2.2 Patients who have had prior treatment with bevacizumab or other agents specifically targeting VEGF.

Yes______ NO______3.2.3 Patients with known brain metastases should be excluded from this clinical trial because of their poor prognosis and because they often develop progressive neurologic dysfunction that would confound the evaluation of neurologic and other adverse events.

Yes______ NO______3.2.4 History of allergic reactions attributed to compounds of similar chemical or biologic composition to docetaxel, cisplatin, carboplatin, 5-Fluorouracil, bevacizumab or other agents used in the study.

Yes______ NO______3.2.5 Major surgical procedures or traumatic injury within 4 weeks prior to the first treatment . Incisional or excisional surgical biopsies for NPC must have been done > 15 days from the first planned day of treatment. There is no required interval between fine needle aspirate biopsy and treatment initiation PEG tubes may not be placed within 7 days prior to treatment.

Yes______ NO______3.2.6 Patients must not have had gross bleeding from any site within 4 weeks prior to treatment. Clinically insignificant non- arterial mucosal bleeding from the NPC primary site is NOT an exclusion criterion.

Yes______ NO______3.2.7 Pregnant women are excluded from this study. The effects of TPFB on the developing human fetus are unknown. These agents as well could be teratogenic or abortifacient as well. Because there is an unknown but potential risk for adverse events in nursing infants secondary to treatment of the mother with TPFB, breastfeeding should be discontinued if the mother is treated with TPFB. These potential risks may also apply to other agents used in this study.

Yes______ NO______3.2.8 HIV-positive patients on combination antiretroviral therapy are ineligible because of the potential for pharmacokinetic interactions with TPFB. In addition, these patients are at increased risk of lethal infections when treated with marrow-suppressive therapy. Appropriate studies will be undertaken in patients receiving combination antiretroviral therapy when indicated.

Yes______ NO______3.2.9 Uncontrolled intercurrent illness including, but not limited to, ongoing or active infection, symptomatic congestive heart failure, unstable angina pectoris, cardiac arrhythmia, or psychiatric illness/social situations that would limit compliance with study requirements.

Yes______ NO______3.2.10 Patients with clinically significant cardiovascular disease are excluded

Yes______ NO_____ Inadequately controlled HTN (SBP > 160 mmHg and/or DBP > 90 mmHg despite antihypertensive medication)

Yes______ NO______ History of CVA within 6 months

Yes______ NO______ Myocardial infarction or unstable angina within 6 months

Yes______ NO______ New York heart association grade II or greater congestive heart failure,

Yes______ NO______Serious and inadequately controlled cardiac arrhythmia

Yes______ NO______ Significant vascular disease (e.g. aortic aneurysm, history of aortic dissection)

Yes______ NO______Clinically significant peripheral vascular disease

3.2.11 Patients on full-dose anticoagulants (warfarin or heparin) are eligible provided that both of the following criteria are met:

Yes______ NO______ 3.2.11.1 The patient has no active bleeding or pathological condition that carries a high risk of bleeding.

Yes______ NO______ 3.2.11.2 Patient is on stable dose of anticoagulation (if on warfarin, INR should be stable in the therapeutic range).

3.3 **Inclusion of Women and Minorities**

Both men and women and members of all races and ethnic groups are eligible for this trial.

4. REGISTRATION PROCEDURES

Appropriate forms for the study (e.g., Eligibility Screening Worksheet, Registration Form)will be developed and included with the protocol. These forms must be used by all participating institutions for data submission.

4.1 General Guidelines

Eligible patients will be entered on study centrally at the Stanford Cancer Center_by the Study Coordinator. All sites should call the Study Coordinator _ Ruth Lira at 650 723 1367 or email at rlira@stanford.edu to verify agent availability. See details below. Eligibility must be confirmed by the study coordinator prior to treatment. The required forms can be found in Appendix F

Following registration, patients should begin protocol treatment within 1 week. Issues that would cause treatment delays should be discussed with the Principal Investigator. If a patient does not receive protocol therapy following registration, the patient's registration on the study may be canceled. The Study Coordinator should be notified of cancellations as soon as possible.

Except in very unusual circumstances, each participating institution will order DCTD-supplied agents directly from CTEP. Agents may be ordered by a participating site only after the initial IRB approval for the site has been forwarded by the Coordinating Center to the CTEP PIO (PIO@ctep.nci.nih.gov) (except for Group studies).

4.2 Registration Process

To register a patient, the following documents should be completed by the research nurse or data manager and faxed 650 498 5800 ATTN : Ruth Lira or e-mailed *rlira@stanford.edu* to the Study Coordinator:

- Copy of required laboratory tests
- Signed patient consent form
- HIPAA authorization form(embedded in consent form)
- Eligibility Screening Worksheet.

The research nurse or data manager at the participating site will then call 650 723 1367 or e-mail *rlira@stanford.edu* to verify eligibility. To complete the registration process, the Coordinator will

- assign a patient study number
- register the patient on the study
- fax or e-mail the patient study number and dose to the participating site
- Call the research nurse or data manager at the participating site and verbally confirm registration.

5. TREATMENT PLAN

5.1 Prophylactic gastrostomy feeding tubes.

Investigators are **STRONGLY ENCOURAGED** to have prophylactic gastrostomy feeding tubes placed in patient **prior to initiation of treatment.** Chemoradiation for NPC patients is known to be associated with a high rate of severe locoregional toxicity including severe mucositis, skin breakdown, nausea, and presence of thick copious tenacious secretions, often complicated by oropharyngeal candidiasis and superficial ulceration and superficial bleeding oral alimentation and hydration extremely difficult. Because the bleeding and wound healing risks associated with bevacizumab use in this trial would complicate the placement of gastrostomy feeding tubes once treatment is underway, the practice of delayed placement of feeding tubes could unnecessarily

interrupt treatment.

5.2 Agent Administration

Treatment will usually be administered on an outpatient basis. Reported adverse events and potential risks for the chemotherapeutic agents are described in the Agent Adverse Events section of each agent . Appropriate dose modifications for chemotherapeutic agents and _are described in the **DOSING DELAYS/DOSE MODIFICATIONS section below.** No investigational or commercial anti-cancer agents or therapies other than those described below may be administered with the intent to treat the patient's malignancy.

5.2.1

Induction TPFB treatment (note the first day of each cycle =day "1"					
Agent	***Premedications; Precautions***	***Dose***	***Route***	***Schedule***	***Cycle Length***
Bevacizumab	*Cycles 2 and beyond: dexamethasone 12 mg PO or IV prior to bevacizumab on day 1. Antiemetics per text below*	*15 mg/kg in 100 cc NS*	*IV over 90 minutes. Subsequent infusion times can be modified according to the FDA label for Avastin*	*Cycle 1: Day minus 7.*<br><br>*All subsequent cycles: day 1*	*3 weeks(21 days)*
Docetaxel	*Dexamethasone 8 mg PO the evening of day 0(patient to have taken at home)*	*75 mg/m^2 in 250 cc NS*	*IV over 60 minutes immediately after bevacizumab*	*Day 1, all cycles*	
Cisplatin (if substituting carboplatin, see # for directions)	*Antiemetics and Hydration per text below*	*75 mg/m2*	*IV over 1-3 hours.*	*Day 1, all cycles*	
*Carboplatin **(only if substituting for cisplatin***	*Antiemetics and Hydration per text below*	*AUC 6 using the below formula**	*IV over 30 minutes*	*Day 1 all cycles*	
5-Fluorouracil	*Antiemetics and Hydration per text below*	*750 mg/m2/d x 5 doses*	*IV continuous infusion over 24 hours daily*	*Days 1,2,3,4,5 May start 5- FU infusion CONCURRENTLY with Cisplatin infusion start.*	
Ciprofloxacin 500 mg		*500 mg BID*	*PO or per G tube*	*Days 6-15*	

#Cisplatin should be substituted with carboplatin for creatinine > grade 1 , neuropathy > gr2 or hearing loss > grade 2.

* Carboplatin dose = AUC * (GFR+25) Where GFR estimate = (140 - age) * weight in

kg / (72 * serum creatinine). Multiply GFR estimate by 0.85 for females.

5.2.2 Premedications , antiemetics and intravenous fluid support for TPFBduring induction chemotherapy

Premedications for TPFB

On day 1

Prior to bevacizumab (prior to docetaxel for cycle 1):

Dexamethasone 12 mg PO or IV

Famotidine 20 mg IV or PO or therapeutic equivalent. DO NOT USE CIMETIDINE

Diphenhydramine 25-50 mg PO or IV

Antiemetics for TPFB

On day 1:

Granisetron 2 mg PO or therapeutic equivalent

Aprepitant 125 mg PO

On days 2 and 3:

Dexamethasone 8 mg PO or IV

Aprepitant 80 mg PO

On day 4:

Dexamethasone 8 mg PO or IV

On any day of the cycle for breakthrough nausea, additional granisetron, metochlopramide, prochlorperazine or lorazepam may be used per local routine.

Intravenous fluid support for TPFB

Local institutional practices for IV fluid support may be used as long as all patients receive 3 liters of IVF with corresponding adequate urine output on day 1 and 1-2 liters of IV fluid as needed on days 2 and 3.

IVF and diuresis recommendations: Patient shall in aggregate have received 1 L IVF prior to cisplatin, including fluids in which other agents have been administered. Our practice is to give, in addition to IVF received with other agents, 500 -1000 mL NS over 2 hours, administered concurrently with bevacizumab and docetaxel.

Give 12.5mg mannitol IVP immediately prior to cisplatin.

Give concurrently with cisplatin 500 cc NSS containing 5 meq KCl, 1gram Magnesium Sulfate and 25 grams Mannitol .

Give post cisplatin : 1 liter NSS containing 10 mEq KCL/liter,1 gm Magnesium Sulfate/ l at 500 ml/hr .

On days 2 and 3, give 1-2 liters NSS IV PRN for poor oral fluid intake

5.2.3 Growth factor support:

No primary prophylactic administration of G-CSF or GM-CSF (for first TPFB chemotherapy cycle) is permitted.

GM-CSF or G-CSF will be administered (dosing as above or as per treating institution standard) prophylactically during the second and/or third cycles if the following conditions are met during the first or second cycle:

- Patients with a prior episode of febrile neutropenia or neutropenic infection
- Delayed recovery of absolute neutrophil count at day 21
- Grade 4 neutropenia (ANC $< 0.5 \times 10^9$/L), which persists for ≥ 7 days

Route: subcutaneously

Schedule: starting 24 hours after the completion of 5-fluorouracil infusion. GM-CSF or G-CSF will be administered once daily for 10 days or until ANC $> 1.5 \times 10^9$/L for 2 consecutive measurements, whichever occurs earlier. G-CSF as Neulasta also may be given per treating institution standard.

Once indicated, GM-CSF or G-CSF will be given in all subsequent TPFB cycles.

5.2.4 Chemotherapy concurrent with radiation:

All patients will receive chemoradiotherapy after the end of TPFB with a minimum interval of 3 weeks and no later than 6 weeks after start of the last cycle (day 22 to 42 of last cycle).

Patients must fulfill the following criteria for chemoradiation:

Mucositis < grade 2

ANC ≥ 1500/ microliter

PLT ≥100, 000/ microliter

Hemoglobin ≥ 10 g/dL or hematocrit ≥ 30%

Creatinine ≤ 1.5 mg/dl OR creatinine clearance≥55 mL/min/1.73 m^2 for patients with creatinine levels above 1.5 mg/dl. **Cisplatin should be substituted with carboplatin for creatinine > grade 1 , neuropathy > gr2 or hearing loss > grade 2**

Chemotherapy concurrent with radiation				
Agent	***Premedications; Precautions***	***Dose***	***Route***	***Schedule***
Bevacizumab	*Premedications per standard of care PRN in case of prior bevacizumab hypersensitivity*	*15 mg/kg in 100 cc NS*	*IV over 90 minutes or less per FDA package insert guidelines*	*Weeks 1, 3, and 6 of radiation*
Cisplatin (if substituting carboplatin, see text for directions)	*Antiemetics and Hydration per text below*	*40mg/m2*	*IV over 1 hour*	*Weekly during radiation for a total of 6 doses*
Carboplatin ***(only if substituting for cisplatin per the above guidelines)***	*Antiemetics and Hydration per text below*	*AUC 1.5 using the below formula**	*IV over 30 minutes*	*Weekly during radiation for a total of 6 doses*
Radiation therapy	three dimensional conformal or intensity modulated radiation therapy to 70 Gy to the gross target volume + margins in 33 fractions. See section 5.3 for details			

* Carboplatin dose = AUC * (GFR+25) Where GFR estimate = (140 - age) * weight in kg / (72 * serum creatinine). Multiply GFR estimate by 0.85 for females.

5.2.5 Premedications , antiemetics and intravenous fluid support for TPFB during concurrent chemoradiation

Antiemetics for ***Concurrent CISPLATIN***

On day 1:

Granisetron 2 mg PO or therapeutic equivalent

Aprepitant 125 mg PO

Dexamethasone 12 mg PO or IV

On days 2 and 3:

Dexamethasone 8 mg PO or IV

Aprepitant 80 mg PO

On any day for breakthrough nausea, additional granisetron, metochlopramide, prochlorperazine or lorazepam may be used per local routine.

Intravenous fluid support ***Concurrent CISPLATIN***

Local institutional practices for IV fluid support may be used as long as all patients receive 2 liters of IVF with corresponding adequate urine output on day 1 and 1-2 liters of IV fluid as needed on days 2 and 3.

Antiemetics for ***concurrent CARBOPLATIN***

On day 1:

Granisetron 2 mg PO or therapeutic equivalent

Dexamethasone 8 mg PO or IV.

On any day for breakthrough nausea, additional granisetron, metochlopramide, prochlorperazine or lorazepam may be used per local routine

Intravenous fluid support ***Concurrent CARBOPLATIN***

None needed

5.3 Radiation therapy treatment plan:

5.3.1 Concurrent bevacizumab, cisplatin, and radiation:

Radiation Therapy: 70 Gy at 2.0-2.12 Gy/fraction in 6.5-7 weeks delivered with either 3-dimensional conformal radiotherapy (3DCRT) or intensity modulated radiotherapy (IMRT).

5.3.2 Radiation Therapy (See also Appendix E– Radiation Quality Assurance form)

Allowable treatment approaches include a 3DCRT approach or an IMRT.

Dose specification:

Two different RT dose prescriptions are allowed:

5.3.2.1. Integrated dose prescription

PTV_{70} (planning target volume 70): [GTV (gross target volume) + margin]: will receive 70 Gy at 2.12 Gy/fraction for 33 fractions.

$PTV_{high\ risk}$ [$CTV_{high\ risk}$ (clinical target volume high risk) + margin]: the areas of high risk, sub-clinical disease will receive between 56-59.4 Gy (at the discretion of the treating physician) at 1.7-1.8 Gy/fraction for 33 fractions.

$PTV_{low\ risk}$ ($CTV_{low\ risk}$ + margin): The area of low risk, subclinical disease, which is predominantly the uninvolved low necks, will receive 54 Gy at 1.64 Gy/fraction. Alternatively, the uninvolved low neck can be treated with a conventional AP or APPA supraclavicular field to a total dose of 46-50 Gy at 2Gy fraction for 23-25 fractions. The dose is prescribed to a depth of 3 cm from the anterior surface for the AP field and to the midplane for the APPA field. The junction between the IMRT or 3DCRT fields and the low-neck fields will be dependent on the institutional IMRT techniques; however, each institution is required to record the dosimetric details at the match-line to ensure dose homogeneity and to prevent overdosing of the spinal cord.

5.3.2.2. Sequential dose prescription:

PTV_{70} (planning target volume 70): [GTV (gross target volume) + margin]: will receive 70 Gy at 2.0 Gy/fraction for 35 fractions.

$PTV_{high\ risk}$ [$CTV_{high\ risk}$ (clinical target volume high risk) + margin] (**optional**): the areas of high risk, sub-clinical disease will receive 60 Gy at 2 Gy/fraction for 30 fractions.

$PTV_{low\ risk}$ ($CTV_{low\ risk}$ + margin): The area of low risk, subclinical disease, which is predominantly the uninvolved low necks, will receive 50 Gy at 2 Gy/fraction in 25 fractions. Alternatively, the uninvolved low neck can be treated with a conventional AP or APPA supraclavicular field to a total dose of 46-50 Gy at 2Gy fraction for 23-25 fractions. The dose is prescribed to a depth of 3 cm from the anterior surface for the AP field and to the midplane for the APPA field. The junction between the IMRT or 3DCRT fields and the low-neck fields will be dependent on the institutional IMRT techniques; however, each institution is required to record the dosimetric details at the match-line to ensure dose homogeneity and to prevent overdosing of the spinal cord.

5.3.2.3 Dose Compliance

The reported dose for each PTV should include the prescribed dose, maximal point dose, mean dose, the % of PTV that receive $\geq$ 110%, $\geq$ 115% and $\leq$ 93% of the prescribed dose.

All plans should be normalized so that $\geq$ 95% of the PTV_{70} receives the prescribed dose. In addition, no more than 20% of the PTV_{70} will receive $\geq$ 110 % and no more than 5% will receive $\geq$ 115%

RT will be given as once daily fraction. The first RT treatment should begin on Monday, Tuesday or Wednesday.

5.3.2.4 Technical factors

External beam equipment and beam delivery methods

Megavoltage equipments capable of delivering 3DCRT or IMRT (either static or dynamic) are required.

Treatment planning, imaging and localization requirement

The immobilization device should include at least the head and neck. It is strongly encouraged that the participation centers also utilize shoulder immobilization especially when comprehensive nodal IMRT is utilized.

Treatment planning CT scan will be required to delineate the GTV, CTV and PTV. Other imaging studies such as MRI and PET-CT scans can aid in volume delineation. The treatment planning CT scan should be acquired with the patient immobilized in the same treatment position. All tissue irradiated should be included in the treatment planning CT scan, which should be $\leq$ 3 mm slice thickness through the regions containing the GTV. Thicker slices (up to 5 mm) may be used for region above or below the GTV; however, thicker slices may compromise the image quality of the digitally reconstructed radiographs (DRR)

Treatment planning/target volumes

The definition of the target volumes should conform to the 1993 ICRU report #50;

Gross target volume (GTV): All known gross disease determined from clinical (including endoscopic) and imaging findings. Grossly involved nodes are defined as any lymph node > 1 cm on CT or MRI in the minimal cross-sectional diameter, any nodes with increased metabolic uptake on FDG PET scan, any node with central necrosis and/or radiographic evidence of extracapsular extension regardless of size.

Clinical target volume (CTV):

CTV_{70}: For grossly positive node, a margin of 5 mm should be added circumferentially to account for microscopic extracapsular extension

$CTV_{high\ risk}$: should include all regions deemed to be at high risk for microscopic disease, all potential routes of spread, and the high risk nodal regions.

$CTV_{low\ risk}$: nodal regions at low risk for microscopic involvement. This usually constitutes the clinically and radiographically low-neck nodes.

Planning target volume: A margin should be used to account for intrafraction and interfraction set up variability. The average recommended PTV margin is 5 mm; however, it will depend on the accuracy of treatment set up and immobilization at each individual treatment site.

5.3.2.5 Treatment Plan

Treatment plan will be based on the analysis of the volumetric dose, including dose-volume histogram (DVH) analyses of the PTVs and critical normal structures. A 3D or an "inverse" planning using computerized optimization should be used. The treatment aim will be the delivery of radiation to the PTVs and the exclusion of non-involved tissues. **HETEROGENEITY CORRECTION SHOULD BE USED**.

5.3.2.6 Critical structures

Surrounding critical normal structures, including the brainstem, temporal lobes (if the tumor is near the skull base), spinal cords, optic nerves, eyes, optic chiasm, parotid glands, the inner and middle ears (if the targets are near by), oral cavity, mandible and glottic larynx should be outlined. If there is grossly involved tumor in the low neck, then the brachial plexus should also be delineated.

Unspecified tissues, defined as the tissues within the skin, subtracted all target volumes and delineated normal tissues, should also be taken into account of the treatment planning and evaluation. No more than 5% of the unspecified tissue can receive > 70 Gy and no more than 1% or 1 cc of this tissue can receive >= 77 Gy. **Participants are strongly encouraged to remain within these limits.**

Dose constraints for certain normal tissues are shown in the following table.

Table 1: Required critical structure dose constraints

**Structure**	**Maximal dose (Gy)**
Brainstem	54
Spinal cord	45
Optic nerves	54
Optic chiasms	54
Eyes	50
Mandible	70
Brachial plexus	66

Table 2: Suggested normal structure dose constraints

**Structure**	**Mean dose (Gy)**
Parotid	< 26 for 1 gland or < 30 for 50% of 1 gland or < 20 for 20 cc volume of both glands
Oral cavity	< 40 if tumors outside the oral cavity
Inner/middle ear	< 45 or $\leq$ 5% volume receives > 55 Gy
Glottic larynx	< 50 for tumor outside the larynx and hypopharynx

Esophagus/post cricoid pharynx	< 50 for tumor outside the larynx and hypopharynx

5.3.2.7 Documentation requirements:

Weekly verification of orthogonal films through the treatment isocenter is required. If the IMRT or 3DCRT fields are matched to an AP supraclavicular field, then the supraclavicular field should also be included during weekly portal verification. **Appendix E is the radiation quality assurance form that should be submitted at the completion of radiation therapy**.

5.3.2.8 Radiation adverse events and allowed interruption

Radiation adverse events will be graded as per CTCAE v3.0. RT interruption may be necessary due to severe acute RT - related reactions such as severe skin or mucosal reaction or any other acute complications. Interruptions will be left at the discretion of the treating physicians but strongly discouraged. The cause of interruption should be recorded.

Placement of feeding gastrostomy tube may be necessary for nutritional support in this frail population. The date and reason for placement of a feeding gastrostomy tube (either prophylactic in preparation for RT or for active nutritional support due to significant weight loss before or during RT) should also be recorded. The duration of feeding tube dependence after completion of RT should also be recorded.

5.4 **Duration of Follow Up**

Patients will be followed for a minimum of 1 year after removal from study or until death, whichever occurs first. Patients removed from study for unacceptable adverse events will be followed until resolution or stabilization of the adverse event.

Patients will be seen quarterly in follow- up the first year after completion of radiation, and subsequently per local standards of care.

6. DOSING DELAYS/DOSE MODIFICATIONS

Doses will be modified in case of severe hematological and/or non-hematological toxicities. Dose adjustments are to be made according to the CTCAE v3 system showing the greatest degree of toxicity. Toxicities will be graded using the CTCAE v3 criteria.

6.1 Bevacizumab Dose modifications:

There will be no dose reduction for bevacizumab.

Treatment should be interrupted or discontinued for certain adverse events, as described below. If bevacizumab is interrupted for ANY reason for > 3 weeks, the patient should discontinue bevacizumab therapy on protocol.

Treatment Modification for Bevacizumab-Related Adverse Events

Event	CTCAE.v3.0 Grade	Action to be Taken
**Allergic reactions, or**<br>**Acute infusional reactions/ cytokine release syndrome**	Grade 1-3	**For patients with Grade 3 reactions**, bevacizumab infusion should be stopped and not restarted on the same day. At the physicians' discretion, bevacizumab may be permanently discontinued or re-instituted with premeds and at a rate of 90±15 min.
	Grade 4	Discontinue bevacizumab
**Arterial Thrombosis**<br>- Cardiac ischemia/ infraction<br>- CNS ischemia (TIA, CVA)<br>- any peripheral or visceral arterial ischemia/thrombosis	Grade 2 (if new or worsened since bevacizumab therapy)	Discontinue bevacizumab.
	Grade 3-4	Discontinue bevacizumab
**New Venous Thrombosis**		
	Grade 3<br>OR<br>asymptomatic Grade 4	▪ Hold bevacizumab treatment. If the planned duration of full-dose anticoagulation is <2weeks, bevacizumab should be held until the full-dose anticoagulation period is over.<br>▪ If the planned duration of full-dose anticoagulation is >2 weeks, bevacizumab may be resumed during full-dose anticoagulation **IF** <u>all</u> of the criteria below are met:<br>− The subject must not have pathological conditions that carry high risk of bleeding (e.g. tumor involving major vessels or other conditions)<br>− The subject must not have had hemorrhagic events while on study<br>− The subject must on stable dose of heparin or have an in-range INR (usually 2-3) on a stable dose of warfarin prior to restarting bevacizumab.<br><br>▪ If thromboemboli worsen/recur upon resumption of study therapy, discontinue bevacizumab
	Grade 4 (symptomatic)	Discontinue bevacizumab

Event	CTCAE.v3.0 Grade	Action to be Taken
**Hypertension**	[Treat with anti-hypertensive medication as needed. The goal of BP control should be consistent with general medical practice]	
	Grade 1	Consider increased BP monitoring
	Grade 2 asymptomatic **but** diastolic BP < 100 mmHg	Begin anti-hypertensive therapy and continue bevacizumab
	-Grade 2-3 Symptomatic **OR** -Diastolic BP > 100 mmHg	• Hold bevacizumab should until symptoms resolve **AND** BP < 160/90mmHg*
	Grade 4	Discontinue bevacizumab.
**Congestive Heart Failure**	Grade 3 (symptomatic)	Discontinue bevacizumab
	Grade 4	Discontinue bevacizumab
**Proteinuria**	[Proteinuria should be monitored by urine analysis for urine protein creatinine (UPC) ratio prior to every other dose of bevacizumab]	
	UPC ratio < 3.5	Continue bevacizumab.
	UPC ratio ≥ 3.5	Hold bevacizumab until it UPC recovers to < 3.5.
	Grade 4 or nephrotic syndrome	Discontinue bevacizumab.
**Hemorrhage (CNS or pulmonary)**	Grade 2-4	• Discontinue bevacizumab
**Hemorrhage (non-CNS; non-pulmonary)**	Grade 3	• Patients receiving full-dose anticoagulation should discontinue bevacizumab.<br>• For patients not on full-dose anticoagulation, hold bevacizumab until ALL of the following criteria are met:<br>- the bleeding has resolved and Hb is stable<br>- there is no bleeding diathesis that would increase the risk of therapy<br>- there is no anatomic or pathologic condition that could increase the risk of hemorrhage recurrence.<br>• Patients who experience recurrence of grade 3 hemorrhage should discontinue study therapy.
	Grade 4	Discontinue bevacizumab

Event	CTCAE.v3.0 Grade	Action to be Taken
**RPLS** (Reversible Posterior Leukoencephalopathy syndrome **or PRES** (Posterior Reversible Encephalopathy Syndrome)		• Discontinue bevacizumab upon diagnosis of RPLS.
**Wound dehiscence** requiring medical or surgical intervention		• Discontinue bevacizumab
**Perforation (GI, or any other organ)**		Discontinue bevacizumab
**Fistula (GI, pulmonary or any other organ)**		Discontinue bevacizumab
**Bowel obstruction**	G2 requiring medical intervention	• Hold bevacizumab until complete resolution
	G3-4	• Hold bevacizumab until complete resolution<br>• If surgery is required, patient may restart bevacizumab after full recovery from surgery, and at investigator's discretion
**Other Unspecified bevacizumab-related AEs** (except controlled nausea/vomiting).	Grade 3	• Hold bevacizumab until symptoms resolve to ≤ grade 1
	Grade 4	• Discontinue bevacizumab<br>• **Upon consultation with the study chair,** resumption of bevacizumab may be considered if a patient is benefiting from therapy, and the G4 toxicity is transient, has recovered to ≤ grade 1 and unlikely to recur with retreatment.

6.2 DOCETAXEL dose modifications

Febrile Neutropenia or Documented Neutropenic Infection

Adverse event	Action to be taken for subsequent cycles
• Febrile neutropenia<br>• Documented infection	The first episode of febrile neutropenia or documented grade 3 /4 neutropenia with documented infection will result in the addition of GM-CSF or G-CSF to all subsequent cycles .<br>If there is a second episode, the patient will remain on Ciprofloxacin and GM-CSF or G-CSF and additionally, during the subsequent cycles, Docetaxel dose will be reduced from 75 to 60 mg/m²

Action Taken Following Results of CBC Counts On first day of each TPFB cycle

ANC ($x10^9/L$)	Action to be taken
≥ 1.5 (grade 1)	Treat on time

< 1.5 (GRADE 2 OR HIGHER)	1. Delay TPFB 1 week and repeat complete blood count .<br>2. If ANC > 1.5 x 10^9/L, then proceed with full dose chemotherapy<br>3. If ANC < 1.5 x 10^9/L, then consider addition of G-CSF or GM-CSF for 7 days<br>• On day 35, perform complete blood count with differential<br>• Proceed with full dose chemotherapy if ANC > 1.5<br>• And consider use of GM-CSF or G-CSF in remaining cycles<br>4. If there is no recovery by day 35, (ANC < 1.5 x 10^9/L), the patient will go off TPFB chemotherapy
Thrombocytopenia	Action to be taken
Plt <LLN – 75,000/mm3 (grade 1)	Treat on time
Plt < 75,000 (grade 2 -4)	1. Delay TPFB 1 week and repeat complete blood count .<br>2. If plt ≥ 100,000, then proceed with full dose chemotherapy<br>3. If plt 75,000-<100,000, dose reduction of docetaxel from 75 to 60 mg/m^2 for all subsequent cycles of TPFB.<br>4. if plt < 75,000, patient will go off TPFB chemotherapy<br><br>If patient has recurrent thrombocytopenia after docetaxel reduction to 60 mg/m2 without recovery to 75,000 by day 28 of subsequent cycles, patient will go off TPFB therapy

Action Taken for other Docetaxel AEs

Stomatitis

If stomatitis is present on day 1 of any cycle, treatment should be withheld until stomatitis has resolved.

If Grade 3/4 stomatitis occurs at any time, the dose of Docetaxel should be reduced for subsequent cycles, Docetaxel dose will be reduced from 75 to 60 mg/m^2

Peripheral Neuropathy

Docetaxel dose will be reduced from 75 to 60 mg/m^2 for Grade 2 neuropathies without treatment delay.

Treatment should be discontinued for Grade 3/4 neuropathies.

Dermatological/ Skin

Grade 0,1 and 2: no change

Grade 3: Delay until < grade 1 and retreat with a dose reduction of Docetaxel from 75 to 60 mg/m². If no recovery to < grade 1 within 2 weeks delay, patient will go off protocol therapy.

Grade 4: The patient will go off chemotherapy.

Nausea and/or vomiting

Prophylactic antiemetic regimen with 5-HT3 antagonist should be administered from the first cycle. In addition, the corticosteroids used during 3 days for the prophylaxis of fluid retention should also reduce the incidence and severity of emesis.

Patients with nausea and vomiting despite these measures may be treated with another antiemetic regimen (i.e. high dose metochlopramide) as appropriate.

Bilirubin and Impaired liver function:

In the event that bilirubin levels are abnormal during study, the next cycle will be delayed by a maximum of 2 weeks. If no recovery, the patient should be taken off chemotherapy.

In the event that AST and/or ALT and/or alkaline phosphatase levels are abnormal in the absence of progressive disease, the following dose modifications will apply:

Table :Dose Modifications for docetaxel for Abnormal Liver Function

	**AST or ALT:**			
**ALK PHOS #:**	**≤ ULN**	**>1x but ≤1.5x**	**>1.5x but ≤5x**	**>5x ULN**
**≤ ULN**	Full Dose	Full Dose	Full Dose	Hold*
**>1x but ≤ 2.5x**	Full Dose	Full Dose	Reduce Dose to 60 mg/m2	Hold*
**>2.5x but ≤ 5x**	Full Dose	Reduce Dose to 60 mg/m2	Hold*	Hold*
**>5x ULN**	Hold*	Hold*	Hold*	Hold*

*Hold until recovered, maximum 2 weeks, then re-treat at a reduced dose. "Recovered" is defined as meeting the study baseline eligibility criteria.

Bilirubin: Docetaxel should not be administered to patients with serum total bilirubin >ULN. If serum total bilirubin is >ULN on treatment day, hold Docetaxel until serum total bilirubin is ≤ ULN (maximum 2 weeks), then re-treat at a reduced dose.

**Reduced doses of Docetaxel will be at 60mg/m^2. After Docetaxel is dose is reduced, there will be no re-escalation. There will be only one dose reduction.

If alkaline phosphatase is clinically related to local bone erosion, for purposes of docetaxel dose reductions, consider alk phos < ULN, i.e. dose adjust based on other clinical and lab parameters.

<u>6.3 CISPLATIN and CARBOPLATIN dose modifications:</u>

Cisplatin dose reductions during TPFB for hematologic adverse events:

There will be no planned CDDP dose reductions for ANC or platelet AEs. See Docetaxel section for dose reduction and TPFB delay and discontinuance parameters for ANC and platelet AEs. Note that after one reduction in docetaxel for hematological AEs, high grade persistent or recurrent AEs will result in discontinuance of TPFB per the algorithm outlined for docetaxel dose modification.

isplatin dose reductions during TPFB for non- hematologic adverse events:

CDDP dose levels during TPFB	
-1	Starting dose
60 mg/m2	75 mg/m2

Peripheral Neuropathy

Grade 0,1: no change

Grade > 2: Carboplatin may be substituted for Cisplatin

Ototoxicity

Cisplatin is known to cause high frequency hearing loss. If grade 1 or 2 hearing loss occurs, the risk of additional hearing loss versus the potential benefit of continuing Cisplatin chemotherapy should be made. Grade 3 and 4 hearing loss is an indication to discontinue the drug. In case of grade 3 or 4 ototoxicity, Carboplatin may be used to replace Cisplatin .

Creatinine

Grade 1 (creatinine <1.5): no CDDP dose change

Grade 2 (creatinine > 1.5-3):

first incidence decrease one dose level, consider change to carboplatin.

second incidence: switch to carboplatin.

Grade 3 -4 (creatinine >3): discontinue CDDP, switch to carboplatin.

All other non- hematological AEs attributable to CDDP:

Grade 1-2 : no dose change of CDDP

Grade 3-4:Hold TPFB up to one week for resolution of AEs to grade 2 or less, then re-treat with one level dose reduction of CDDP. If AEs not resolved to grade 2 after 1 week, discontinue TPFB treatment

Carboplatin dose reductions during TPFB

Hematological AEs during TPFB :

There will be no planned Carboplatin dose reductions for ANC or platelet AEs. See Docetaxel section for dose reduction and TPFB delay and discontinuance parameters for ANC and platelet AEs. Note that after one reduction in docetaxel for hematological AEs, high grade persistent or recurrent AEs will result in discontinuance of TPFB per the algorithm outlined for docetaxel dose modification.

All other non- hematological AEs attributable to carboplatin:

Grade 1-2 : No dose change of carboplatin or TPFB delay.

Grade 3-4: Hold TPFB up to one week for resolution of AEs to grade 2 or less, then re-treat with one level dose reduction. If AEs not resolved to grade 2 after 1 week, discontinue TPFB treatment.

Cisplatin and carboplatin dose reductions during concurrent radiation for hematological toxicities

CDDP dose levels during radiation		
-2	-1	Starting dose
25 mg/m2/week	30 mg/m2/week	40mg/m2/week

Carboplatin dose levels during radiation		
-2	-1	Starting dose
AUC 0.8/week	AUC 1.1/week	AUC 1.5 /week

CDDP or Carboplatin must not be administered concurrently with radiation until the ANC ≥ 1,000 and platelets are ≥ 100,000. If not, delay one week. If the patient still has not recovered , continue to hold on a week by week basis until the above criteria are met, then resume dosing according to the below table.

ANC		Plt count	Dose reduction/ delay
Greater or = to 1500	and	Greater or = 75,000	No change in dose
1000-1499	or	50,000-74,999	Decrease by one dose level
Less than 1000	or	Less than 50,000	Hold until ANC>1000 and plt> 75,000 and decrease by one dose level.

There will be no reduction below dose level -2. If a patient is already at dose level -2 and experiences AEs as defined above, discontinue CDDP or carboplatin.

Cisplatin or carboplatin dose reductions during concurrent radiation for non-hematological toxicities:

Concurrent platinum and radiation for patients with nasopharyngeal cancer is known to be associated with a high rate of severe locoregional toxicity including severe mucositis, skin breakdown, nausea, and presence of thick copious tenacious secretions, often complicated by oropharyngeal candidiasis and superficial ulceration and superficial bleeding. Patients commonly are not able to adequately aliment or hydrate themselves orally during chemoradiation and for several weeks afterwards. Every effort should be made to manage patient symptomatically using IV hydration, and clinicians are STRONGLY ENCOURAGED to have prophylactic gastrostomy feeding tubes placed prior to initiation of treatment. Severe mucositis and skin breakdown in the radiation field based on ulceration and superficial bleeding should not be considered inherently dose limiting.

Radiation associated mucositis, stomatitis or dermatitis:

Grade 4: hold CDDP or carboplatin until resolution to grade 3 then dose reduce by one dose level. There will be no dose reductions below dose level minus 2. In the case that a patient is being treated at dose level minus 2, once AEs are grade 3 or less, the patient should be treated again at dose level minus 2.

Peripheral Neuropathy

Grade 0,1: no change

Grade > 2: Carboplatin may be substituted for Cisplatin Should a new grade > 2 peripheral neuropathy develop on carboplatin, hold carboplatin until resolution to grade 2 then dose reduce 1 level.

Ototoxicity

Cisplatin is known to cause high frequency hearing loss. If grade 1 or 2 hearing loss occurs, the risk of additional hearing loss versus the potential benefit of continuing cisplatin chemotherapy should be made. Grade 3 and 4 hearing loss is an indication to discontinue the drug. In case of grade 3 or 4 ototoxicity, carboplatin may be used to replace cisplatin. Should new grade > 2 ototoxicity develop on carboplatin, hold carboplatin until resolution to grade 2 then dose reduce 1 level.

Creatinine

Grade 1 (creatinine <1.5): no CDDP dose change

Grade 2 (creatinine > 1.5-3):

First incidence decrease one dose level, consider change to carboplatin.

Second incidence: switch to carboplatin.

Grade 3 -4 (creatinine >3): discontinue CDDP, switch to carboplatin. Should a new grade > 2 creatinine develop on carboplatin, hold carboplatin until resolution to grade 2 then dose reduce 1 level.

Non- hematological AEs attributable to CDDP or carboplatin excluding AEs discussed above:

Grade 1-2 : No dose change of CDDP or carboplatin.

Grade 3-4: Hold CDDP or carboplatin until resolution of AEs to grade 2 or less, then dose reduce by one dose level. There will be no dose reductions below dose level minus 2. In the case that a patient is being treated at dose level minus 2, once AEs resolve to grade 2 or less, the patient should be treated again at dose level minus 2.

6.4 5- Fluorouracil (5-FU) dose modifications during TPFB

5-FU dose levels during TPFB		
-2	-1	Starting dose
480 mg/m2 IVCI/d x 5 d	600 mg/m2 IVCI/d x 5 d	750 mg/m2 IVCI/d x 5 d

Hematological AEs during TPFB:

There will be no planned 5-FU dose reductions for ANC or platelet AEs. See Docetaxel section for dose reduction and TPFB delay and discontinuance parameters for ANC and platelet AEs. Note that after one reduction in docetaxel for hematological AEs, high grade persistent or recurrent AEs will result in discontinuance of TPFB per the algorithm outlined for docetaxel dose modification.

Non- Hematological AEs during TPFB:

Mucositis, stomatitis or dermatitis

Grade 3 lasting more than 96 hours or grade 4 **:** Dose reduce one level.

Diarrhea

In the case of severe diarrhea, octreotide is recommended. If the patient has a significant diarrhea occurrence again (> 3 loose stools/24 hr), the patient should be treated prophylactically in the subsequent cycles with 2 tablets of loperamide or diphenoxylate in addition to 1 or 2 tablets after each loose stool. The maximum daily dose of Loperamide is 16mg and Diphenoxylate is 20mg/day.

39

Grade 4 diarrhea, or grade 3 diarrhea lasting > 7 days despite the prophylactic treatment: dose reduce one level.

All other non- hematological AEs attributable to 5-FU:

Grade 1-2 : No dose change of 5-FU or TPFB delay.

Grade 3-4: Hold TPFB up to one week for resolution of AEs to grade 2 or less, then re-treat with one dose level reduction . If AEs not resolved to grade 2 after 1 week, discontinue TPFB treatment.

7. ADVERSE EVENTS: LIST AND REPORTING REQUIREMENTS

Adverse event (AE) monitoring and reporting is a routine part of every clinical trial. The following list of AEs (Section 7.1) and the characteristics of an observed AE (Section 7.2) will determine whether the event requires expedited (via AdEERS) reporting **in addition** to routine reporting.

7.1 Comprehensive Adverse Events and Potential Risks List (CAEPR)

7.1.1 CAEPRs for CTEP-Supplied Investigational Agent Bevacizumab (NSC #704865)

The Comprehensive Adverse Event and Potential Risks list (CAEPR) provides a single, complete list of reported and/or potential adverse events (AE) associated with an agent using a uniform presentation of events by body system. In addition to the comprehensive list, a subset, the Agent Specific Adverse Event List (ASAEL), appears in a separate column and is identified with ***bold*** and ***italicized*** text. This subset of AEs (the ASAEL) contains events that are considered 'expected' for expedited reporting purposes only. Refer to the "CTEP, NCI Guidelines: Adverse Event Reporting Requirements" (http://ctep.cancer.gov/reporting/adeers.html) for further clarification. The CAEPR may not provide frequency data; if not, refer to the Investigator's Brochure for this information.

Version 1.2, June 19, 2007[1]

**Category (Body System)**	**Adverse Events with Possible Relationship to Bevacizumab (CTCAE v3.0 Term)**	**'Agent Specific Adverse Event List' (ASAEL)**
ALLERGY/IMMUNOLOGY		
	Allergic reaction/hypersensitivity (including drug fever)	***Allergic reaction/hypersensitivity (including drug fever)***
	Allergic rhinitis (including sneezing, nasal stuffiness, postnasal drip)	***Allergic rhinitis (including sneezing, nasal stuffiness, postnasal drip)***
BLOOD/BONE MARROW		
	Hemoglobin	***Hemoglobin***
	Leukocytes (total WBC)	***Leukocytes (total WBC)***
	Neutrophils/granulocytes (ANC/AGC)	***Neutrophils/granulocytes (ANC/AGC)***
CARDIAC ARRHYTHMIA		
	Supraventricular arrhythmia NOS	***Supraventricular arrhythmia NOS***
	Ventricular fibrillation	
CARDIAC GENERAL		
	Cardiac ischemia/infarction	***Cardiac ischemia/infarction***
	Cardiac troponin I (cTnI)	
	Hypertension	***Hypertension***
	Hypotension	
	Left ventricular diastolic dysfunction	
	Left ventricular systolic dysfunction	
CONSTITUTIONAL SYMPTOMS		
	Fatigue (asthenia, lethargy, malaise)	***Fatigue (asthenia, lethargy, malaise)***
	Fever (in the absence of neutropenia, where neutropenia is defined as ANC <1.0 x 10^9/L)	***Fever (in the absence of neutropenia, where neutropenia is defined as ANC <1.0 x 10^9/L)***
	Rigors/chills	***Rigors/chills***
	Weight loss	
DERMATOLOGY/SKIN		
	Pruritus/itching	***Pruritus/itching***
	Rash/desquamation	***Rash/desquamation***
	Ulceration	
	Urticaria (hives, welts, wheals)	***Urticaria (hives, welts, wheals)***
	Wound complication, non-infectious	
GASTROINTESTINAL		
	Anorexia	***Anorexia***
	Colitis	
	Constipation	***Constipation***
	Diarrhea	***Diarrhea***
	Fistula, GI - Select	
	Heartburn/dyspepsia	***Heartburn/dyspepsia***

Category (Body System)	Adverse Events with Possible Relationship to Bevacizumab (CTCAE v3.0 Term)	'Agent Specific Adverse Event List' (ASAEL)
	Ileus (functional obstruction of bowel, i.e., neuroconstipation)	
	Leak (including anastomotic), GI: large bowel	
	Mucositis/stomatitis (functional/symptomatic) - Select	***Mucositis/stomatitis (functional/symptomatic) - Select***
	Nausea	***Nausea***
	Perforation, GI - Select	
	Ulcer, GI - Select	
	Vomiting	***Vomiting***
HEMORRHAGE/BLEEDING		
	Hemorrhage, GI - Select	***Hemorrhage GI - Select***
	Hemorrhage, CNS	***Hemorrhage, CNS***
	Hemorrhage, GU: vagina	***Hemorrhage, GU: vagina***
	Hemorrhage, pulmonary/upper respiratory: lung	***Hemorrhage, pulmonary/upper respiratory: lung***
	Hemorrhage, pulmonary/upper respiratory: nose	***Hemorrhage, pulmonary/upper respiratory: nose***
INFECTION		
	Infection with normal ANC or Grade 1 or 2 neutrophils - Select	
	Infection with normal ANC or Grade 1 or 2 neutrophils - Select (pelvis, peritoneal cavity, rectum, scrotum, skin, wound)	
METABOLIC/LABORATORY		
	Alkaline phosphatase	***Alkaline phosphatase***
	ALT, SGPT (serum glutamic pyruvic transaminase)	***ALT, SGPT (serum glutamic pyruvic transaminase)***
	AST, SGOT (serum glutamic oxaloacetic transaminase)	***AST, SGOT (serum glutamic oxaloacetic transaminase)***
	Bilirubin (hyperbilirubinemia)	***Bilirubin (hyperbilirubinemia)***
	Creatinine	
	Proteinuria	***Proteinuria***
NEUROLOGY		
	CNS cerebrovascular ischemia	***CNS cerebrovascular ischemia***
	Dizziness	***Dizziness***
	Neurology - Other: (Leukoencephalopathy syndrome including reversible posterior leukoencephalopathy syndrome [RPLS])	
PAIN		
	Pain - abdomen NOS	***Pain - abdomen NOS***

**Category (Body System)**	**Adverse Events with Possible Relationship to Bevacizumab (CTCAE v3.0 Term)**	**'Agent Specific Adverse Event List' (ASAEL)**
	Pain - chest/thorax NOS	***Pain - chest/thorax NOS***
	Pain - head/headache	***Pain - head/headache***
	Pain - joint	***Pain - joint***
	Pain - muscle	
	Pain - NOS	
PULMONARY/UPPER RESPIRATORY		
	Bronchospasm, wheezing	
	Cough	***Cough***
	Dyspnea (shortness of breath)	***Dyspnea (shortness of breath)***
	Fistula, pulmonary/upper respiratory - Select	
	Nasal cavity/paranasal sinus reactions	***Nasal cavity/paranasal sinus reactions***
	Voice changes/dysarthria (e.g., hoarseness, loss or alteration in voice, laryngitis)	***Voice changes/dysarthria (e.g., hoarseness, loss or alteration in voice, laryngitis)***
	Pulmonary/Upper Respiratory - Other (nasal-septal perforation)	
RENAL/GENITOURINARY		
	Fistula, GU - Select	
	Renal failure	
SYNDROMES		
	Cytokine release syndrome/acute infusion reaction	***Cytokine release syndrome/acute infusion reaction***
VASCULAR		
	Thrombosis/thrombus/embolism	***Thrombosis/thrombus/embolism***
	Visceral arterial ischemia (non-myocardial)	

[1]This table will be updated as the toxicity profile of the agent is revised. Updates will be distributed to all Principal Investigators at the time of revision. The current version can be obtained by contacting ADEERSMD@tech-res.com. Your name, the name of the investigator, the protocol and the agent should be included in the e-mail.

Also reported on bevacizumab trials but with the relationship to bevacizumab still undetermined:

BLOOD/BONE MARROW - Idiopathic thrombocytopenia purpura; platelets

CARDIAC GENERAL - Cardiac arrest; pericardial effusion; pulmonary hypertension

COAGULATION - DIC

DEATH - Sudden death (cause unknown)

DERMATOLOGY/SKIN - Hypopigmentation

GASTROINTESTINAL - Rectal abscess/necrosis; small bowel obstruction; taste

alteration

METABOLIC/LABORATORY - Hyperglycemia; hypoglycemia; hypomagnesemia; hyponatremia

MUSCULOSKELETAL/SOFT TISSUE - Aseptic necrotic bone; gait/walking; myasthenia gravis

NEUROLOGY - Aseptic meningitis; confusion; peripheral neuropathy; seizure; syncope

OCULAR/VISUAL - Cataract; watery eye

PULMONARY/UPPER RESPIRATORY - ARDS; pneumonitis/pulmonary infiltrates; pneumothorax

RENAL/GENITOURINARY - Urinary frequency

Note: Bevacizumab in combination with other agents could cause an exacerbation of any adverse event currently known to be caused by the other agent, or the combination may result in events never previously associated with either agent.

7.1.2 **Adverse Effects of *[^{18}F]FMISO injection***

No adverse events have been reported for diagnostic [^{18}F]FMISO administration at the strength described for this study in over 300 patients studied. Thus no adverse effects are expected as a result of the administration of [^{18}F]FMISO. The proposed [^{18}F]FMISO dose is less than 0.001 of the recommended safe oral dose assuming 2.5 m^2 std woman and 83 mg/m^2 (12 g/m^2 divided by 18 days) in one day. Significant adverse effects related to the study agent will be reported for up to a period of 24 hours post injection. Unexpected but potential adverse effects are listed below.

- Infection at the injection site or systemic infection
- Extravasation of the dose
- Allergic reaction
- Nausea, vomiting and anorexia
- Peripheral neuropathy
- Secondary cancer from radiation (chemo or radiation therapy would be the most likely cause)
- Death

7.2 Adverse Event List(s) for Commercial Agent(s)

7.2.1 Docetaxel is commercially available. See package insert for details.

Side Effects may include:

1. Cardiac: arrhythmias, pericardial effusions.
2. Hematologic: dose-related neutropenia, leukopenia, thrombocytopenia, anemia, hypoglycemia, hypernatremia.
3. Gastrointestinal: nausea and vomiting, diarrhea, oral mucositis, pancreatitis, esophagitis.
4. Neurologic: reversible dysthesias or paresthesias, peripheral neuropathy, mild or moderate lethargy or somnolence, headache, seizures.
5. Hypersensitivity: hypersensitivity (local or general skin rash, flushing, pruritus, drug-fever, chills and rigors, low back pain), severe anaphylactoid reactions (flushing with hypo- or hypertension, with or without dyspnea).
6. Dermatologic: alopecia, desquamation following localized pruriginous maculopapular eruption, skin erythema with edema, extravasation reaction (erythema, swelling, tenderness, pustules), reversible peripheral phlebitis, nail changes.
7. Hepatic: increased transaminase, alkaline phosphatase, bilirubin; hepatic failure; hepatic drug reaction.
8. Pulmonary: dyspnea with restrictive pulmonary syndrome, pleural effusions.
9. Other: asthenia, dysgeusia, anorexia, conjunctivitis, arthralgia, muscle aches, myopathy, peripheral edema, fluid retention syndrome, ascites.
   Prolonged treatment with weekly docetaxel results in chronic toxicities, which include asthenia (fatigue), anemia, edema, excessive lacrimation (epiphora), and onycholysis.

<u>7.2.2 Cisplatin</u> is commercially available. See package insert for details.
Side Effects may include:

1. Hematologic: Leukopenia and thrombocytopenia occur, but are rarely dose-limiting; anemia.
2. Dermatologic: Alopecia (uncommon).
3. Gastrointestinal: Nausea and vomiting are common and may persist for up to 24-96 hours; anorexia.
4. Renal: Nephrotoxicity is dose-related and relatively uncommon with adequate hydration and diuresis; elevated serum creatinine and BUN.

5. Hepatic: Elevated AST and ALT.
6. Neurologic: Peripheral neuropathy (paresthesias), common and dose-limiting when the cumulative cisplatin dose exceeds 400 mg/m²; rarely seizures; ototoxicity manifested initially by high frequency hearing loss; vestibular toxicity (dizziness) uncommon; tetany (caused by hypomagnesemia); rarely Lhermitte's sign.
8. Other: Hypomagnesemia, hypocalcemia, hyponatremia, vein irritation, papilledema, rarely retrobulbar neuritis, rarely anaphylaxis, fatigue.

7.2.3 Carboplatin is commercially available. See package insert for details. Side Effects may include:

1. Hematologic: Thrombocytopenia, neutropenia, leukopenia, more pronounced in patients with compromised renal function and heavily pretreated patients; may be cumulative.
2. Gastrointestinal: Nausea and vomiting (less severe than with cisplatin), treatable with moderate doses of antiemetics.
3. Dermatologic: Rash, urticaria.
4. Hepatic: Abnormal liver function tests, usually reversible with standard doses.
5. Neurologic: Rarely peripheral neuropathy.
6. Renal: Elevations in serum creatinine, BUN, electrolyte loss (Na, Mg, K, Ca).

7.Other: Pain, asthenia.

7.2.4 5- Fluorouracil is commercially available. See package insert for details. Side Effects may include:

Hematologic: Leukopenia, thrombocytopenia, anemia (can be dose limiting, less common with continuous infusion); Dermatologic: Dermatitis, nail changes, hyperpigmentation, Hand-Foot Syndrome with protracted infusions, alopecia; Gastrointestinal: Nausea, vomiting, anorexia, diarrhea (can be dose limiting); mucositis

(is common with 5-day infusion, occasionally dose limiting); Neurologic: Cerebellar Syndrome (headache and cerebellar ataxia); Cardiac: Angina, noted with continuous infusion; Ophthalmic: Eye irritation, nasal discharge, watering of eyes, blurred vision.

7.3 **Adverse Event Characteristics**

- **CTCAE term (AE description) and grade:** The descriptions and grading scales found in the revised NCI Common Terminology Criteria for Adverse Events (CTCAE) version 3.0 will be utilized for AE reporting. All appropriate treatment areas should have access to a copy of the CTCAE version 3.0. A copy of the CTCAE version 3.0 can be downloaded from the CTEP web site (http://ctep.cancer.gov).

- **'Expectedness'**: AEs can be 'Unexpected' or 'Expected' (see Section 7.1 above) for expedited reporting purposes only. 'Expected' AEs (the ASAEL) are ***bold and italicized*** in the CAEPR (Section 7.1.1).

- **Attribution** of the AE:

- Definite – The AE *is clearly related* to the study treatment.
- Probable – The AE *is likely related* to the study treatment.
- Possible – The AE *may be related* to the study treatment.
- Unlikely – The AE *is doubtfully related* to the study treatment.
- Unrelated – The AE *is clearly NOT related* to the study treatment.

7.4 **Expedited Adverse Event Reporting**

7.4.1 Expedited AE reporting for this study must use AdEERS (Adverse Event Expedited Reporting System), accessed via the CTEP home page (http://ctep.cancer.gov). The reporting procedures to be followed are presented in the "CTEP, NCI Guidelines: Adverse Event Reporting Requirements" which can be downloaded from the CTEP home page (http://ctep.cancer.gov). These requirements are briefly outlined in the table below (Section 7.4.3).

In the rare occurrence when Internet connectivity is lost, an AE report may be submitted using CTEP's Adverse Event Expedited Report-Single Agent or Multiple Agent paper template (available at http://ctep.cancer.gov) and faxed to 301-230-0159. A 24-hour notification is to be made to CTEP by telephone at 301-897-7497, only when Internet connectivity is disrupted. Once Internet connectivity is restored, an AE report submitted on a paper template or a 24-hour notification phoned in must be entered electronically into AdEERS by the original submitter at the site.

7.4.2 AdEERS is programmed for automatic electronic distribution of reports to the following individuals: Study Coordinator of the Lead Organization, Principal Investigator, and the local treating physician. AdEERS provides a copy feature for other

e-mail recipients.

7.4.3 **<u>Expedited Reporting Guidelines</u>**

<u>Note that these guidelines apply to both bevacizumab and F- MISO **EXCEPT** </u>***that F-MISO related events are defined as AEs occurring within 24 hours of the infusion.***

– AdEERS Reporting Requirements for Adverse Events that occur within 30 Days[1] of the Last Dose of the Investigational Agent on Phase 2 and 3 Trials

**Phase 2 and 3 Trials**									
	**Grade 1**	**Grade 2**	**Grade 2**	**Grade 3**		**Grade 3**		**Grades 4 & 5[2]**	**Grades 4 & 5[2]**
	**Unexpected and Expected**	**Unex-pected**	**Expected**	**Unexpected** with Hospitali-zation	**Unexpected** without Hospitali-zation	**Expected** with Hospitali-zation	**Expected** without Hospitali-zation	**Unex-pected**	**Expected**
**Unrelated Unlikely**	Not Required	Not Required	Not Required	10 Calendar Days	Not Required	10 Calendar Days	Not Required	10 Calendar Days	10 Calendar Days
**Possible Probable Definite**	Not Required	10 Calendar Days	Not Required	10 Calendar Days	10 Calendar Days	10 Calendar Days	Not Required	24-Hour; 5 Calendar Days	10 Calendar Days

[1] **Adverse events with attribution of possible, probable, or definite that occur <u>greater</u> than 30 days after the last dose of treatment with an agent under a CTEP IND require reporting as follows:**

AdEERS 24-hour notification followed by complete report within 5 calendar days for:

- Grade 4 and Grade 5 unexpected events

  AdEERS 10 calendar day report:
- Grade 3 unexpected events with hospitalization or prolongation of hospitalization
- Grade 5 expected events

[2] Although an AdEERS 24-hour notification is not required for death clearly related to progressive disease, a full report is required as outlined in the table.

December 15, 2004

Note: All deaths on study require both routine and expedited reporting regardless of causality. Attribution to treatment or other cause must be provided.

- Expedited AE reporting timelines defined:

- "24 hours; 5 calendar days" – The investigator must initially report the AE via AdEERS within 24 hours of learning of the event followed by a complete AdEERS report within 5 calendar days of the initial 24-hour report.
- "10 calendar days" - A complete AdEERS report on the AE must be submitted within 10 calendar days of the investigator learning of the event.

- Any medical event equivalent to CTCAE grade 3, 4, or 5 that precipitates hospitalization (or prolongation of existing hospitalization) must be reported regardless of attribution and designation as expected or unexpected with the exception of any events identified as protocol-specific expedited adverse event reporting exclusions.

- Any event that results in persistent or significant disabilities/incapacities, congenital anomalies, or birth defects must be reported via AdEERS if the event occurs following treatment with an agent under a CTEP IND.

- Use the NCI protocol number and the protocol-specific patient ID assigned during trial registration on all reports.

7.4.4 Protocol-Specific Expedited Adverse Event Reporting Requirements

For this protocol only, certain additional AEs/grades are required to be reported via the AdEERS SAE reporting mechanism in addition to those in the generic Expedited Reporting Guidelines section. The following AEs must be reported through the routine reporting mechanism as well as the AdEERS system.

Bevacizumab-specific AdEERS reporting requirements:

Note: The below SAEs must be reported to AdEERS regardless of the interval from the last dose of bevacizumab.

CTCAE Category	Adverse Event	Grade (or higher)	Attribution	Comments
Cardiac	Hypertension	4	Bevacizumab	
cardiac	Cardiac ischemia	3	Bevacizumab	
CNS cerebrovascular ischemia	CNS ischemia	3	Bevacizumab	
Vascular	Peripheral/visceral arterial ischemia	3	Bevacizumab	
Hemorrhage / Bleeding	Henorrhage	3	Bevacizumab	Any site

GI, Pulmonary, other	Perforation, fistula and leak	2	Bevacizumab	Any site
Metabolic/ laboratory	proteinuria	3	Bevacizumab	
Neurology	Other- Reversible Posterior Leukoencephalopathy Syndrome (RPLS)	1	Bevacizumab	
Dermatology/ skin	Wound complication	3	all	Any site

7.5 **Routine Adverse Event Reporting**

All Adverse Events **must** be reported in routine study data submissions. **AEs reported through AdEERS must also be reported in routine study data submissions.**

7.6 **Secondary AML/MDS**

Investigators are required to report cases of secondary AML/MDS occurring on or following treatment on NCI-sponsored chemotherapy protocols using the NCI/CTEP Secondary AML/MDS Report Form. This form can be downloaded from the CTEP web site (http://ctep.cancer.gov). Refer to the "CTEP, NCI Guidelines: Adverse Event Reporting Requirements" (available at http://ctep.cancer.gov) for additional information about secondary AML/MDS reporting.

8. PHARMACEUTICAL INFORMATION

A list of the adverse events and potential risks associated with the investigational or commercial agents administered in this study can be found in Section 7.1.

8.1 **CTEP-Supplied Investigational Agents**

8.1.1 **Bevacizumab** (rhuMAb VEGF, Avastin™) NSC # 704865

Bevacizumab is a humanized IgG1 monoclonal antibody (MAb) that binds all biologically active isoforms of human vascular endothelial growth factor **(**VEGF, or VEGF-A) with high affinity (k_d = 1.1 nM) [66]. The antibody consists of a human IgG1 framework and the antigen-binding complementarity-determining regions from the murine anti-VEGF MAb A.4.6.1 See Avastin^{TM} [bevacizumab] Investigators Brochure. [66, 67] for details.

Mechanism of Action

Of known pro-angiogenic factors, VEGF is one of the most potent and specific, and has been identified as a crucial regulator of both normal and pathological angiogenesis. VEGF is a secreted, heparin-binding protein that exists in multiple isoforms. Action of VEGF is primarily mediated through binding to the receptor tyrosine kinases, VEGFR-1 (Flt-1) and VEGFR-2 (KDR/Flk-1). The biological effects of VEGF include endothelial cell mitogenesis and migration, increased vascular permeability, induction of proteinases leading to remodeling of the extracellular matrix, and suppression of dendritic cell maturation. Neutralization of VEGF by A.4.6.1 or bevacizumab has been shown to inhibit the VEGF-induced proliferation of human endothelial cells *in vitro*, and decrease microvessel density and interstitial pressure in tumor xenografts *in vivo*. In patients, preliminary results from a neoadjuvant trial in rectal cancer demonstrated a decrease in blood perfusion/permeability and interstitial fluid pressure in tumors after one dose of bevacizumab. [42]

The specific potential clinical relevance of bevacizumab in patients with NPC and in combination with other treatments in this trial is discussed in the general background section of this protocol.

Nonclinical Studies

The murine parent MAb of bevacizumab, A4.6.1, has demonstrated potent growth inhibition *in vivo* in a variety of human cancer xenograft and metastasis models, including those for SK-LMS-1 leiomyosarcoma, G55 glioblastoma multiforme, A673 rhabdo myosarcoma, Calu-6, and MCF-7 cell lines. [66-68]The antitumor activity was enhanced with the combination of A4.6.1 and chemotherapeutic agents compared to either agent alone. Combined blockage of the VEGF and other growth factor pathways (*e.g.*, EGFR or PDGFR) has also demonstrated additive effects *in vivo.* [69] [70] Associated with the anti-tumor activity of anti-VEGF MAbs were findings of reduced intra-tumoral endothelial cells and microcapillary counts as well as reduced vascular permeability and interstitial pressure

Nonclinical toxicology studies have examined the effects of bevacizumab on female reproductive function, fetal development, and wound healing. Fertility may be impaired in cynomolgus monkeys administered bevacizumab, which led to reduced endometrial proliferation and uterine weight as well as a decrease in ovarian weight and number of corpora lutea. Bevacizumab is in rabbits, with increased frequency of fetal resorption, specific gross and skeletal alterations. In juvenile cynomolgus monkeys with open growth plates, bevacizumab induced epophyseal dysplasia which was partially reversible upon cessation of therapy. Bevacizumab also delays the rate of wound healing in rabbits, and this effect appeared to be dose-dependent and characterized by a reduction of wound tensile strength.

Clinical Studies

To date, over 7000 patients have been treated in clinical trials with bevacizumab as monotherapy or in combination regimens (Bevacizumab Investigator's Brochure).

The pharmacokinetics (PK) of bevacizumab have been characterized in several phase 1 and phase 2 clinical trials, with doses ranging from 1 to 20 mg/kg administered weekly,

every 2 weeks, or every 3 weeks. The estimated half-life of bevacizumab is approximately 21 days (range 11-50 days). The predicted time to reach steady state was 100 days. The volume of distribution is consistent with limited extravascular distribution.

The maximum tolerated dose (MTD) of bevacizumab has not been determined; however, the dose level of 20 mg/kg was associated severe headaches . [71] The dose schedule of either 10 mg/kg q2w, or 15 mg/kg q3w is used in most phase 2 or 3 trials with only a few exceptions (*e.g*., the pivotal phase 3 trial in colorectal cancer, in which bevacizumab was given at 5 mg/kg q2w).

Clinical proof of principle for anti-VEGF therapy with bevacizumab has been observed in several solid tumors. In 1st- and 2nd-line metastatic colorectal cancer, combination of bevacizumab with 5-FU-based chemotherapy improved the overall survival (OS), progression-free survival (PFS) and response rate (RR) as compared to chemotherapy alone .[72] There was also improved overall survival in first-line NSCLC patients (E4599) treated with carboplatin/paclitaxel + bevacizumab compared with chemotherapy alone. Bevacizumab in combination with chemotherapy has been approved by the FDA for treatment in advanced/metastatic colorectal cancer (first and second lines) and in NSCLC.

In untreated advanced and metastatic breast cancer, addition of bevacizumab to paclitaxel also significantly improved the RR and PFS. [73]However, in the phase 3 trial in doxorubicin and paclitaxel-refractory metastatic breast cancer, the addition of bevacizumab to capecitabine did not show an improvement in PFS despite an increase in the RR. [73] . In locally advanced and metastatic pancreatic cancer, a Phase III also failed to demonstrate OS or PFS advantage by adding bevacizumab to gemcitabine (CALGB 80303) .

Bevacizumab has been studied as monotherapy in renal cell cancer (RCC). In a 3-arm, double-blind, placebo-controlled phase 2 trial [74],patients with previously treated stage IV RCC were randomized to high-dose (HD) bevacizumab (10 mg/kg q2w), low-dose (LD) bevacizumab (3 mg/kg q2w) or placebo. The study demonstrated a highly significant prolongation of time to progression (TTP) in the HD arm (4.8 months) as compared with the placebo (2.6 months) (hazard ratio = 2.55, p = 0.0002); the LD arm was associated with a smaller difference in TTP (3.0 months) of borderline significance. The tumor response rate was 10% in the HD arm but 0% in the LD and placebo groups.

Additional clinical trials are ongoing in a variety of solid tumors and hematological malignancies using bevacizumab as monotherapy or in combination with chemotherapy, radiation, or other targeted/biological agents.

Formulation

Bevacizumab is a recombinant humanized anti-VEGF monoclonal antibody. consisting of 93% human and 7% murine amino acid sequences. The antibody consists of a human IgG1 framework and the antigen-binding complementarity-determining regions from the murine anti- VEGF MAb A.4.6.1;17,20,23 approximate molecular weight is 149,000 daltons.

Availability

Bevacizumab is supplied as a clear to slightly opalescent, sterile liquid ready for parenteral administration. - Each 400 mg (25mg/ml – 16 mL fill) glass vial contains bevacizumab with phosphate, trehalose, polysorbate 20, and Sterile Water for Injection, USP.

Storage and Stability

Upon receipt, bevacizumab should be refrigerated (2 to 8 degrees Centigrade). Do not freeze. Do not shake. Shelf-life studies of rhuMAb VEGF are ongoing. The sterile single use vials contain no antibacterial preservatives. Therefore, vials should be discarded 8 hours after initial entry. Once diluted in 0.9% sodium chloride, solutions of bevacizumab must be administered within 8 hours.

Route of Administration

Intravenous

Preparation

Vials contain no preservatives and are intended for single use only. Place the calculated dose in 100 mL of 0.9% sodium chloride for injection.

Safety Profile

Based on clinical trials with bevacizumab as monotherapy or in combination with chemotherapy, the most common adverse events of any severity include asthenia, pain, headache, hypertension, diarrhea, stomatitis, constipation, epistaxis, dyspnea, dermatitis and proteinuria. The most common grade 3-4 adverse events were asthenia, pain, hypertension, diarrhea and leukopenia.

The most serious AEs include life-threatening or fatal hemorrhage, arterial thromboembolic events, gastrointestinal perforation and wound dehiscence; these events were uncommon but occurred at an increased frequency compared to placebo or chemotherapy controls in randomized studies. Increased rates of severe neutropenia have been observed in patients treated with some chemotherapy regimens plus bevacizumab. Other SAEs observed with bevacizumab therapy include hypertensive crisis, nephrotic syndrome and reversible posterior leukoencephalopathy syndrome.

The following is a description of major adverse events associated with bevacizumab therapy. A list of Comprehensive Adverse Events and Potential Risks (CAEPR) in NCI-CTCAE v3.0 terms is included below. Please see the Investigators' Brochure and the FDA package insert (www.fda.gov/cder/foi/label/2004/125085lbl.pdf) for additional details.

Infusion-Related Reactions: Infusion reactions with bevacizumab were uncommon (<3%) and rarely severe (0.2%). Infusion reactions may include rash, urticaria, fever, rigors, hypertension, hypotension, wheezing, or hypoxia. Currently, there is no adequate information on the safety of retreatment with bevacizumab in patients who have experienced severe infusion-related reactions.

Hypertension: Hypertension is common in patients treated with bevacizumab, with an incidence of 20-30% (all grade) across trials, with a mean increase of +5.5mmHg to

+8.4mmHg for systolic pressure, or +4.1mmHg to +5.4mmHg for diastolic pressure. Incidence of grade 3 (hypertension requiring initiation of or increase in hypertensive medications) ranges from 7.8 to 17.9%. Grade 4 hypertension (hypertensive crisis) occurred in up to 0.5% of bevacizumab-treated patients.

Hypertension associated with bevacizumab can generally be controlled with routine oral drugs while bevacizumab is continued. However, incidents of hypertensive crisis with encephalopathy (including RPLS – reversible posterior leukoencephalopathy syndrome – see below) or cardiovascular sequelae have been rarely reported. BP should be closely monitored during bevacizumab therapy and the goal of BP control should be consistent with standard medical practice. [75] Bevacizumab therapy should be suspended in the event of uncontrolled hypertension.

<u>Proteinuria</u>: Proteinuria has been seen in all bevacizumab studies to date, ranging in severity from mild asymptomatic increase in urine protein (incidence of about 38%) to rare instances of grade 3 proteinuria (> 3.5gm/24 hour urine) (3%) or nephrotic syndrome (1.4%). Pathologic findings on renal biopsies in two patients showed proliferative glomerulonephritis. The risk of proteinuria may be higher in patients with advanced RCC or history of hypertension. There is also evidence that the rate of proteinuria may be dose related.

<u>Hemorrhage</u>: An increased incidence of bleeding events was observed in patients treated with bevacizumab as compared to control treatment arms. In the bevacizumab-containing treatment arms of clinical trials (across all indications), the incidence rate of NCI-CTC Grade ≥ 3 bleeding events ranged from 0.4-5%, compared to 0-2.9% in control treatment arms. <u>**The hemorrhagic events that have been observed in bevacizumab clinical studies were predominantly tumor-associated hemorrhage and minor mucocutaneous hemorrhage.**</u>

Tumor-associated hemorrhage - Major or massive pulmonary hemorrhage/hemoptysis has been observed primarily in patients with NSCLC. In a phase 2 study in NSCLC, 6 cases of life-threatening (4 fatal) hemoptysis were reported among 66 patients treated with bevacizumab and chemotherapy [76]; squamous cell histology was identified as the risk factor. In the phase III trial in non-squamous NSCLC (E4599), the rate of Grade ≥ 3 pulmonary hemorrhage was <1% in the control arm (carboplatin/paclitaxel) versus 2.3% in the chemotherapy plus bevacizumab arm (10/427 patients, including 7 deaths). ***Many patients who experienced pulmonary hemorrhages requiring medical intervention had cavitation and/or necrosis of the tumor, either pre-existing or developing during bevacizumab therapy. Patients developing tumor cavitation on treatment should be assessed by the treating physician for risk-benefit.***

Gastrointestinal hemorrhages, including rectal bleeding and melena have been reported in patients with colorectal cancer, and have been assessed as tumor-associated hemorrhages. In the pivotal phase 3 trial in advanced colorectal cancer, the rate of GI hemorrhage (all grades) was 24% in the IFL/bevacizumab arm compared to 6% in the IFL arm; grade 3-4 hemorrhage was 3.1% for IFL/bevacizumab and 2.5% for IFL. Serious tumor associated bleedings have also been observed in patients with pancreatic cancer, gastric cancer, soft tissue sarcoma, CNS metastases, hepatoma or varices treated with bevacizumab.

Mucocutaneous hemorrhage - Across all bevacizumab clinical trials, mucocutaneous hemorrhage has been seen in 20%-40% of patients treated with bevacizumab. These were most commonly NCI-CTC Grade 1 epistaxis that lasted less than 5 minutes, resolved without medical intervention and did not require any changes in bevacizumab treatment regimen.

There have also been less common events of minor mucocutaneous hemorrhage in other locations, such as gingival bleeding and vaginal bleeding.

Arterial Thromboembolic Events (ATE): The risk of arterial thromboembolic events is increased with bevacizumab therapy; such events included cerebral infarction, transient ischemic attack (TIA), myocardial infarction (MI) and other peripheral or visceral arterial thrombosis. A pooled analysis of five randomized studies showed a two-fold increase in these events (3.8% vs. 1.7%). ATE led to a fatal outcome in 0.8% patients with bevacizumab (vs. 0.5% without bevacizumab). The rate of cerebrovascular accidents (including TIA) was 2.3% vs. 0.5%, and the rates of MI 1.7% vs. 0.7%. Certain baseline characteristics, such as age and prior arterial ischemic events, appear to confer additional risk . [77] In patients ≥ 65 years treated with bevacizumab and chemotherapy, the rate of ATE was approximately 8.5%.

Aspirin is a standard therapy for primary and secondary prophylaxis of ATE in patients at high risk of such events, and the use of aspirin ≤ 325 mg daily was allowed in the five randomized studies discussed above, though safety analyses specifically regarding aspirin use were not preplanned. Due to the relatively small numbers of aspirin users and ATE events, retrospective analyses of the ability of aspirin to affect the risk of ATE were inconclusive. Further analyses of the effects of concomitant use of bevacizumab and aspirin are ongoing.

Venous thromboembolism (VTE) *(including deep venous thrombosis, pulmonary embolism and thrombophlebitis)* – In the Phase III pivotal trial in metastatic CRC, there was a slightly higher rate of VTE in patients treated with chemotherapy + bevacizumab compared with chemotherapy alone (19% vs. 16%). The incidence of NCI-CTC Grade≥ 3 VTEs in one NSCLC trial (E4599) was higher in the bevacizumab-containing arm compared to the chemotherapy control arm (5.6% vs. 3.2%).

In clinical trials across all indications the overall incidence of VTEs ranged from 2.8% to 17.3% in the bevacizumab-containing arms compared to 3.2% to 15.6% in the chemotherapy control arms. The use of bevacizumab with chemotherapy does not substantially increase the risk of VTE compared with chemotherapy alone. However, patients with mCRC who receive bevacizumab and experienced VTE may be at higher risk for recurrence of VTE.

Gastrointestinal Perforation: GI perforations/fistula were rare but occurred at an increased rate in bevacizumab-containing therapies. The majority of such events required surgical intervention and some were associated with a fatal outcome. In the pivotal phase 3 trial in CRC (AVF2107), the incidence of bowel perforation was 2% in patients receiving IFL/bevacizumab and 4% in patients receiving 5-FU/bevacizumab compared to 0.3% in patients receiving IFL alone. GI perforation has also been reported in non-CRC tumors

(e.g. gastric/esophageal, pancreatic and ovarian cancers) or nonmalignant conditions such as diverticulitis and gastric ulcer. GI perforation should be included in the differential diagnosis of patients on bevacizumab therapy presenting with abdominal pain or rectal/abdominal abscess.

Fistulae that involve areas other than the GI tract have also been observed (e.g. tracheoesophageal, bronchopleural, urogenital, biliary). For example, life-threatening or fatal tracheoesophageal fistula has been reported in patients with small cell lung cancer treated with concurrent chemoradiation and bevacizumab. In a phase II trial of irinotecan + carboplatin + RT and bevacizumab followed by maintenance bevacizumab that accrued 25 patients, there have been two confirmed cases of tracheoesophageal (TE) fistula (one fatal) and a third case of fatal upper aerodigestive tract hemorrhage, with TE fistula suspected but not confirmed. All three events occurred during the bevacizumab maintenance phase (1.5 to 4 months after completion of chemoradiation). While pulmonary fistula (including TE fistula) has also been observed in advanced NSCLC or SCLC patients receiving bevacizumab and chemotherapy (without radiation), the incidence was extremely low.

Wound Healing Complications: Bevacizumab delays wound healing in rabbits, and it may also compromise or delay wound healing in patients. Bowel anastomotic dehiscence and skin wound dehiscence have been reported in clinical trials with bevacizumab.

The appropriate interval between surgery and initiation of bevacizumab required to avoid the risk of impaired wound healing has not been determined. Across metastatic CRC trials, at least 28 days must have elapsed following major surgery before bevacizumab could be initiated; data suggested initiation of bevacizumab 29-60 days following surgery did not appear to increase the risk of wound healing complications compared to those treated with chemotherapy alone.

The optimal interval between termination of bevacizumab and subsequent elective surgery has not been determined. In the pivotal study in CRC, among patients who underwent major surgery while on study therapy, there was an increased rate of significant post-operative bleeding or wound healing complications in the IFL + bevacizumab arms vs. IFL alone [10% (4/40) vs. 0% (0/25)]. [78]Decisions on the timing of elective surgery should take into consideration the half-life of bevacizumab (average 21 days, range 11-50 days).

If patients receiving treatment with bevacizumab require elective major surgery, it is recommended that bevacizumab be held for 4–8 weeks prior to the surgical procedure. Patients undergoing a major surgical procedure should not begin/restart bevacizumab until 4 weeks after that procedure (in the case of high-risk procedures such as liver resection, thoracotomy, or neurosurgery, it is recommended that chemotherapy be restarted no earlier than 6 weeks and bevacizumab no earlier than 8 weeks after surgery).

Congestive Heart Failure: The risk of left ventricular dysfunction may be increased in patients with prior or concurrent anthracycline treatment. In phase 3 controlled clinical trials in metastatic breast cancer (AVF 2119g) in which all patients had received prior anthracyclines, congestive heart failure (CHF) or cardiomyopathy were reported in 7 patients (3%) in the bevacizumab+capecitabine arm compared to 2 (1%) in the capecitabine-only arm. A recently published phase II study in subjects with refractory

acute myelogenous leukemia reported 5 cases of cardiac dysfunction (CHF or decreases to <40% in left ventricular ejection fraction) of 48 subjects treated with sequential cytarabine, mitoxantrone, and bevacizumab. All but one of these subjects had significant prior exposure to anthracyclines as well. [79] Other studies are ongoing in this patient population. Patients receiving anthracyclines or with prior exposure to anthracyclines should have a baseline MUGA or ECHO with a normal ejection fraction.

Reversible Posterior Leukoencephalopathy Syndrome (RPLS), Posterior Reversible Encephalopathy Syndrome (PRES) or similar leukoencephalopathy syndrome: RPLS/PRES are clinical syndromes related to vasogenic edema of the white matter and there have rarely reported in association with bevacizumab therapy (<1%). Clinical presentations may include altered mental status, seizure and cortical blindness. MRI scans are required for diagnosis; typical findings are vasogenic edema predominantly in the white matter of the posterior parietal and occipital lobes, and less frequently in the anterior distributions and the gray matter. In RPLS associated with bevacizumab, mild or significant BP elevations were seen in some but not all cases. [80-82]

RPLS/PRES should be in the differential diagnosis in patients presenting with unexplained mental status change, visual disturbance, or seizure; hypertension may or may not be present. This syndrome is potentially reversible, but timely correction of the underlying causes, including control of BP and interruption of the offending drug, is important in order to prevent irreversible tissue damage.

Neutropenia: When combined with chemotherapy, bevacizumab may increase the risk of neutropenia compared to chemotherapy alone. In a phase 3 trial with IFL +/- bevacizumab in colorectal cancer, grade 3-4 neutropenia was 21% in the bevacizumab + IFL arm vs. 14% in the IFL arm (grade 4 neutropenia was 3% vs. 2%). In a phase 3 trial with carboplatin and paclitaxel +/- bevacizumab in NSCLC, the bevacizumab-containing arm was associated with an increased rate of grade 4 neutropenia (27% vs. 17%), febrile neutropenia (5.4% vs. 1.8%), and an increased rate of infection with neutropenia (4.4% vs. 2.0%) with three fatal cases in the bevacizumab + chemotherapy arm vs. none in the chemotherapy control arm.

Fertility and Pregnancy: Clinical data are lacking regarding the immediate or long-term effect of bevacizumab on fertility and pregnancy. However, bevacizumab is known to be teratogenic and detrimental to fetal development in animal models. In addition, bevacizumab may alter corpus luteum development and endometrial proliferation, thereby having a negative effect on fertility. As an IgG1, it may also be secreted in human milk. Therefore, fertile men and women on bevacizumab studies must use adequate contraceptive measures and women should avoid breast feeding. The duration of such precautions after discontinuation of bevacizumab should take into consideration the half-life of the agent (average 21 days, ranging from 11 to 50 days).

Immunogenicity**:** As a therapeutic protein, there is a potential for immunogenicity with bevacizumab. With the currently available assay with limited sensitivity, high titer human anti-bevacizumab antibodies have not been detected in approximately 500 patients treated with bevacizumab.

Treatment

Bevacizumab is administered by IV infusion. The dose should be based on the patient's actual body weight; the dose will be recalculated if there is a weight change of > 10% from baseline.

The first dose of bevacizumab should be given over 90 minutes. If well tolerated, the second dose can be given over 60 minutes. If this dose is well-tolerated, then all subsequent infusions can be administered over 30 minutes. If an infusion reaction occurs, subsequent doses of bevacizumab should be administered over the shortest period that was well tolerated.

Special Precautions/Safety Issues:

- Prior to each treatment, the patient should be carefully assessed with special attention to blood pressure, proteinuria, bleeding and cardiovascular events, as well as symptoms or signs of bowel perforation and RPLS. Decisions for retreatment or dose modification/interruption should follow the dose modification guidelines.
- Infusional reactions: Routine premedication is not required for the first dose of bevacizumab. If infusional reactions occur, acetaminophen, diphenhydramine, steroids or other medications may be given for symptom control and for premedication as needed. Anaphylaxis precautions should be observed during bevacizumab administration.
- Hypertension: Hypertension is a known and potentially serious adverse event associated with bevacizumab treatment. Patients should have their BP monitored prior to each infusion of bevacizumab. Hypertensive mediation should be initiated or increased for optimal BP control according to standard public heath guidelines.
- Proteinuria: Proteinuria should be monitored by urine protein:creatinine (UPC) ratio at least every 6 weeks.

8.1.3 Agent Ordering

NCI-supplied agents may be requested by the Principal Investigator (or their authorized designees) at each participating institution. Pharmaceutical Management Branch (PMB) policy requires that the agent be shipped directly to the institution where the patient is to be treated. PMB does not permit the transfer of agents between institutions (unless prior approval from PMB is obtained). The CTEP-assigned protocol number must be used for ordering all CTEP-supplied investigational agents. The responsible investigator at each participating institution must be registered with CTEP, DCTD through an annual submission of FDA form 1572 (Statement of Investigator), Curriculum Vitae, Supplemental Investigator Data Form (IDF), and Financial Disclosure Form (FDF). If there are several participating investigators at one institution, CTEP-supplied investigational agents for the study should be ordered under the name of one lead investigator at that institution.

Agent may be requested by completing a Clinical Drug Request (NIH-986) and mailing it to the Pharmaceutical Management Branch, DCTD, NCI, 9000 Rockville Pike, EPN Room 7149, Bethesda, MD 20892-7422 or faxing it to (301) 480-4612. For questions call (301) 496-5725.

8.2 ***Commercial Agent(s)***

8.2.1 Docetaxel - (Commercially available. Please refer to the package insert for further information)

Other Names Taxotere, RP 56976, NSC #628503. Classification: Antimicrotubule agent.

Mode of Action: Docetaxel, a semisynthetic analog of paclitaxel, promotes the assembly of tubulin and inhibits microtubule depolymerization. Bundles of microtubules accumulate and interfere with cell division.

Storage and Stability:

Docetaxel infusion solution, if stored between 2 and 25ºC (36 and 77ºF) is stable

for 4 hours. Fully prepared docetaxel infusion solution (in either 0.9% Sodium

Chloride solution or 5% Dextrose solution) should be used within 4 hours

(including the administration time). Store between 2 and 25°C (36 and 77ºF). Retain in the original package toprotect from bright light. Freezing does not adversely affect the product.

Preparation:

Docetaxel is a cytotoxic anticancer drug and, as with other potentially toxic compounds, caution should be exercised when handling and preparing docetaxel solutions. The use of gloves is recommended. If docetaxel concentrate, initial diluted solution, or final dilution for infusion should come into contact with the skin, immediately and thoroughly wash with soap and water. If docetaxel concentrate, initial diluted solution, or final dilution for infusion should come into contact with mucosa, immediately and thoroughly wash with water. Docetaxel for Injection Concentrate requires two dilutions prior to administration.

Please follow the preparation instructions provided below. **Note:** Both the docetaxel for Injection Concentrate and the diluent vials contain an overfill.

A. Preparation of the Initial Diluted Solution

1. Gather the appropriate number of vials of docetaxel for Injection

Concentrate and diluent (13% Ethanol in Water for Injection). If the vials were refrigerated, allow them to stand at room temperature for approximately 5 minutes.

2. Aseptically withdraw the contents of the appropriate diluent vial into a

syringe and transfer it to the appropriate vial of docetaxel for Injection Concentrate. **If the procedure is followed as described, an initial diluted solution of 10mg docetaxel/mL will result.**

3. Mix the initial diluted solution by repeated inversions for at least 45

seconds to assure full mixture of the concentrate and diluent. Do not shake.

4. The initial diluted docetaxel solution (10 mg docetaxel/mL) should be

clear; however, there may be some foam on top of the solution due to the polysorbate 80. Allow the solution to stand for a few minutes to allow any foam to dissipate. It is not required that all foam dissipate prior to continuing the preparation process. The initial diluted solution may be used immediately or stored either in the refrigerator or at room temperature for a maximum of 8 hours.

B. Preparation of the Final Dilution for Infusion

1. Aseptically withdraw the required amount of initial diluted docetaxel

solution (10mg docetaxel/mL) with a calibrated syringe and inject into an infusion bag or bottle of either 0.9% Sodium Chloride solution or 5% Dextrose solution to produce a final concentration of 0.3 to 0.74mg/mL. Thoroughly mix the infusion by manual rotation.

2. As with all parenteral products, docetaxel should be inspected visually

for particulate matter or discoloration prior to administration whenever the solution and container permit. If the docetaxel for Injection, initial diluted solution, or final dilution for infusion is not clear or appears to have precipitation, these should be discarded. The final docetaxel dilution for infusion should be administered intravenously as per protocol under ambient room temperature and lighting conditions. Contact of the docetaxel concentrate with plasticized PVC equipment or

devices used to prepare solutions for infusion is not recommended. In order to minimize patient exposure to the plasticizer DEHP (di-2-ethylhexyl phthalate), which may be leached from PVC infusion bags or sets, the final docetaxel dilution for infusion should be stored in bottles (glass,

polypropylene) or plastic bags (polypropylene, polyolefin) and administered through polyethylene-lined administration sets.

Route of Administration:

Docetaxel will be administered as a 60 minute infusion in saline or D5W through an administration set that does not contain phthalate plasticizers along the fluid pathway that is connected to the patient's vascular access catheter.

Incompatibilities:

Contact of the undiluted concentrate with plasticized PVC equipment or devices used to prepare solutions for infusion should be avoided. Diluted docetaxel solution should be stored in bottles (glass, polypropylene) or plastic bags (polypropylene, polyolefin) and administered through polyethylene-lined administration sets. The metabolism of docetaxel may be modified by the concomitant administration of compounds that induce, inhibit, or are metabolized by cytochrome P450 3A4, such as cyclosporine, terfenadine, ketoconazole, erythromycin, and troleandomycin. Caution should be exercised with these drugs when treating patients receiving docetaxel as there is a potential for a significant interaction.

Availability:

Docetaxel (Taxotere®) is a commercial drug. The combination of docetaxel, cisplatin, and 5-FU for the treatment of patients with SCCHN is approved by the FDA and exempt from the requirements of an IND as described under Title 21 CFR 312.2(b).

Docetaxel vials of 80 mg in 2 ml polysorbate 80 and 20mg in 0.5ml polysorbate 80 with accompanying diluent (13% w/w ethanol in Water for Injection) are commercially available from Sanofi Pharmaceuticals. (The vials contain 15% overfill to compensate for liquid lost during

preparation). Docetaxel for Injection Concentrate is supplied in a single-dose vial as a sterile, pyrogen-free, non-aqueous, viscous solution with an accompanying sterile, nonpyrogenic, diluent (13% ethanol in Water for Injection) vial. The following strengths are available:

TAXOTERE 80 mg (NDC 0075-8001-80)

TAXOTERE (docetaxel) 80 mg Concentrate for Infusion: 80 mg docetaxel in 2 mL polysorbate 80 and diluent for TAXOTERE 80 mg. 13% (w/w) ethanol in Water for Injection. Both items are in a blister pack in one carton.

TAXOTERE 20 mg (NDC 0075-8001-20)

TAXOTERE (docetaxel) 20 mg Concentrate for Infusion: 20 mg docetaxel in 0.5 mL polysorbate 80 and diluent for TAXOTERE 20 mg. 13% (w/w) ethanol in Water for Injection. Both items are in a blister pack in one carton.

Nursing/Patient Implications:

1. Monitor CBC with differential and platelet count prior to drug administration.

2. Symptom management of expected nausea, vomiting, and mucositis.

3. Advise patients of possible hair loss.

4. Patients should be observed closely for hypersensitivity reactions, especially during the first and second infusions. Insure that recommended premedications are given.

5. Resuscitation equipment and medications to treat hypersensitivity reactions should be available during docetaxel administration.

6. Monitor liver function tests.

7. Evaluate site regularly for signs of infiltration.

8. Monitor for symptoms and signs of fluid retention, peripheral neuropathy, and cutaneous reactions.

8.2.2 Cisplatin- (Commercially available. Please refer to the package insert for further information)

Other Names Cis-diaminedichloroplatinum Cis-diaminedichloroplatinum (II), diaminedichloroplatinum,cis-platinum, platinum, Platinol®, Platinol-AQ®, DDP, CDDP, DACP, NSC 119875. Classification: Alkylating agent.

Mode of Action:

Inhibits DNA synthesis by forming inter- and intra-strand crosslinks. Other possible mechanisms include chelation of DNA and binding to cell membranes thereby stimulating immune mechanisms.

Storage and Stability:

Intact vials of cisplatin are stored at room temperature. Solutions diluted with sodium chloride or dextrose are stable for up to 72 hours at room temperature. Due to the risk of precipitation, cisplatin solutions should **not** be refrigerated.

Preparation:

The desired dose of cisplatin is diluted with 250 - 1000 ml of saline and/or dextrose solution. Varying concentrations of 0.225 - 5% sodium chloride and 5% dextrose may be used. To maintain stability of cisplatin, a final sodium chloride concentration of at least 0.2% is recommended.

Route of Administration:

Cisplatin should be administered as a 1 mg/ml intravenous infusion over 1-3 hours. Antiemetics should be given in conjunction with Cisplatin. Cisplatin is highly emetogenic. A suggested regimen is aprepitant 125 mg po on day 1 and 80 mg po on days 2 and 3 plus ondansetron (8 mg mg IV or 24 mg PO) or granisetron (1 mg IV or 2 mg PO) plus dexamethasone 12 mg po on day 1, 8 mg po on days 2-4) [83]

Metochlopramide 20-40 mg 2-4 times daily is suggested for patients with delayed nausea. Other antidopaminergic agents such as haloperidol can be used in patients with refractory symptoms.

Incompatibilities:

Amsacrine, cefepime, gallium nitrate, mesna, piperacillin, sodium bicarbonate, thiotepa. Cisplatin may react with aluminum which is found in some syringe needles or IV sets, forming a black precipitate.

Compatibilities:

Admixture: Amphotericin-B, aztreonam, carmustine, cefazolin, cephalothin, droperidol, etoposide, floxuridine, hydroxyzine, ifosphamide, leucovorin, magnesium sulfate, mannitol, potassium chloride.

Y-site: Allopurinol, bleomycin chlorpromazine, cimetidine, cyclophosphamide, dexamethasone, diphenhydramine, doxapram, doxorubicin, famotidine, filgrastim, fludarabine, fluorouracil, furosemide, ganciclovir, heparin, hydromorphone, lorazepam, melphalan, methotrexate, methylprednisolone, metoclopramide, mitomycin, morphine, ondansetron, paclitaxel, prochlorperazine, ranitidine, sargramostim, vinblastine, vincristine, vinorelbine.

Consult your pharmacist regarding specific concentrations.

Availability:

Commercially available as a mg/ml solution in 50 and 100 mg vials. Vials of lyophilized powder are no longer commercially available, but may be obtained directly from the manufacturer for chemoembolization use.

Nursing Implications:

1. Assess labs prior to administration (esp. CBC, platelet count, Cr).

2. Assess urine output prior to each dose. Maintain hydration. Urine output should be 500-150 ml/hr. Diuretics may be ordered.

3. Administer antiemetics before cisplatin, then q 2-4 h for 3-5 doses.

4. Observe carefully for signs of anaphylaxis.

5. Monitor for signs of neurotoxicity, hearing loss.

8.2.3 Carboplatin - (Commercially available. Please refer to the package insert for further information) Other Names: CBDCA, Paraplatin, JM-8, NSC 241240.

Classification: Second generation tetravalent organic platinum compound.

Mode of Action:

Like cisplatin, carboplatin produces predominately interstrand DNA crosslinks rather than DNA-protein crosslinks. Cell-cycle nonspecific.

Storage and Stability:

Intact vials are stored at room temperature and protected from light. The reconstituted solution is stable for at least 24 hours. When further diluted in glass or polyvinyl plastic to a concentration of 500 mg/ml, solutions have the following stability: in normal saline, 8 hours at 25°C; in 5% dextrose (when reconstituted in sterile water), 24 hours at 5 of 25°C.

Preparation:

Add 5, 15, or 45 ml sterile water, normal saline, or 5% dextrose to the 50, 150 or 450 mg vial, respectively. The resulting solution contains 10 mg/ml. The desired dose is further diluted, usually in 5% dextrose.

Administration:

Administer as a 30 minute infusion

Incompatibilities:

Forms a precipitate when in contact with aluminum.

Compatibilities:

Carboplatin (0.3 mg/ml) and etoposide (0.4 mg/ml) are chemically compatible in normal saline or 5% dextrose for 24 hours at room temperature.

Availability:

Commercially available in 50, 150, and 450 mg vials.

8.312 Nursing Implications

1. Monitor CBC and platelet count; nadir occurs at approximately day 21 with recovery by day 28-30.

2. Premedicate with antiemetics – evaluate effectiveness.

3. Monitor fluid status – maintain adequate hydration.

4. Assess skin/mucous membranes.

5. Assess for signs of peripheral neuropathy – coordination, sensory loss.

8.2.4 5-Fluorouracil- (Commercially available. Please refer to the package insert for further information).

Other Names

5-FU, Adrucil, Efudex.

Formulation

Available in 500 mg/10 mL ampules and vials, and 1 gm/ 20 ml. For further information, see package insert.

Administration: 5- Fluorouracil will be administered as a continuous IV infusion during induction chemotherapy following the completion of bevacizumab and docetaxel administration. 5-**Fluorouracil may be begin concurrently** with the cisplatin or carboplatin infusion. [31]

Drug Interactions

Cimetidine: Because cimetidine can decrease the clearance of 5-FU, patients should not enter on this study until the cimetidine is discontinued. Ranitidine or a drug from another anti-ulcer class can be substituted for cimetidine, as necessary.

Allopurinol: Oxypurinol, a metabolite of allopurinol, can potentially interfere with 5-FU anabolism via orotate phosphoribosyltransferase. Although this was originally used as a strategy to protect normal tissues from 5-FU-associated toxicity, further laboratory studies suggested possible antagonism of the anticancer activity of 5-FU in some tumor models. If a patient is receiving allopurinol, the need for taking this medicine should be ascertained. If possible, allopurinol should be discontinued prior to starting on this regimen, and another agent substituted for it.

Storage

Stable for prolonged periods of time at room temperature, if protected from light. Inspect for precipitate; if apparent, agitate vial vigorously or gently heat to not greater than 140°F in a water bath. Do not allow to freeze.

9. CORRELATIVE/SPECIAL STUDIES

9.1 Hypoxia Imaging

For a scientific background and rationale for noninvasive hypoxia imaging in NPC, please see the background sections of this protocol . Our hypothesis is that bevacizumab alone and TPFB will reduce intratumoral hypoxia prior to the administration of definitive concurrent chemoradiation and that this reduced hypoxia prior to chemoradiation may render the radiation treatment more effective. The purpose correlative imaging portion of this study is to preliminarily explore whether the first part of this hypothesis is true, That is, does one dose of bevacizumab alone and/ or induction TPFB reduce intratumoral hypoxia using non- invasive FMISO PET Scans as the assay for intratumoral hypoxia . An exploratory hypothesis generating correlation will be made between hypoxia reduction and clinical outcome in this small study.

FMISO agent description and background

FMISO is composed of ≤ 15 μg of fluoromisonidazole labeled with ≤ 10 mCi of radioactive ^{18}F at a specific activity >1 Ci/mg at the time of injection. The drug is the only active ingredient and it is formulated in ≤ 10 mL of 5% ethanol in saline for intravenous injection and has a half-life of 110 minutes.

Cardinal Health will manufacture and supply FMISO.

No adverse events have been reported with administration of FMISO in ~ 300 patients studied at the University of Washington.

Unexpected, but potential adverse effects with administration of FMISO include: infection at the injection site or systemic infection, extravasation of the dose, allergic reaction, secondary cancer from radiation exposure, death, peripheral neuropathy, nausea, vomiting, and anorexia.

Stanford will cross file under the NCI' s IND for use of FMISO

Functional imaging of hypoxia with PET scans has been evaluated in a number of clinical trials and has advantages over the measurement of oxygen content via oxygen electrodes. [84]PET imaging is less prone to the sampling artifact inherent in the placement of oxygen electrodes and PET, unlike invasive oxygen electrode evaluation, can distinguish more reliably between necrotic tumor regions and viable hypoxic tumor regions. The most widely used tracer for evaluating tumor hypoxia using PET has been [18F]-fluoromisonidazole, 1H-1-(3-[18F]-fluoro-2-hydroxy-propyl)-2-nitro-imidazole. (F-MISO). -18 EF5, I-124 IAZA and F-18 FAZA are other noninvasive hypoxia imaging agents in clinical development. [85 86]

F-MISO binds covalently to cellular molecules at rates that are inversely proportional to intracellular oxygen concentration. F-MISO is trapped in hypoxic cells, thereby enabling the measurement of hypoxia. F-MISO has been used in human subjects (approximately 300) in many centers throughout the world without reported serious adverse effects. The structure of FMISO is shown below:

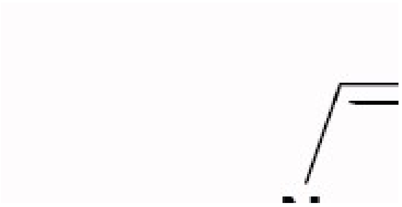

FMISO is a positron emitter with a half life of 110 minutes. Intravenously injected FMISO distributes throughout the total body water space, crossing cell membranes, including the blood brain barrier by passive diffusion. FMISO is bound and retained within viable hypoxic cells in an inverse relationship to the O_2 concentration.

FMISO dosimetry studies were performed at the University of Washington. Calculated total body dose for a 70 kg man injected with 3.7 MBq/kg was 0.013 mGy/MBq; for a 57 Kg woman it was 0.016 mGy/MBq. Effective dose equivalents were 0.013 mSv/MBq for men and 0.014 mSv/MBq for women. Ninety-seven percent of the injected radiation was homogenously distributed in the body, leaving only 3% for urinary excretion. The radiation exposures from FMISO was found to be equal to or lower than from other widely used nuclear medicine studies. Potential radiation risks associated with this study are within generally accepted limits .[87]

The University of Washington group has proposed the use of tumor to blood ratios as a reproducible method to quantify FMISO PET results whereby FMISO PET scans are presented as tissue-to-blood ratio images. A tumor to blood ratio less than 1.2 is considered to represent "hypoxia".

FMISO PET imaging for the TPFB protocol:

Patients will have FMISO PET imaging performed within the 2 weeks prior to the first bevacizumab dose, 5 to 8 days after the bevacizumab dose, and 2 to 4 weeks after the last TPFB dose, prior to initiation of radiation treatment. FMISO dose activity = 0.1mCi/kg (not to exceed 10 mCi)

Analysis of FMISO PET imaging results:

Hypoxia will be defined as a tissue to blood FMISO detection ratio of 1.2. Tumor regions of interest will be established using the FMISO PET data fused with CT data for attenuation correction. A functional hypoxic volume of all RECIST evaluable tumor regions (primary site and lymph nodes) will be calculated, based on the number of voxels with a tumor to blood ratio of 1.2 or greater. The relative hypoxic volumes at baseline, after bevacizumab, and after TPFB will be compared both quantitatively and qualitatively using descriptive statistics.

A FMISO PET imaging manual containing technical aspects of performance and analysis of FMISO PET studies is in development and will be APPENDIX C of this protocol.

Laboratory Correlative Studies

9.2 Plasma EBV DNA levels

9.2.1

3.5 mL of blood will be collected into EDTA anticoagulated tubes within 2 weeks prior to the first dose of bevacizumab, 5-8 days post the first dose of bevacizumab, and 2-4 weeks after the final dose of TPFB, but prior to the initiation of radiation, and 1-3 months after the completion of radiation.

Specimens will be labeled as follows:

Each specimen will be labeled with the anonymized Oncore subject number (generated on the date of enrollment) and the date of collection.

9.2.2

Blood collected at Stanford Cancer Center:

Transport the same day at room temperature to the Le lab (CCSR, South, 269 Campus Dr, R. 1230, Stanford, CA 94305-5152), centrifuge for 10 minutes at 1600g then 10 minutes at 16000g, recover plasma, and store at –20 or -70 degrees C until processing .

Blood collected at other sites:

Within 24 hours of collection, centrifuge for 10 minutes at 1600g then 10 minutes at 16000g, recover plasma, and store at -20 or –70 degrees C until processing. Shipping to Le lab at the following address:

Hongbing Cao, CCSR South, 269 Campus Dr, R. 1230, MC 5152, Stanford, CA 94305-5251

1.Ship the plasma via FEDEX on ICE with appropriate biohazard protection

2.Please contact Hongbin Cao at hcao17@stanford.edu for FEDEX shipping lable prior to shipping.

3. Call Hongbin Cal 650-725-7805 or email her at hcao17@stanford.edu prior to shipping to coordinate shipping and receipt of specimens. Please also cc Dr. Le at qle@stanford.edu.

9.2.3 Data management:

Upon receipt of specimens, designated Le laboratory personnel will notify the study coordinator to facilitate tabulation of all collected specimens, with data storage of this tabulation in Oncore.

9.2.4 Plasma EBV DNA assays will be done using the Pol-1 and the BamH1 probe and methodology as previously described by the Le lab. [4]

9.2.5 Statistical analysis of EBV DNA results:

Kaplan-Meier methods will be used to analyze the relationship of baseline, post bevacizumab, post TPFB, and post radiation EBV DNA levels to overall response, freedom-from-relapse and overall survival.

10. STUDY CALENDAR

NOTE: RED HIGHLIGHTED ITEMS ARE RESEARCH, NOT STANDARD OF CARE

Baseline evaluations are to be conducted within 1 week prior to start of protocol therapy. Scans and x-rays must be done within 1 month prior to the start of therapy. In the event that the patient's condition is deteriorating, laboratory evaluations should be repeated within 48 hours prior to initiation of the next cycle of therapy.

Induction Chemotherapy with TPFB study calendar (separate calendar for radiation portion and post radiation follow-up is below)

	**Pre study**	Wk 1	Wk 2	Wk 3	Wk 4	Wk 5	Wk 6	Wk 7	Wk 8	Wk 9	**End of induction**
Bevacizumab		**A** #			**A**			**A**			
TPF		**B**			**B**			**B**			
Informed consent	**x**										
Demographics	**x**										
Medical history	**x**										
Concurrent meds	**x**	**x**			**x**			**x**			**x**
Physical exam	**x**	**x**			**x**			**x**			**x**
Vital signs	**x**	**x**			**x**			**x**			**x**
Height	**x**										
Weight	**x**										
Performance status	**x**										
CBC w/diff, plts[d]	**x**	**x**			**x**			**x**			**x**
Comp. metab. profile [a,d]	**x**	**x**			**x**			**x**			**x**
aPTT , PT/INR	**x**										
EKG (as indicated)	**x**										
Adverse event evaluation	**x**	**x**			**x**			**x**			**x**

Tumor measurements per clinical routine	**x**	**x**			**x**			**x**			**x**
Radiological evaluation [f]	**x**										**x**
Urine analysis for UPC ratio[b]	**x**				**x**						**x**
**FMISO PET imaging** [c]	**C** [c]	**C** [c]									**C**
**Plasma EBV DNA** [c]	**x** [c]	**x** [c]									**x**

A#: Bevacizumab, 15 mg/kg on day 1 of each cycle except cycle , when bevacizumab will be administered separately, 1 week prior to TPF.

B: TPF: *Docetaxel 75 mg/m2 and cisplatin 75 mg/m2 on day 1 of each cycle. 5-FU, 750 mg/m2 on days 1,2,3,4,5 of each cycle as IVCI,*

a: Albumin, alkaline phosphatase, total bilirubin, bicarbonate, BUN, calcium, chloride, creatinine, glucose, potassium, total protein, SGOT [AST], SGPT [ALT], sodium.

b: Urine analysis for calculation of Urine Protein: Creatinine Ratio (**UPC ratio**) should be performed prior to each or every other course of bevacizumab. If UPC ration is $\geq$ 1, collection of 24 hour urine for measurement of urine protein level is recommended but not required.

UPC ratio of spot urine is an estimation of the 24 urine protein excretion – a UPC ratio of 1 is roughly equivalent to a 24-hour urine protein of 1 gm. UPC ratio is calculated using one of the following formulas:

- [urine protein]/[urine creatinine] – if both protein and creatinine are reported in mg/dL
- [(urine protein) x0.088]/[urine creatinine] – if urine creatinine is reported in mmol/L

c: FMISO PET imaging and plasma EBV DNA is to be within 2 weeks prior to first dose of bevacizumab, 5-7 days post the first dose of bevacizumab and prior to TPF, and 2-4 weeks after the last dose of TPFB

d: While the protocol mandates a blood tests only prior to each dose of TPFB, good clinical practice should be used in ordering additional lab tests as part of routine care for patients receiving chemotherapy.

e: Basic metabolic panel: calcium, CO2, chloride, creatinine, glucose, potassium, sodium, BUN

f: Radiological evaluation will be tailored to the patient with the following parameters: There must be CT or MRI evaluation of the primary site and neck, and a baseline evaluation for metastatic disease to include at minimum chest CT or total body FDG PET scan. Imaging modality should be consistent for each patient throughout.

Concurrent chemoradiation study calendar (separate calendar for induction TPFB is above, use same footnotes)

	Induct . End (from above)	Wk1	Wk2	Wk3	Wk4	Wk5	Wk6	Wk7	1 mo f/u	3,6,9, 12 , 24 mo f/u
Radiation treatment		x	x	x	x	x	x			
Bevacizumab		x			x			x		
Cisplatin(or carboplatin)		x	x	x	x	x	x			
Concurrent meds	x	x	x	x	x	x	x	x	x	x
Physical exam	x	x	x	x	x	x	x	x	x	x
Vital signs	x									
Performance status	x								x	x
CBC	x	x	x	x	x	x	x	x	x	
Basic metabolic profile [e]		x	x	x		x	x			
Comp. metab. Profile [a]	x				x			x	x	
AE evaluation	x	x	x	x	x	x	x	x	x	X
Urine UPC [b]	x				x			x		
**Plasma EBV DNA** [c]	x									X*
**FMISO PET imaging** [c]	x									
Tumor measure per clinical routine	x								x	X
Radiological evaluation [f]	x									X#

*= only at 3 monthly followup
only at 3 , 12, and 24 month followup

11. MEASUREMENT OF EFFECT

11.1 **Antitumor Effect – Solid Tumors**

For the purposes of this study, patients should be reevaluated for response after TPFB and 3,6 ,9 ,12 and 24 months after the completion of radiation. The primary endpoint of response assessment will be based on MRI and or CT imaging and physical exam using the RECIST criteria (see below).While other modalities for response assessment (e.g. PET scanning, serum tumor markers) will be collected and may be used for clinical planning, they will not be used to evaluate the primary endpoint.

Response and progression will be evaluated in this study using the new international criteria proposed by the Response Evaluation Criteria in Solid Tumors (RECIST) Committee [*JNCI* 92(3):205-216, 2000]. Changes in only the largest diameter (unidimensional measurement) of the tumor lesions are used in the RECIST criteria.

11.1.1. **Definitions**

Evaluable for toxicity. All patients will be evaluable for toxicity from the time of their first treatment with bevacizumab

Evaluable for objective response. Only those patients who have measurable disease present at baseline, have received at least one cycle of TPFB, and have had their disease re-evaluated will be considered evaluable for response.

11.1.2 **Disease Parameters**

Measurable disease. Measurable lesions are defined as those that can be accurately measured in at least one dimension (longest diameter to be recorded) as ≥20 mm with conventional techniques (CT, MRI, x-ray) or as ≥10 mm with spiral CT scan. All tumor measurements must be recorded in millimeters (or decimal fractions of centimeters).

Non-measurable disease. All other lesions (or sites of disease), including small lesions (longest diameter <20 mm with conventional techniques or <10 mm using spiral CT scan), are considered non-measurable disease. Bone lesions, leptomeningeal disease, ascites, pleural/pericardial effusions, lymphangitis cutis/pulmonis, inflammatory breast disease, abdominal masses (not followed by CT or MRI), and cystic lesions are all non-measurable.

Target lesions. All measurable lesions up to a maximum of 5 lesions per organ and 10 lesions in total, representative of all involved organs, should be identified as **target lesions** and recorded and measured at baseline. Target lesions should be selected on the basis of their size (lesions with the longest diameter) and their suitability for accurate repeated measurements (either by imaging techniques or clinically). A sum of the longest diameter (LD) for all target lesions will be calculated and reported as the baseline sum LD. The baseline sum LD will be used as reference by which to characterize the objective tumor response.

Non-target lesions. All other lesions (or sites of disease) including any measurable lesions over and above the 10 target lesions should be identified as **non-target lesions** and should also be recorded at baseline. Measurements of these lesions are not required, but the presence or absence of each should be noted throughout follow-up.

11.1.3 **Methods for Evaluation of Measurable Disease**

All measurements should be taken and recorded in metric notation using a ruler or calipers. All baseline evaluations should be performed as closely as possible to the beginning of treatment and never more than 4 weeks before the beginning of the treatment.

The same method of assessment and the same technique should be used to characterize each identified and reported lesion at baseline and during follow-up. Imaging-based evaluation is preferred to evaluation by clinical examination when both methods have been used to assess the antitumor effect of a treatment.

Clinical lesions Clinical lesions will only be considered measurable when they are superficial (e.g., skin nodules and palpable lymph nodes). In the case of skin lesions, documentation by color photography, including a ruler to estimate the size of the lesion, is recommended.

Chest x-ray Lesions on chest x-ray are acceptable as measurable lesions when they are clearly defined and surrounded by aerated lung. However, CT is preferable.

Conventional CT and MRI These techniques should be performed with cuts of 10 mm or less in slice thickness contiguously. Spiral CT should be performed using a 5 mm contiguous reconstruction algorithm. This applies to tumors of the chest, abdomen, and pelvis. Head and neck tumors and those of extremities usually require specific protocols.

Ultrasound (US) When the primary endpoint of the study is objective response

evaluation, US should not be used to measure tumor lesions. It is, however, a possible alternative to clinical measurements of superficial palpable lymph nodes, subcutaneous lesions, and thyroid nodules. US might also be useful to confirm the complete disappearance of superficial lesions usually assessed by clinical examination.

Endoscopy, Laparoscopy The utilization of these techniques for objective tumor evaluation has not yet been fully and widely validated. Their uses in this specific context require sophisticated equipment and a high level of expertise that may only be available in some centers. Therefore, the utilization of such techniques for objective tumor response should be restricted to validation purposes in reference centers. However, such techniques may be useful to confirm complete pathological response when biopsies are obtained.

Tumor markers Tumor markers alone cannot be used to assess response. If markers are initially above the upper normal limit, they must normalize for a patient to be considered in complete clinical response. Specific additional criteria for standardized usage of prostate-specific antigen (PSA) and CA-125 response in support of clinical trials are being developed.

Cytology, Histology These techniques can be used to differentiate between partial responses (PR) and complete responses (CR) in rare cases (e.g., residual lesions in tumor types, such as germ cell tumors, where known residual benign tumors can remain).

The cytological confirmation of the neoplastic origin of any effusion that appears or worsens during treatment when the measurable tumor has met criteria for response or stable disease is mandatory to differentiate between response or stable disease (an effusion may be a side effect of the treatment) and progressive disease.

11.1.4 **Response Criteria**

11.1.4.1 **Evaluation of Target Lesions**

Complete Response (CR): Disappearance of all target lesions

Partial Response (PR): At least a 30% decrease in the sum of the longest diameter (LD) of target lesions, taking as reference the baseline sum LD

Progressive Disease (PD): At least a 20% increase in the sum of the LD of target lesions, taking as reference the smallest sum LD recorded since the treatment started or the appearance of one or more new lesions

Stable Disease (SD): Neither sufficient shrinkage to qualify for PR nor sufficient increase to qualify for PD, taking as reference the smallest sum LD since the treatment started

11.1.4.2 **Evaluation of Non-Target Lesions**

Complete Response (CR): Disappearance of all non-target lesions and normalization of tumor marker level

Note: If tumor markers are initially above the upper normal limit, they must normalize for a patient to be considered in complete clinical response.

Incomplete Response/

Stable Disease (SD): Persistence of one or more non-target lesion(s) and/or maintenance of tumor marker level above the normal limits

Progressive Disease (PD): Appearance of one or more new lesions and/or unequivocal progression of existing non-target lesions

Although a clear progression of "non-target" lesions only is exceptional, the opinion of the treating physician should prevail in such circumstances, and the progression status should be confirmed at a later time by the review panel (or Principal Investigator).

11.1.4.3 **Evaluation of Best Overall Response**

The best overall response is the best response recorded from the start of the treatment until disease progression/recurrence (taking as reference for progressive disease the smallest measurements recorded since the treatment started). The patient's best response assignment will depend on the achievement of both measurement and confirmation criteria

<table>
<tr><th>Target Lesions</th><th>Non-Target Lesions</th><th>New Lesions</th><th>Overall Response</th><th>Best Response following radiation for this Category Also Requires:</th></tr>
<tr><td>CR</td><td>CR</td><td>No</td><td>CR</td><td>≥4 wks. confirmation</td></tr>
<tr><td>CR</td><td>Non-CR/Non-PD</td><td>No</td><td>PR</td><td rowspan="2">≥4 wks. confirmation</td></tr>
<tr><td>PR</td><td>Non-PD</td><td>No</td><td>PR</td></tr>
<tr><td>SD</td><td>Non-PD</td><td>No</td><td>SD</td><td>documented at least once ≥4 wks. from baseline</td></tr>
<tr><td>PD</td><td>Any</td><td>Yes or No</td><td>PD</td><td rowspan="3">no prior SD, PR or CR</td></tr>
<tr><td>Any</td><td>PD*</td><td>Yes or No</td><td>PD</td></tr>
<tr><td>Any</td><td>Any</td><td>Yes</td><td>PD</td></tr>
</table>

* In exceptional circumstances, unequivocal progression in non-target lesions may be accepted as disease progression.

Note: Patients with a global deterioration of health status requiring discontinuation of treatment without objective evidence of disease progression at that time should be reported as "*symptomatic deterioration*". Every effort should be made to document the objective progression even after discontinuation of treatment.

11.1.5 **Duration of Response**

Duration of overall response: The duration of overall response is measured from the time measurement criteria are met for CR or PR (whichever is first recorded) until the first date that recurrent or progressive disease is objectively documented (taking as reference for progressive disease the smallest measurements recorded since the treatment started).

The duration of overall CR is measured from the time measurement criteria are first met for CR until the first date that recurrent disease is objectively documented.

Duration of stable disease: Stable disease is measured from the start of the treatment until the criteria for progression are met, taking as reference the smallest measurements recorded since the treatment started.

11.1.6 **Progression-Free Survival**

Progression – free survival will be calculated as the interval between date of registration and date of documented cancer progression or death, whichever occurs first.

11.1.7 **Response Review**

All radiological images used for response assessment must be available to the principal investigator for response assessment review. For patients imaged at Stanford Cancer center/ Stanford Hospital, images available within the Hospital radiology system fulfill this criterion. For images acquired outside of the Stanford system, images must be transferred to a separate , portable medium (i.e. CD or DVD), labeled with the patient's anonymized Oncore protocol registration number and scan date, and sent to the clinical trial coordinator at the address on the first page of this protocol.

12. DATA REPORTING / REGULATORY REQUIREMENTS

Adverse event lists, guidelines, and instructions for AE reporting can be found in Section 7.0 (Adverse Events: List and Reporting Requirements).

12.1 **Data Reporting**

12.1.1 Method

This study will be monitored by the Clinical Data Update System (CDUS) version 3.0. Cumulative CDUS data will be submitted quarterly to CTEP by electronic means. **Reports to CTEP are due January 31, April 30, July 31, and October 31**. Instructions for submitting data using the CDUS can be found on the CTEP web site (http://ctep.cancer.gov). **Note**: **All** adverse events that have occurred on the study, including those reported through AdEERS, must be reported via CDUS.

12.1.2 Responsibility for Data Submission

Study participants are responsible for submitting CDUS data and/or data forms **to the Coordinating Center quarterly by January 1, April 1, July 1, and October 1** to allow time for Coordinating Center compilation, Principal Investigator review, and timely submission to CTEP (see Section 12.1.1

The Coordinating Center is responsible for compiling and submitting CDUS data to CTEP for all participants and for providing the data to the Principal Investigator for review.

12.2 CTEP Multicenter Guidelines

This protocol will adhere to the policies and requirements of the CTEP Multicenter Guidelines. The specific responsibilities of the Principal Investigator and the Coordinating Center (Study Coordinator) and the procedures for auditing are presented in Appendix D.

- The Principal Investigator/Coordinating Center is responsible for distributing all IND Action Letters or Safety Reports received from CTEP to all participating institutions for submission to their individual IRBs for action as required.
- Except in very unusual circumstances, each participating institution will order DCTD-supplied agents directly from CTEP. Agents may be ordered by a participating site only after the initial IRB approval for the site has been forwarded by the Coordinating Center to the CTEP PIO (*PIO@ctep.nci.nih.gov*) .

12.3 Cooperative Research and Development Agreement (CRADA)/Clinical Trials Agreement (CTA)

The agent(s) supplied by CTEP, DCTD, NCI used in this protocol is/are provided to the NCI under a Collaborative Agreement (CRADA, CTA, CSA) between the Pharmaceutical Company(ies) (hereinafter referred to as Collaborator(s) and the NCI Division of Cancer Treatment and Diagnosis. Therefore, the following obligations/guidelines, in addition to the provisions in the Intellectual Property Option to Collaborator (http:// ctep.cancer.gov/industry) contained within the terms of award, apply to the use of the Agent(s) in this study:

1. Agent(s) may not be used for any purpose outside the scope of this protocol, nor can Agent(s) be transferred or licensed to any party not participating in the clinical study. Collaborator(s) data for Agent(s) are confidential and proprietary to Collaborator(s) and shall be maintained as such by the investigators. The protocol documents for studies utilizing investigational Agents contain confidential information and should not be shared or distributed without the permission of the NCI. If a copy of this protocol is requested by a patient or patient's family member participating on the study, the individual should sign a confidentiality agreement. A suitable model agreement can be downloaded from: http://ctep.cancer.gov.

2. For a clinical protocol where there is an investigational Agent used in combination with (an)other investigational Agent(s), each the subject of different collaborative agreements , the access to and use of data by each Collaborator shall be as follows (data pertaining to such combination use shall hereinafter be referred to as "Multi-Party Data"):

a. NCI will provide all Collaborators with prior written notice regarding the existence and nature of any agreements governing their collaboration with NIH, the design of the proposed combination protocol, and the existence of any obligations that would tend to restrict NCI's participation in the proposed combination protocol.

b. Each Collaborator shall agree to permit use of the Multi-Party Data from the clinical trial by any other Collaborator solely to the extent necessary to allow said other Collaborator to develop, obtain regulatory approval or commercialize its own investigational Agent.

c. Any Collaborator having the right to use the Multi-Party Data from these trials must agree in writing prior to the commencement of the trials that it will use the Multi-Party Data solely for development, regulatory approval, and commercialization of its own investigational Agent.

3. Clinical Trial Data and Results and Raw Data developed under a Collaborative Agreement will be made available exclusively to Collaborator(s), the NCI, and the FDA, as appropriate and unless additional disclosure is required by law or court order. Additionally, all Clinical Data and Results and Raw Data will be collected, used, and disclosed consistent with all applicable federal statutes and regulations for the protection of human subjects including, if applicable, the *Standards for Privacy of Individually Identifiable Health Information* set forth in 45 C.F.R. Part 164.

4. When a Collaborator wishes to initiate a data request, the request should first be sent to the NCI, who will then notify the appropriate investigators (Group Chair for Cooperative Group studies, or PI for other studies) of Collaborator's wish to contact them.

5. Any data provided to Collaborator(s) for Phase 3 studies must be in accordance with the guidelines and policies of the responsible Data Monitoring Committee (DMC), if there is a DMC for this clinical trial.

6. Any manuscripts reporting the results of this clinical trial must be provided to CTEP by the principal investigator for immediate delivery to Collaborator(s) for advisory review and comment prior to submission for publication. Collaborator(s) will have 30 days from the date of receipt for review. Collaborator shall have the right to request that publication be delayed for up to an additional 30 days in order to ensure that Collaborator's confidential and proprietary data, in addition to Collaborator(s)'s intellectual property rights, are protected. Copies of abstracts must be provided to CTEP for forwarding to Collaborator(s) for courtesy review as soon as possible and preferably at least three (3) days prior to submission, but in any case, prior to presentation at the meeting or publication in the proceedings. Press releases and other media presentations must also be forwarded to CTEP prior to release. Copies of any manuscript, abstract and/or press release/ media presentation should be sent to:

Regulatory Affairs Branch, CTEP, DCTD, NCI

Executive Plaza North, Suite 7111

Bethesda, Maryland 20892

FAX 301-402-1584

Email: anshers@mail.nih.gov

The Regulatory Affairs Branch will then distribute them to Collaborator(s). No publication, manuscript or other form of public disclosure shall contain any of Collaborator's confidential/ proprietary information.

13. STATISTICAL CONSIDERATIONS

13.1 Study Design/Endpoints

The primary endpoint for this trial will be progression free survival 2 years following chemoradiotherapy.

The US intergroup CR rate to chemoradiotherapy was 49%, using SWOG response criteria that did not include PET imaging. [5] However, many have argued that this CR rate was atypically low when compared to other studies conducted primarily in Asia .

Because the CR rates reported in many other studies are so high and because PFS rates at one year in many RCTS of chemoradiaton in NPC are so high, [5-7, 11] we have decided to make PFS at 2 years following the end of chemoradiation as the primary endpoint of this study. In the 4 studies referenced above, the average 2 year PFS for the superior arm was 0.7 and no single study achieved a PFS greater than 0.85. Therefore we will evaluate 40 patients in a single stage design for progression free survival at 2 years post radiation, which will give an alpha of 0.1 and a power of 0.9 to distinguish a 2 year PFS of 0.88 from 0.7 .

It is not feasible with the accrual rate planned to introduce an early stopping rule for a PFS at 2 years. Therefore, in order to avoid accruing up to 40 patients to a study that is unlikely to be of interest, we will have an early stopping rule based on RECIST determined CR rates. If there are less than 10 CRs in the first 18 patients, we would stop the trial. If the trial goes to the second stage, the treatment will be considered worthy of further study if 33 or more of the 40 patients are progression-free at 2 years.

13.2 **Sample Size/Accrual Rate**

18-40 evaluable patients. 2-4 per month. All patients who are not evaluable will be replaced. Any patient who starts treatment with TPFB will be considered evaluable for response.

13.3 **Stratification Factors**

No stratification

13.4 Analysis of Secondary Endpoints:

1. Progression free survival and overall survival will be estimated according to the methods of Kaplan and Meier.

1. Rates of adverse events will be analyzed as follows:

The acceptable incidence of AEs resulting in protocol treatment discontinuance is 3% or less, and the unacceptable rate is 15% or greater. The rates of AEs resulting in protocol treatment discontinuation will be estimated using a binomial distribution along with their associated 95% confidence intervals. Only adverse events assessed definitely, probably, or possibly related to protocol treatment will be considered. 40 evaluable patients will be able to distinguish between the above null and alternative hypothesis with and alpha error of .03 and power .87.

2. Statistical analysis of FMISO PET and EBV DNA results:

Logistic regression and Cox regression methods will be used to analyze the relationship of baseline, post bevacizumab, post TPFB, and post radiation EBV DNA levels to overall response, freedom-from-relapse and overall survival, using EBV DNA levels of zero versus nonzero. Exploratory analysis of correlations between changes in hypoxia by FMISO PET and CR rate, PFS and OS will be performed.

13.5 **Reporting**

13.5.1 **Evaluation of toxicity.** All patients will be evaluable for toxicity from the time of their first treatment with bevacizumab. . We are aware that there are no safety data for the combination of bevacizumab plus TPF proposed in this study. The ongoing RTOG 0615 study already has demonstrated that bevacizumab, added to concurrent cisplatin and radiation, is feasible. Therefore, in order to ensure that TPFB is safe without exposing an inordinate number of patients to an untested recipe, there will be a planned cumulative safety review of all patients on study after of 5 , 10, 15 and 20 patients have been treated with 3 cycles of TPFB. We will evaluate annually the cumulative rate and nature of all AEs, both acute and long term, as part of the standard Stanford IRB annual update requirement.. Should either the investigators or IRB decide that the cumulative AE rate is unacceptable, accrual would be halted until an IRB approved amendment addressing this situation is implemented.

13.5.2 **Evaluation of response.** All patients who receive one cycle of TPFB will be considered evaluable for response. Response categories will be: 1) complete response, 2) partial response, 3) stable disease, 4) progressive disease, 5) early death from malignant disease, 6) early death from toxicity, 7) early death because of other cause, or 9) unknown (not assessable, insufficient data).

REFERENCES

1.Licitra L, Bernier J, Cvitkovic E, et al. Cancer of the nasopharynx. Critical Reviews in Oncology-Hematology 2003;45:199-213.

2.Kantakamalakul W, Chongkolwatana C, Naksawat P, et al. Specific IgA antibody to Epstein-Barr viral capsid antigen: a better marker for screening nasopharyngeal carcinoma than EBV-DNA detection by polymerase chain reaction. Asian Pacific Journal of Allergy & Immunology 2000;18:221-6.

3.Leung SF, Zee B, Ma BB, et al. Plasma Epstein-Barr viral deoxyribonucleic acid quantitation complements tumor-node-metastasis staging prognostication in nasopharyngeal carcinoma. Journal of Clinical Oncology 2006;24:5414-8.

4.Le QT, Jones CD, Yau TK, et al. A comparison study of different PCR assays in measuring circulating plasma epstein-barr virus DNA levels in patients with nasopharyngeal carcinoma. Clinical Cancer Research 2005;11:5700-7.

5.Al-Sarraf M, LeBlanc M, Giri PG, et al. Chemoradiotherapy versus radiotherapy in patients with advanced nasopharyngeal cancer: phase III randomized Intergroup study 0099. Journal of Clinical Oncology 1998;16:1310-7.

6.Lin JC, Jan JS, Hsu CY, Liang WM, Jiang RS, Wang WY. Phase III study of concurrent chemoradiotherapy versus radiotherapy alone for advanced nasopharyngeal carcinoma: positive effect on overall and progression-free survival.[see comment]. Journal of Clinical Oncology 2003;21:631-7.

7.Chan AT, Leung SF, Ngan RK, et al. Overall survival after concurrent cisplatin-radiotherapy compared with radiotherapy alone in locoregionally advanced nasopharyngeal carcinoma. Journal of the National Cancer Institute 2005;97:536-9.

8.Lee AW, Tung SY, Chan AT, et al. Preliminary results of a randomized study (NPC-9902 Trial) on therapeutic gain by concurrent chemotherapy and/or accelerated fractionation for locally advanced nasopharyngeal carcinoma. International Journal of Radiation Oncology, Biology, Physics 2006;66:142-51.

9.Chan AT, Teo PM, Ngan RK, et al. Concurrent chemotherapy-radiotherapy compared with radiotherapy alone in locoregionally advanced nasopharyngeal carcinoma: progression-free survival analysis of a phase III randomized trial.[see comment]. Journal of Clinical Oncology 2002;20:2038-44.

10.Chua DT, Sham JS, Wei WI, Ho WK, Au G, Choy D. Control of regional metastasis after induction chemotherapy and radiotherapy for nasopharyngeal carcinoma. Head & Neck 2002;24:350-60.

11.Chua DT, Ma J, Sham JS, et al. Long-term survival after cisplatin-based induction chemotherapy and radiotherapy for nasopharyngeal carcinoma: a pooled data analysis of two phase III trials.[see comment]. Journal of Clinical Oncology 2005;23:1118-24.

12.Hong RL, Ting LL, Ko JY, et al. Induction chemotherapy with mitomycin, epirubicin, cisplatin, fluorouracil, and leucovorin followed by radiotherapy in the treatment of locoregionally advanced nasopharyngeal carcinoma. Journal of Clinical Oncology 2001;19:4305-13.

13.Paccagnella A, Favaretto A, Oniga F, et al. Cisplatin versus carboplatin in combination with mitomycin and vinblastine in advanced non small cell lung cancer. A multicenter, randomized phase III trial. Lung Cancer 2004;43:83-91.

14.Scagliotti GV, De Marinis F, Rinaldi M, et al. Phase III randomized trial comparing three platinum-based doublets in advanced non-small-cell lung cancer. Journal of Clinical Oncology 2002;20:4285-91.

15.Fossella F, Pereira JR, von Pawel J, et al. Randomized, multinational, phase III study of docetaxel plus platinum combinations versus vinorelbine plus cisplatin for advanced non-small-cell lung cancer: the TAX 326 study group.[see comment]. Journal of Clinical Oncology 2003;21:3016-24.

16.Aravantinos G, Fountzilas G, Kosmidis P, et al. Paclitaxel plus carboplatin versus paclitaxel plus alternating carboplatin and cisplatin for initial treatment of advanced ovarian cancer: long-term efficacy results: a Hellenic Cooperative Oncology Group (HeCOG) study. Annals of Oncology 2005;16:1116-22.

17.du Bois A, Luck HJ, Meier W, et al. A randomized clinical trial of cisplatin/paclitaxel versus carboplatin/paclitaxel as first-line treatment of ovarian cancer. Journal of the National Cancer Institute 2003;95:1320-9.

18.Ozols RF, Bundy BN, Greer BE, et al. Phase III trial of carboplatin and paclitaxel compared with cisplatin and paclitaxel in patients with optimally resected stage III ovarian cancer: a Gynecologic Oncology Group study.[see comment]. Journal of Clinical Oncology 2003;21:3194-200.

19.Homma A, Shirato H, Furuta Y, et al. Randomized phase II trial of concomitant chemoradiotherapy using weekly carboplatin or daily low-dose cisplatin for squamous cell carcinoma of the head and neck. Cancer Journal 2004;10:326-32.

20.Chitapanarux I, Lorvidhaya V, Kamnerdsupaphon P, et al. Chemoradiation comparing cisplatin versus carboplatin in locally advanced nasopharyngeal cancer: randomised, non-inferiority, open trial. European Journal of Cancer 2007;43:1399-406.

21.Forastiere AA, Shank D, Neuberg D, Taylor SGt, DeConti RC, Adams G. Final report of a phase II evaluation of paclitaxel in patients with advanced squamous cell carcinoma of the head and neck: an Eastern Cooperative Oncology Group trial (PA390). Cancer 1998;82:2270-4.

22.Dreyfuss AI, Clark JR, Norris CM, et al. Docetaxel: an active drug for squamous cell carcinoma of the head and neck. Journal of Clinical Oncology 1996;14:1672-8.

23.Hitt R, Grau J, Lopez- Puose A, et al. Randomized phase II/III clinical trial of induction chemotherapy (ICT) with either cisplatin/5-fluorouracil (PF) or docetaxel/cisplatin/5-fluorouracil (TPF) followed by chemoradiotherapy (CRT) vs. crt alone for patients (pts) with unresectable locally advanced head and neck cancer (LAHNC). Journal of Clinical Oncology 2006;24:5515.

24.Vermorken JB, Remenar E, van Herpen C, et al. Cisplatin, fluorouracil, and docetaxel in unresectable head and neck cancer. New England Journal of Medicine 2007;357:1695-704.

25.Posner MR, Colevas AD. Induction chemotherapy in the management of squamous

cell cancer of the head and neck.[comment]. Cancer Journal From Scientific American 1997;3:73-5.

26.Administration USFaD. Approval History New Drug Application # 020449 for docetaxel.

27.Pignon JP, Syz N, Posner M, et al. Adjusting for patient selection suggests the addition of docetaxel to 5-fluorouracil-cisplatin induction therapy may offer survival benefit in squamous cell cancer of the head and neck. Anti-Cancer Drugs 2004;15:331-40.

28.Chua DT, Sham JS, Au GK. A phase II study of docetaxel and cisplatin as first-line chemotherapy in patients with metastatic nasopharyngeal carcinoma. Oral Oncology 2005;41:589-95.

29.Haddad R, Colevas AD, Tishler R, et al. Docetaxel, cisplatin, and 5-fluorouracil-based induction chemotherapy in patients with locally advanced squamous cell carcinoma of the head and neck: the Dana Farber Cancer Institute experience. Cancer 2003;97:412-8.

30.Colevas AD, Busse PM, Norris CM, et al. Induction chemotherapy with docetaxel, cisplatin, fluorouracil, and leucovorin for squamous cell carcinoma of the head and neck: a phase I/II trial.[see comment]. Journal of Clinical Oncology 1998;16:1331-9.

31.Colevas AD, Norris CM, Tishler RB, et al. Phase II trial of docetaxel, cisplatin, fluorouracil, and leucovorin as induction for squamous cell carcinoma of the head and neck.[see comment]. Journal of Clinical Oncology 1999;17:3503-11.

32.Colevas AD, Norris CM, Tishler RB, et al. Phase I/II trial of outpatient docetaxel, cisplatin, 5-fluorouracil, leucovorin (opTPFL) as induction for squamous cell carcinoma of the head and neck (SCCHN). American Journal of Clinical Oncology 2002;25:153-9.

33.Kyzas PA, Cunha IW, Ioannidis JP. Prognostic significance of vascular endothelial growth factor immunohistochemical expression in head and neck squamous cell carcinoma: a meta-analysis. Clinical Cancer Research 2005;11:1434-40.

34.Wakisaka N, Wen QH, Yoshizaki T, et al. Association of vascular endothelial growth factor expression with angiogenesis and lymph node metastasis in nasopharyngeal carcinoma. Laryngoscope 1999;109:810-4.

35.Qian CN, Zhang CQ, Guo X, et al. Elevation of serum vascular endothelial growth factor in male patients with metastatic nasopharyngeal carcinoma. Cancer 2000;88:255-61.

36.Krishna SM, James S, Balaram P. Expression of VEGF as prognosticator in primary nasopharyngeal cancer and its relation to EBV status. Virus Research 2006;115:85-90.

37.Druzgal CH, Chen Z, Yeh NT, et al. A pilot study of longitudinal serum cytokine and angiogenesis factor levels as markers of therapeutic response and survival in patients with head and neck squamous cell carcinoma. Head & Neck 2005;27:771-84.

38.Sanguineti G, Geara FB, Garden AS, et al. Carcinoma of the nasopharynx treated by radiotherapy alone: determinants of local and regional control.[see comment]. International Journal of Radiation Oncology, Biology, Physics 1997;37:985-96.

39.Okunieff P, de Bie J, Dunphy EP, Terris DJ, Hockel M. Oxygen distributions partly

explain the radiation response of human squamous cell carcinomas. British Journal of Cancer - Supplement 1996;27:S185-90.

40.Jain RK. Normalization of tumor vasculature: an emerging concept in antiangiogenic therapy. Science 2005;307:58-62.

41.Winkler F, Kozin SV, Tong RT, et al. Kinetics of vascular normalization by VEGFR2 blockade governs brain tumor response to radiation: role of oxygenation, angiopoietin-1, and matrix metalloproteinases.[see comment]. Cancer Cell 2004;6:553-63.

42.Willett CG, Boucher Y, di Tomaso E, et al. Direct evidence that the VEGF-specific antibody bevacizumab has antivascular effects in human rectal cancer.[see comment][erratum appears in Nat Med. 2004 Jun;10(6):649]. Nature Medicine 2004;10:145-7.

43.Taghian AG, Abi-Raad R, Assaad SI, et al. Paclitaxel decreases the interstitial fluid pressure and improves oxygenation in breast cancers in patients treated with neoadjuvant chemotherapy: clinical implications. Journal of Clinical Oncology 2005;23:1951-61.

44.Batchelor TT, Sorensen AG, di Tomaso E, et al. AZD2171, a pan-VEGF receptor tyrosine kinase inhibitor, normalizes tumor vasculature and alleviates edema in glioblastoma patients.[see comment]. Cancer Cell 2007;11:83-95.

45.Yeh SH, Liu RS, Wu LC, et al. Fluorine-18 fluoromisonidazole tumour to muscle retention ratio for the detection of hypoxia in nasopharyngeal carcinoma. European Journal of Nuclear Medicine 1996;23:1378-83.

46.Leslie MD, Dische S. The early changes in salivary gland function during and after radiotherapy given for head and neck cancer. Radiotherapy & Oncology 1994;30:26-32.

47.Mira JG, Wescott WB, Starcke EN, Shannon IL. Some factors influencing salivary function when treating with radiotherapy. International Journal of Radiation Oncology, Biology, Physics 1981;7:535-41.

48.Harrison LB, Zelefsky MJ, Pfister DG, et al. Detailed quality of life assessment in patients treated with primary radiotherapy for squamous cell cancer of the base of the tongue.[see comment]. Head & Neck 1997;19:169-75.

49.Cooper JS, Fu K, Marks J, Silverman S. Late effects of radiation therapy in the head and neck region. International Journal of Radiation Oncology, Biology, Physics 1995;31:1141-64.

50.Wang CC. Carcinoma of the nasopharynx. In: Wang CC, ed. Radiation Therapy for Head and Neck

Neoplasms: Indications, Techniques, and Results 2ed. Chicago: Year Book Medical Publishers; 1990:261-83.

51.Bailet JW, Mark RJ, Abemayor E, et al. Nasopharyngeal carcinoma: treatment results with primary radiation therapy. Laryngoscope 1992;102:965-72.

52.Hoppe RT, Goffinet DR, Bagshaw MA. Carcinoma of the nasopharynx. Eighteen years' experience with megavoltage radiation therapy. Cancer 1976;37:2605-12.

53.Chu AM, Flynn MB, Achino E, Mendoza EF, Scott RM, Jose B. Irradiation of

nasopharyngeal carcinoma: correlations with treatment factors and stage. International Journal of Radiation Oncology, Biology, Physics 1984;10:2241-9.

54.Teo PM, Ma BB, Chan AT. Radiotherapy for nasopharyngeal carcinoma--transition from two-dimensional to three-dimensional methods. Radiotherapy & Oncology 2004;73:163-72.

55.Sultanem K, Shu HK, Xia P, et al. Three-dimensional intensity-modulated radiotherapy in the treatment of nasopharyngeal carcinoma: the University of California-San Francisco experience. International Journal of Radiation Oncology, Biology, Physics 2000;48:711-22.

56.Liu WS, Su MC, Wu MF, Tseng HC, Kuo HC. Nasopharyngeal carcinoma treated with precision-oriented radiation therapy techniques including intensity-modulated radiotherapy: preliminary results. Kaohsiung Journal of Medical Sciences 2004;20:49-55.

57.Xia P, Fu KK, Wong GW, Akazawa C, Verhey LJ. Comparison of treatment plans involving intensity-modulated radiotherapy for nasopharyngeal carcinoma.[see comment]. International Journal of Radiation Oncology, Biology, Physics 2000;48:329-37.

58.Wolden SL, Zelefsky MJ, Hunt MA, et al. Failure of a 3D conformal boost to improve radiotherapy for nasopharyngeal carcinoma. International Journal of Radiation Oncology, Biology, Physics 2001;49:1229-34.

59.Hunt MA, Zelefsky MJ, Wolden S, et al. Treatment planning and delivery of intensity-modulated radiation therapy for primary nasopharynx cancer. International Journal of Radiation Oncology, Biology, Physics 2001;49:623-32.

60.Lee N, Xia P, Quivey JM, et al. Intensity-modulated radiotherapy in the treatment of nasopharyngeal carcinoma: an update of the UCSF experience.[see comment]. International Journal of Radiation Oncology, Biology, Physics 2002;53:12-22.

61.Kwong DL, Pow EH, Sham JS, et al. Intensity-modulated radiotherapy for early-stage nasopharyngeal carcinoma: a prospective study on disease control and preservation of salivary function. Cancer 2004;101:1584-93.

62.Wolden SL, Chen WC, Pfister DG, Kraus DH, Berry SL, Zelefsky MJ. Intensity-modulated radiation therapy (IMRT) for nasopharynx cancer: update of the Memorial Sloan-Kettering experience. International Journal of Radiation Oncology, Biology, Physics 2006;64:57-62.

63.Pow EH, Kwong DL, McMillan AS, et al. Xerostomia and quality of life after intensity-modulated radiotherapy vs. conventional radiotherapy for early-stage nasopharyngeal carcinoma: initial report on a randomized controlled clinical trial. International Journal of Radiation Oncology, Biology, Physics 2006;66:981-91.

64.Kam M, Leung S, Zee B, et al. Prospective Randomized Study of Intensity-Modulated Radiotherapy on Salivary Gland Function in Early-Stage Nasopharyngeal Carcinoma Patients. Journal of Clinical Oncology 2007;25:4873-9.

65.Kam MK, Teo PM, Chau RM, et al. Treatment of nasopharyngeal carcinoma with intensity-modulated radiotherapy: the Hong Kong experience. International Journal of Radiation Oncology, Biology, Physics 2004;60:1440-50.

66.Presta LG, Chen H, O'Connor SJ, et al. Humanization of an anti-vascular endothelial growth factor monoclonal antibody for the therapy of solid tumors and other disorders. Cancer Research 1997;57:4593-9.

67.Kim KJ, Li B, Winer J, et al. Inhibition of vascular endothelial growth factor-induced angiogenesis suppresses tumour growth in vivo. Nature 1993;362:841-4.

68.Borgstrom P, Gold DP, Hillan KJ, Ferrara N. Importance of VEGF for breast cancer angiogenesis in vivo: implications from intravital microscopy of combination treatments with an anti-VEGF neutralizing monoclonal antibody and doxorubicin. Anticancer Research 1999;19:4203-14.

69.Shaheen RM, Ahmad SA, Liu W, et al. Inhibited growth of colon cancer carcinomatosis by antibodies to vascular endothelial and epidermal growth factor receptors. British Journal of Cancer 2001;85:584-9.

70.Bergers G, Song S, Meyer-Morse N, Bergsland E, Hanahan D. Benefits of targeting both pericytes and endothelial cells in the tumor vasculature with kinase inhibitors.[see comment]. Journal of Clinical Investigation 2003;111:1287-95.

71.Cobleigh MA, Langmuir VK, Sledge GW, et al. A phase I/II dose-escalation trial of bevacizumab in previously treated metastatic breast cancer. Seminars in Oncology 2003;30:117-24.

72.Hurwitz H, Fehrenbacher L, Novotny W, et al. Bevacizumab plus irinotecan, fluorouracil, and leucovorin for metastatic colorectal cancer.[see comment]. New England Journal of Medicine 2004;350:2335-42.

73.Miller KD, Chap LI, Holmes FA, et al. Randomized phase III trial of capecitabine compared with bevacizumab plus capecitabine in patients with previously treated metastatic breast cancer. Journal of Clinical Oncology 2005;23:792-9.

74.Yang JC, Haworth L, Sherry RM, et al. A randomized trial of bevacizumab, an anti-vascular endothelial growth factor antibody, for metastatic renal cancer.[see comment]. New England Journal of Medicine 2003;349:427-34.

75.Chobanian AV, Bakris GL, Black HR, et al. The Seventh Report of the Joint National Committee on Prevention, Detection, Evaluation, and Treatment of High Blood Pressure: the JNC 7 report.[see comment][erratum appears in JAMA. 2003 Jul 9;290(2):197]. JAMA 2003;289:2560-72.

76.Novotny W, Holmgren, E., Griffing, S., et al. Identification of squamous cell histology and central, cavitary tumors as possible risk factors for pulmonary hemorrhage in patients with advanced NSCLC receiving bevacizumab. Proc Am Soc Clin Oncol 2001:20:A1318.

77.Scappaticci FA, Skillings JR, Holden SN, et al. Arterial thromboembolic events in patients with metastatic carcinoma treated with chemotherapy and bevacizumab. Journal of the National Cancer Institute 2007;99:1232-9.

78.Scappaticci FA, Fehrenbacher L, Cartwright T, et al. Surgical wound healing complications in metastatic colorectal cancer patients treated with bevacizumab. Journal of Surgical Oncology 2005;91:173-80.

79.Karp JE, Gojo I, Pili R, et al. Targeting vascular endothelial growth factor for relapsed and refractory adult acute myelogenous leukemias: therapy with sequential 1-beta-d-arabinofuranosylcytosine, mitoxantrone, and bevacizumab. Clinical Cancer Research 2004;10:3577-85.

80.Allen JA, Adlakha A, Bergethon PR. Reversible posterior leukoencephalopathy syndrome after bevacizumab/FOLFIRI regimen for metastatic colon cancer. Archives of Neurology 2006;63:1475-8.

81.Glusker P, Recht L, Lane B. Reversible posterior leukoencephalopathy syndrome and bevacizumab. New England Journal of Medicine 2006;354:980-2; discussion -2.

82.Ozcan C, Wong SJ, Hari P. Reversible posterior leukoencephalopathy syndrome and bevacizumab. New England Journal of Medicine 2006;354:980-2; discussion -2.

83.American Society of Clinical O, Kris MG, Hesketh PJ, et al. American Society of Clinical Oncology guideline for antiemetics in oncology: update 2006. Journal of Clinical Oncology 2006;24:2932-47.

84.Rajendran JG, Krohn KA. Imaging hypoxia and angiogenesis in tumors. Radiologic Clinics of North America 2005;43:169-87.

85.Ziemer LS, Evans SM, Kachur AV, et al. Noninvasive imaging of tumor hypoxia in rats using the 2-nitroimidazole 18F-EF5. European Journal of Nuclear Medicine & Molecular Imaging 2003;30:259-66.

86.Reischl G, Dorow DS, Cullinane C, et al. Imaging of tumor hypoxia with [124I]IAZA in comparison with [18F]FMISO and [18F]FAZA--first small animal PET results. Journal of Pharmacy & Pharmaceutical Sciences 2007;10:203-11.

87.Graham MM, Peterson LM, Link JM, et al. Fluorine-18-fluoromisonidazole radiation dosimetry in imaging studies. Journal of Nuclear Medicine 1997;38:1631-6.

Performance Status Criteria

ECOG Performance Status Scale		Karnofsky Performance Scale	
Grade	Descriptions	Percent	Description
0	Normal activity. Fully active, able to carry on all pre-disease performance without restriction.	100	Normal, no complaints, no evidence of disease.
		90	Able to carry on normal activity; minor signs or symptoms of disease.
1	Symptoms, but ambulatory. Restricted in physically strenuous activity, but ambulatory and able to carry out work of a light or sedentary nature (e.g., light housework, office work).	80	Normal activity with effort; some signs or symptoms of disease.
		70	Cares for self, unable to carry on normal activity or to do active work.
2	In bed <50% of the time. Ambulatory and capable of all self-care, but unable to carry out any work activities. Up and about more than 50% of waking hours.	60	Requires occasional assistance, but is able to care for most of his/her needs.
		50	Requires considerable assistance and frequent medical care.
3	In bed >50% of the time. Capable of only limited self-care, confined to bed or chair more than 50% of waking hours.	40	Disabled, requires special care and assistance.
		30	Severely disabled, hospitalization indicated. Death not imminent.
4	100% bedridden. Completely disabled. Cannot carry on any self-care. Totally confined to bed or chair.	20	Very sick, hospitalization indicated. Death not imminent.
		10	Moribund, fatal processes progressing rapidly.
5	Dead.	0	Dead.

APPENDIX B

Saved a separate document 11/15/07. This is abstract of hypoxia imaging post bevacizumab in GBM. AACR/ EORTC/ NCI meeting October 2007

APPENDIX C: FMISO PET imaging manual: See attached.

APPENDIX D: CTEP MULTICENTER GUIDELINES

If an institution wishes to collaborate with other participating institutions in performing a CTEP sponsored research protocol, then the following guidelines must be followed.

Responsibility of the Protocol Chair

- The Protocol Chair will be the single liaison with the CTEP Protocol and Information Office (PIO). The Protocol Chair is responsible for the coordination, development, submission, and approval of the protocol as well as its subsequent amendments. The protocol must not be rewritten or modified by anyone other than the Protocol Chair. There will be only one version of the protocol, and each participating institution will use that document. The Protocol Chair is responsible for assuring that all participating institutions are using the correct version of the protocol.
- The Protocol Chair is responsible for the overall conduct of the study at all participating institutions and for monitoring its progress. All reporting requirements to CTEP are the responsibility of the Protocol Chair.
- The Protocol Chair is responsible for the timely review of Adverse Events (AE) to assure safety of the patients.
- The Protocol Chair will be responsible for the review of and timely submission of data for study analysis.

Responsibilities of the Coordinating Center

- Each participating institution will have an appropriate assurance on file with the Office for Human Research Protection (OHRP), NIH. The Coordinating Center is responsible for assuring that each participating institution has an OHRP assurance and must maintain copies of IRB approvals from each participating site.
- Prior to the activation of the protocol at each participating institution, an OHRP form 310 (documentation of IRB approval) must be submitted to the CTEP PIO.
- The Coordinating Center is responsible for central patient registration. The

Coordinating Center is responsible for assuring that IRB approval has been obtained at each participating site prior to the first patient registration from that site.

- The Coordinating Center is responsible for the preparation of all submitted data for review by the Protocol Chair.
- The Coordinating Center will maintain documentation of AE reports. There are two options for AE reporting: (1) participating institutions may report directly to CTEP with a copy to the Coordinating Center, or (2) participating institutions report to the Coordinating Center who in turn report to CTEP. The Coordinating Center will submit AE reports to the Protocol Chair for timely review.
- Audits may be accomplished in one of two ways: (1) source documents and research records for selected patients are brought from participating sites to the Coordinating Center for audit, or (2) selected patient records may be audited on-site at participating sites. If the NCI chooses to have an audit at the Coordinating Center, then the Coordinating Center is responsible for having all source documents, research records, all IRB approval documents, NCI Drug Accountability Record forms, patient registration lists, response assessments scans, x-rays, etc. available for the audit.

<u>Inclusion of Multicenter Guidelines in the Protocol</u>

- The protocol must include the following minimum information:

➢ The title page must include the name and address of each participating institution and the name, telephone number and e-mail address of the responsible investigator at each participating institution.

➢ The Coordinating Center must be designated on the title page.

➢ Central registration of patients is required. The procedures for registration must be stated in the protocol.

➢ Data collection forms should be of a common format. Sample forms should be submitted with the protocol. The frequency and timing of data submission forms to the Coordinating Center should be stated.

➢ Describe how AEs will be reported from the participating institutions, either directly to CTEP or through the Coordinating Center.

➢ Describe how Safety Reports and Action Letters from CTEP will be distributed to participating institutions.

<u>Agent Ordering</u>

- Except in very unusual circumstances, each participating institution will order DCTD-supplied investigational agents directly from CTEP. Investigational agents may be ordered by a participating site only after the initial IRB approval for the site has been forwarded by the Coordinating Center to the CTEP PIO.

APPENDIX E:

Radiation Quality Assurance Form

Patient Initials: ____________________ Patient No.: __________ Sex: M ☐ F ☐

Radiotherapy Dept.: ________________ Radiation Oncologist: ______________

DOSE PRESCRIPTION

Target Volume: ____________________ Date of first treatment: ____________________

Dose per Fraction to Prescription Volume (cGy)	
Prescription to which Isodose Surface (e.g. 95%)	
Intended Number of Fractions	
Intended Dose to Prescription Volume	

Maximum Dose per Fraction in the Planning Target Volume	
Minimum Dose per Fraction in the Planning Target Volume	
**Patient's weight pre-tretment**	
**Patient's weight post-treatment**	

Planning System	
Treatment Machine	
Patient Position	

	IMRT ☐<br>***OR***<br>3D ☐	Form of IMRT<br>SMLC (step & shoot):_________<br>DMLC (sliding window):_______<br>Serial tomotherapy (MIMiC):_________________<br>Other:______________________	Integrated boost ☐<br>***OR***<br>Sequential boost ☐	Supraclavicular field matching:<br>Yes ☐<br>No ☐

**List Names Of Target Volumes Corresponding To Those On RT-1 Forms, Record Boost Volumes Separately**			
Names of Target Volume (i.e. PTV1, Chest)			
Date of First Treatment to the Target Volume			
Number of Treatments			
Date of Last Treatment			
Total Dose To Prescription Point			
Number of Fields			
Beam Energy			
Monitor Unit/Fraction			
**Critical Structure**	**Max Dose (Gy)**	**Critical Structure**	**Max Dose (Gy)**
**A.** Brainstem	54	**F .** Optic nerves	54
**B.** Spinal cord	45	**G.** Optic chiasms	54
**C.** Eyes	50	**H.** Other	
**D.** Brachial plexus	66	**I.** Other	
**E.** Mandible	70	**J.** Other	
**Interruptions**			
**From:**	**To:**	**Reason:**	
**From:**	**To:**	**Reason:**	
**From:**	**To:**	**Reason:**	
**From:**	**To:**	**Reason:**	
**Off Protocol Therapy**			
**Date:**	**Reason:**		
**Discontinued Radiotherapy**			
**Date:**	**Reason:**		

Appendix F Registration and data submission forms: in development
