# Supplementary material for: In search for optimal induction chemotherapy for advanced nasopharyngeal cancer: Standard dosing of Docetaxel, Platinum, and 5-Fluorouracil (TPF) followed by chemoradiation
Source: PLoS One. 2023 Feb 2;18(2):e0276651. doi: 10.1371/journal.pone.0276651 (PMC9894485; doi:10.1371/journal.pone.0276651)

Appendix A. Percentage of intended cumulative concurrent platinum dose received vs Number of M0 patients.

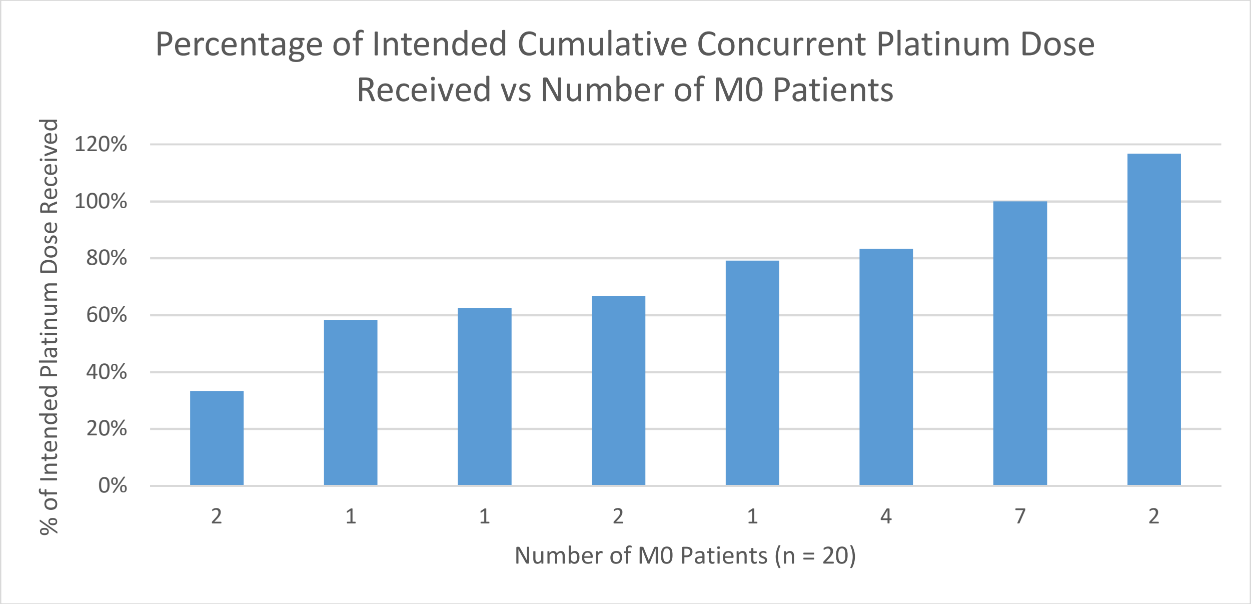

Appendix B. Percentage of intended cumulative concurrent platinum dose received vs Number of M1 patients.

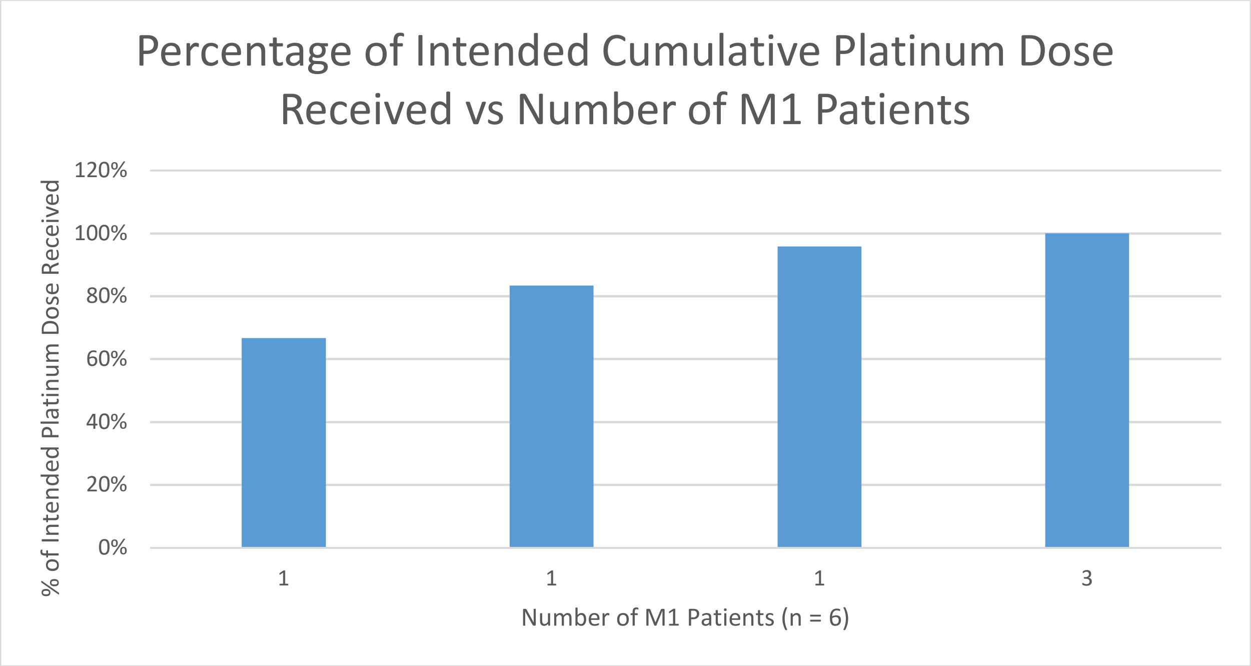

Supplement: S1 Appendix — (PDF) [file pone.0276651.s005.pdf]
